# Covalent organic frameworks-supported copper bismuth oxide nanoparticles as an efficient and green photocatalyst for benzyl alcohol oxidation

**DOI:** 10.1038/s41598-025-25368-8

**Published:** 2025-11-21

**Authors:** Sanaz HajimohamadzadehTorkambour, Masoumeh Jadidi Nejad, Farzane Pazoki, Akbar Heydari

**Affiliations:** 1 Chemistry Department, Tarbiat Modares University, P.O. Box 14155-4838, Tehran, Iran; 2 Department of Chemistry, Isfahan University of Technology, P.O. Box 84156-83111, Isfahan, Iran

**Keywords:** Photocatalyst, Covalent organic frameworks (COFs), Benzyl alcohol, Benzaldehyde, Oxidation reaction, Green chemistry, Chemistry, Materials science

## Abstract

This work presents a sustainable and eco-friendly method using covalent organic frameworks (COFs) under visible light to selectively photocatalyze the oxidation of aromatic alcohols into beneficial aromatic aldehyde compounds. Under mild conditions, this work investigated a composite of CuBi₂O₄ and CTFs (CTF-CuBi₂O₄) as a heterogeneous photocatalyst for the oxidation of benzyl alcohols to their respective benzaldehydes using TBHP as the oxidizing agent. Several microscopic and spectroscopic techniques, including FT-IR, PXRD, SEM, TEM, EDAX, BET, TGA, UV-DRS, Matt-Schottky, and PL, were used to investigate the physical and structural features of this photocatalyst. Moreover, mass spectroscopy, ^1^H NMR, and^13^C NMR were the techniques applied to verify the corresponding benzaldehydes’ structures. The target products can be synthesized in moderate to high yields under appropriate reaction conditions. This technology offers advantages such as diminished reaction time and temperature (room temperature), utilization of visible light as a renewable and eco-friendly energy source, moderate reaction conditions, application of a recyclable catalyst, enhanced selectivity, and minimized undesirable byproducts. Additionally, the stability and recyclability of the catalyst were examined under ideal conditions, revealing that it can be reversibly reused for up to 6 cycles without substantial loss of activity.

**Supplementary Information:**

The online version contains supplementary material available at 10.1038/s41598-025-25368-8.

## Introduction

One of the fundamental transformations in organic synthesis is the oxidation of benzyl alcohols to their corresponding aldehydes^1^, providing key intermediates that are widely used in pharmaceuticals, fine chemicals, fragrances, and industrial materials^2–7^. Conventional oxidation procedures often require high reaction temperatures, pressures, and high-valent metal reagents as oxidizing agents^8–10^. Hazardous byproducts from stoichiometric oxidants, such as potassium permanganate, cobalt ferrite, sodium hypochlorite, and nitric acid, pose significant environmental and safety concerns^11–13^. Consequently, creating a green and sustainable oxidation method, such as photocatalytic oxidation of benzyl alcohol, utilizing oxidants like oxygen (O₂) or tert-butyl hydroperoxide (TBHP), has been a top study priority^14–16^. TBHP is particularly effective among these oxidants as it generates reactive oxygen species (ROS) upon light irradiation, independent of external oxygen sources^17,18^. The choice of oxidant depends strongly on the photocatalyst’s conduction and valence band positions, as only oxidants with suitable redox potentials can be efficiently activated^19,20^. In some reported cases, alcohol oxidation can proceed without an external oxidant, but this typically requires noble metals such as Ni, Pd, Au, Pt, or Ru. These metals can directly mediate α-C–H bond cleavage in benzyl alcohol, enabling dehydrogenation and oxidation in the absence of sacrificial oxidants^21,22^.

The predominant method for industrial synthesis of benzaldehyde has relied on chlorination followed by successive hydrolysis. Still, this route is hindered by the costs of wastewater treatment, contamination, and equipment corrosion^23–25^. Other approaches, such as aerobic oxidation of toluene with homogeneous catalysts, suffer from a lack of selectivity^26^. Benzaldehyde was produced through selective oxidation or reduction using various starting materials, among which benzyl alcohol was particularly appealing because selective oxidation could facilitate the activation of the transition at relatively low temperatures^27,28^.

In contrast, photocatalysis offers a sustainable route by driving redox reactions under mild conditions using light energy^29,30^. Following the pioneering work of Fujishima and Honda^31^, researchers have explored visible-light-driven photocatalysts beyond UV-active semiconductors, such as TiO₂ and ZnO^32–34^. Research on semiconductors with tiny band gaps, mostly bismuth- and copper-based materials, has been driven by strong absorption in the visible-light spectrum to enhance solar energy utilization^35,36^. Among these materials, copper bismuthate (CuBi₂O₄) has emerged as a promising p-type semiconductor with a narrow band gap of 1.5–1.8 eV, facilitating effective visible-light absorption^37,38^. Excellent surface catalytic activity, photostability, and optical characteristics have made CuBi₂O₄ of interest^37^. However, pure CuBi₂O₄ has a weak chemical affinity for substrates and elevated recombination rates of photogenerated charge carriers, hence constraining its overall photocatalytic effectiveness^39^. Strategies such as noble metal deposition with heterostructure formation have improved the photocatalytic performance of CuBi₂O₄; however, high costs restrict its scalability^40,41^. For example, recent studies have shown that Au-decorated CuBi₂O₄ microrods exhibit enhanced stability and photocatalytic efficiency, although the reliance on noble metals remains a limitation^42^. Moreover, composite photocatalysts such as CuBi₂O₄/WO₃, CuBi₂O₄/Bi₂WO₆, and CuBi₂O₄/Ag₃PO₄ have demonstrated higher photocatalytic performance than single-component CuBi₂O₄.^43–45^ Furthermore, recent reviews emphasize that CuBi₂O₄-based heterostructures consistently exhibit improved photocatalytic efficiencies for pollutant degradation and redox transformations, confirming the versatility of this material^46^.

In addition, semiconductor catalysts like covalent organic frameworks (COFs), g-C₃N₄^47^, metal halide perovskites, and metal–organic frameworks (MOFs)^48^ have also been employed in photocatalytic benzyl alcohol oxidations due to their tunable electronic structures, stability, and low toxicity^49^. Covalent triazine frameworks (CTFs), in particular, offer high surface area, thermal and chemical stability, and efficient charge transport^50–52^. Their π-conjugated, nitrogen-rich structures promote visible-light absorption, though synthesis at high temperatures can induce defects^53,54^. Recent reviews have highlighted the potential of COFs/CTFs for photocatalytic transformations, particularly the selective oxidation of alcohols, due to their ordered porosity and tunable band structures^55,56^.

The combination of CuBi₂O₄ with CTFs presents a promising strategy for developing hybrid photocatalysts that leverage the advantages of both components. CuBi₂O₄ exhibits strong visible-light absorption and good surface catalytic properties, while CTFs enhance charge separation and reduce electron-hole recombination, resulting in overall improved photocatalytic performance.

In this study, we report a novel hybrid photocatalyst composed of CuBi₂O₄ and CTFs, which enables the efficient and selective oxidation of benzyl alcohols to benzaldehydes under visible-light irradiation. A significant advantage of this system is its ability to operate at room temperature, and it also exhibits enhanced photocatalytic activity and faster reaction rates compared to traditional methods. Additionally, the catalyst is sustainable, as it is heterogeneous, allowing for easy recovery and reuse. Moreover, this hybrid system exhibits pronounced visible-light absorption and efficient charge transport, which helps minimize electron-hole recombination and enhances overall efficiency. This approach ensures selective oxidation of benzyl alcohols to benzaldehydes without promoting excessive oxidation to benzoic acids. The method also aligns with green and sustainable principles by employing a CTF and harnessing visible light as the primary energy source, thus reducing the reliance on harmful chemicals. Synthesis of the catalyst itself is straightforward and scalable. Moreover, the system is also well-suited for long-term use, as it remains stable over multiple cycles with minimal loss in activity.

## Materials and methods

This section examines the methodologies employed in the synthesis and characterization process. All the materials were purchased from Merck (Germany) and Aldrich (China). Thin-layer chromatography was used to monitor the progress of the reaction. TLC was conducted on glass plates using silica-gel 60 F-254 as the matrix. Infrared (IR) spectra within the range of 400–4000 cm⁻¹ were recorded using KBr pellets on a Nicolet IR100 instrument. X-ray diffraction (XRD) analysis was performed at room temperature utilizing a Philips X-pert 1710 equipped with monochromated Cu Kα radiation, covering a 2θ range of 10° to 80°. A TESCAN MIRA III Field Emission Scanning Electron Microscope (FE-SEM) was used to examine the morphology and size of the particles. EDAX analysis was carried out to determine the elemental composition of the catalysts. Thermal gravimetric analysis (TGA) was conducted with a thermal analyzer, operating at a heating rate of 20 °C min⁻¹ over a temperature range of 25–800 °C in an air environment. Transmission electron microscopy (TEM) imaging was performed using a Philips CM 120 at an accelerating voltage of 120 kV. The Brunauer-Emmett-Teller (BET) method was employed to determine the nanocatalyst’s surface area through nitrogen gas physisorption, using the BELSORP MINI II instrument at 77 K. UV-visible diffuse reflectance spectroscopy (DRS) measurements were conducted using a Shimadzu UV-2550 spectrophotometer within the 200–800 nm wavelength range. For the Matt-Schottky experiment, a Na₂SO₄ (0.5 M) electrolyte solution and a calomel reference electrode were used. Additionally, a Shimadzu RF6000 fluorescence spectrophotometer was utilized to analyze the photoluminescence (PL) spectra. The ¹H NMR and ¹³C NMR spectra were obtained using a Bruker DRX-300 Avance spectrometer at 300 and 75 MHz, respectively.

### Synthesis of CTF

Firstly, 0.66 gr of melamine (5.2 mmol), 0.5 gr of triethylamine (5 mmol), and 10 mL of DMF were mixed by ultrasonication within a round-bottom flask for 10 min (solution 1); then, 0.92 gr of trichlorotriazine (TCT) (5mmol) was added to solution 1 and was sonicated for 30–40 min. Next, the mixture was conveyed into a Teflon-lined autoclave for 24 h at 120 °C. Then, the solution was cooled to room temperature, and a white precipitate was separated by centrifuging and eluted with dimethylformamide and ethanol several times. Finally, it was dried in the oven at 80 °C to acquire CTF^57^.

### Synthesis of CTF-CuBi_2_O_4_

**Fig. 1:**
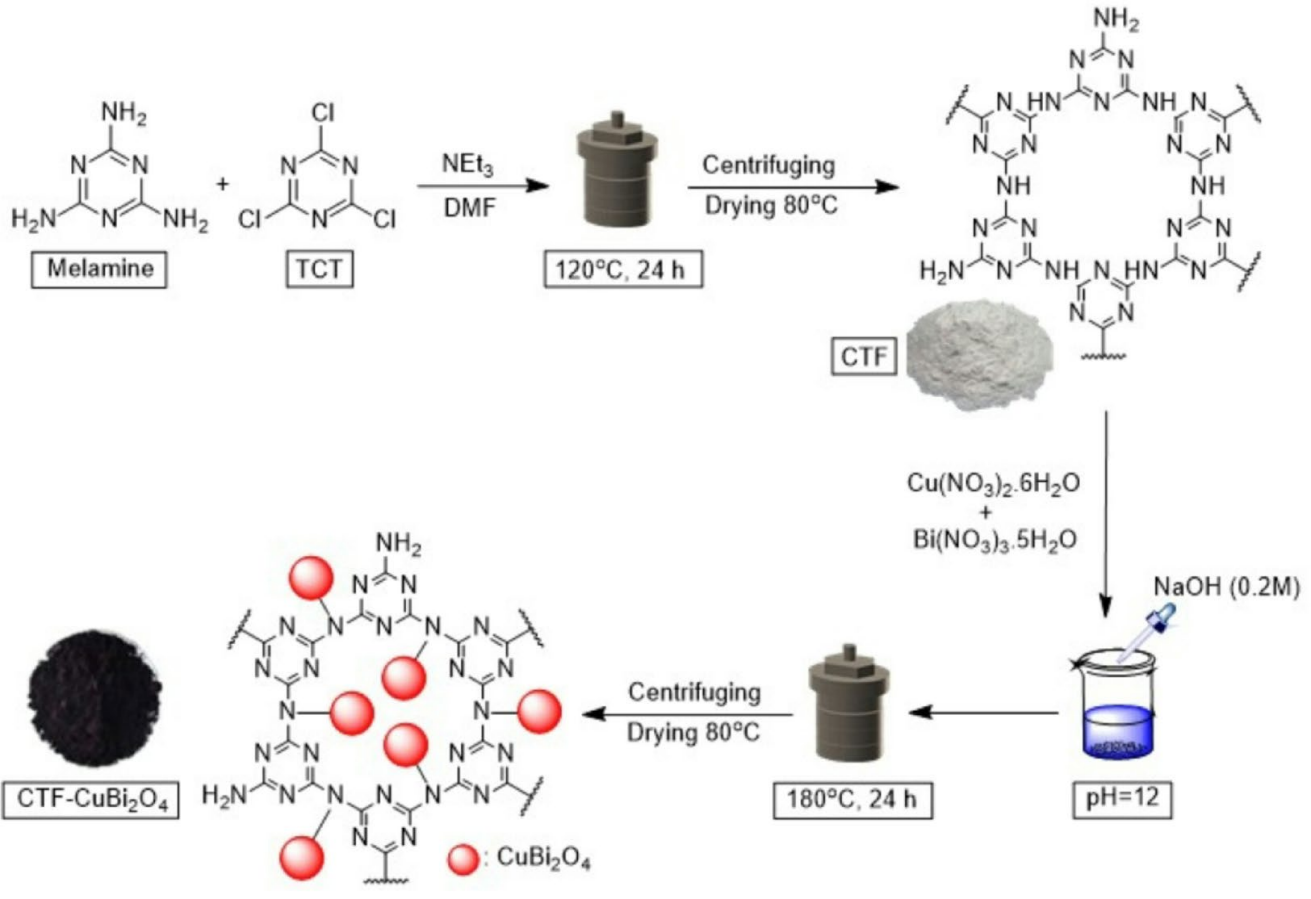
Schematic diagram of the synthesis of CTF-CuBi_2_O_4_.

0.44 gr of copper (II) nitrate hexahydrate (1.5 mmol) was solved in 10 mL of deionized (DI) water within a round-bottom flask by ultrasonic for 10 min (solution 1); then, 1.4 gr of bismuth (III) nitrate pentahydrate (3 mmol) was solved in 10 mL of DI water by ultrasonic for 10 min (solution 2). Afterward, solution 1 and solution 2 were sonicated for 30 min. After that, 0.6 gr of CTF was added to the previous mixture, and the homogenous solution was mixed by ultrasonication for 30 min. A solution of sodium hydroxide (0.2 M) (20 mL, 0.16 gr) was added to adjust pH = 12. Then, the mixture was placed in an autoclave for 24 h at 180 °C. In the next step, the black solid product was separated by centrifuge and washed with DI water and ethanol several times. Finally, the product was dried in the oven at 80 °C to acquire CTF-CuBi_2_O_4_. All of the processes are shown in Fig. 1.

### Activity evaluation

The tests were conducted under visible light illumination using a 20 W blue LED lamp as the light source. A white LED with 400–750 nm wavelength was used, while a 460 nm wavelength was applied for the blue LED. The light intensity was 1.6 × 10⁻² W·cm⁻². A reaction mixture containing 1 mmol of benzyl alcohol, 3 mmol of TBHP (tert-butyl hydroperoxide, 70% aqueous solution), and 30 mg of catalyst was prepared in 3 mL of n-hexane within a test tube. This mixture was stirred for 3 h under LED. The progress of the reaction was evaluated by thin-layer chromatography. After completion of the reaction, the solution was cooled to room temperature; then, the catalyst was separated by centrifuge and extracted with ethyl acetate. Finally, the product had to be isolated by column chromatography (ethyl acetate and hexane in a ratio of 3:2) on silica gel. Also, it was mentioned that the characterizations of the isolated products were based on using^13^C NMR, ^1^H NMR, and Mass spectroscopy.

### Reusing experiments

Six-cycle experiments were executed on the catalyst to test its reusability and photostability. After each experiment, the photocatalyst was gathered, and the samples were purged with DI water and ethanol and then dried at 80 °C. The catalyst was reused under the same reaction conditions, and after each use, the transformation rate of the reactants was measured. Finally, the photocatalyst was characterized by different analyses.

## Results and discussion

### Characterization of the catalyst

**FT-IR analysis**.

**Fig. 2:**
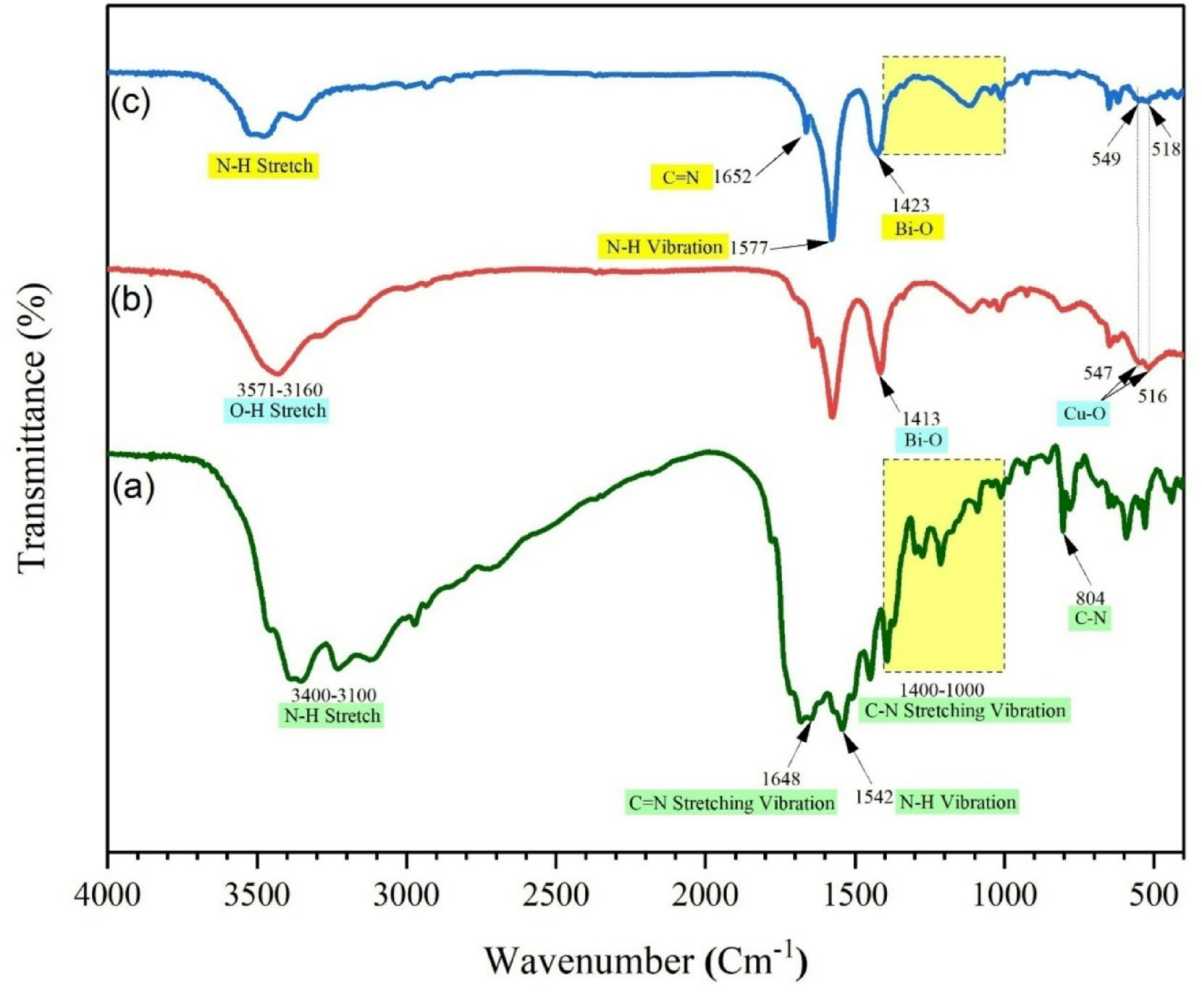
FT-IR spectrum of CTF (a), CuBi_2_O_4_ (b), and CTF-CuBi_2_O_4_ (c).

FT-IR spectroscopy was employed to identify structural features and functional groups. The spectra of CTF (Fig. 2a) exhibit a broad band at 3400–3100 cm⁻¹, corresponding to N–H stretching vibrations, while the peak at 1648 cm⁻¹ is assigned to the stretching vibration of C = N bonds. The band at 1542 cm⁻¹ is attributed to N–H bending vibrations, and the region between 1400 and 1000 cm⁻¹ corresponds to symmetric stretching vibrations of C–N and C = N groups within the triazine ring. A distinct peak at 804 cm⁻¹ represents the out-of-plane breathing mode of the triazine unit, confirming the successful formation of the framework. For CuBi₂O₄ (Fig. 2b), the broad band at 3571–3160 cm⁻¹ is assigned to O–H stretching vibrations of surface-adsorbed water. The characteristic metal–oxygen vibrations are observed at 547 cm⁻¹ (Cu–O) and 516 cm⁻¹ (additional Cu–O mode). In addition, a band at 1413 cm⁻¹ is attributed to Bi–O stretching vibrations. In the spectrum of the CTF–CuBi₂O₄ composite (Fig. 2c), the characteristic bands of both components are present, indicating successful integration. The C = N stretching vibration at 1652 cm⁻¹ and the C–N vibrations (1400–1000 cm⁻¹) are retained from CTF, while the Cu–O stretching at 549 and 518 cm⁻¹ and Bi–O stretching at 1423 cm⁻¹ confirm the presence of CuBi₂O₄. The slight shifts and reduced intensities compared to the pristine materials suggest strong interfacial interaction between CTF and CuBi₂O₄ in the composite.

**XRD analysis**.

**Fig. 3:**
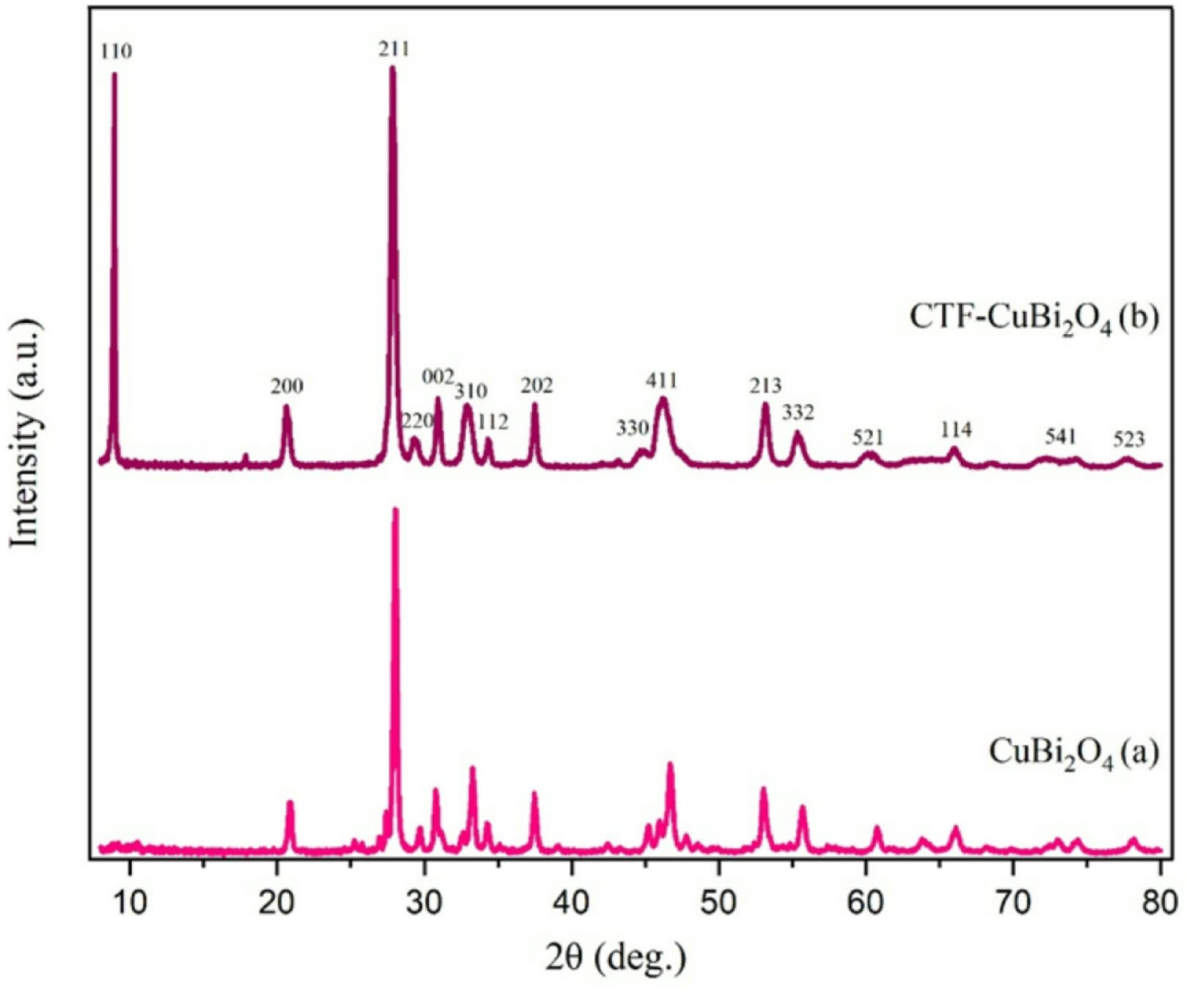
PXRD of CuBi_2_O_4_ (a) and CTF-CuBi_2_O_4_ (b).

The XRD patterns of CuBi_2_O_4_ and CTF-CuBi_2_O_4_ are exhibited in Fig. 3. The broad peak was attributed to (110). This peak is characteristic of the amorphous carbon nature of CTF. The figure shows prominent peaks at 20°, 27°, 29.59°, 30°, 32°, 34°, 37°, 44°, 47°, 53°, 55.95°, 60.8°, 65.6°, 73°, and 77.8° which correspond to planes (200), (211), (220), (002), (310), (112), (202), (330), (411), (213), (332), (521), (114), (541), and (523), respectively. The diffraction peaks of CuBi₂O₄ matched well with the standard tetragonal phase pattern (JCPDS No. 72–0493), verifying the formation of a pure crystalline phase. This observation is consistent with earlier studies on hydrothermally synthesized CuBi₂O₄^58^.

**SEM analysis**.

**Fig. 4:**
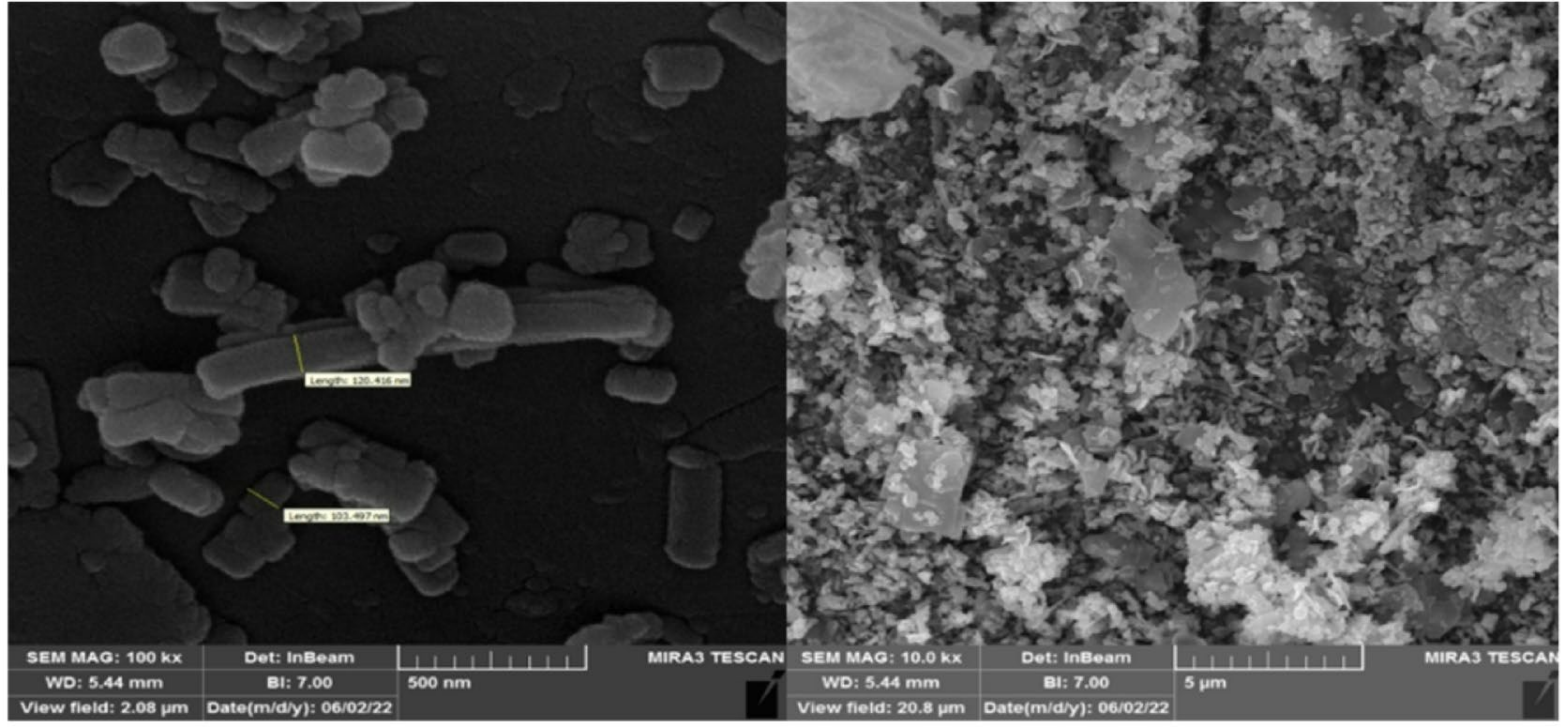
FE-SEM of CTF-CuBi_2_O_4_.

The morphology distribution, surface, and powder size of nanoparticles CTF-CuBi_2_O_4_ were investigated by using FE-SEM, as shown in Fig. 4. The CTF consists of a significant and irregular layered structure decorated with a column nanorod cluster of CuBi_2_O_4_. The size of CuBi_2_O_4_ nanoparticles is from 103 to 120 nm.

**TEM analysis**.

**Fig. 5:**
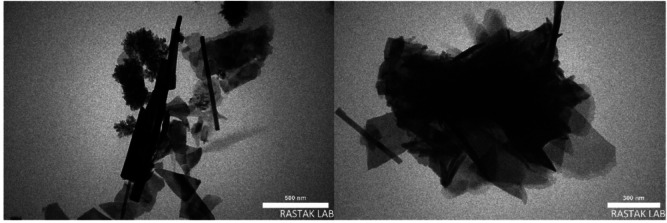
TEM images of CTF-CuBi_2_O_4_.

The morphology and particle size of the CTF-CuBi_2_O_4_ nanoparticles are displayed in Fig. 5, and they indicate a column nanorod cluster. The smallest nanoparticles are determined at about 103 nm.

**EDS analysis**.

**Fig. 6:**
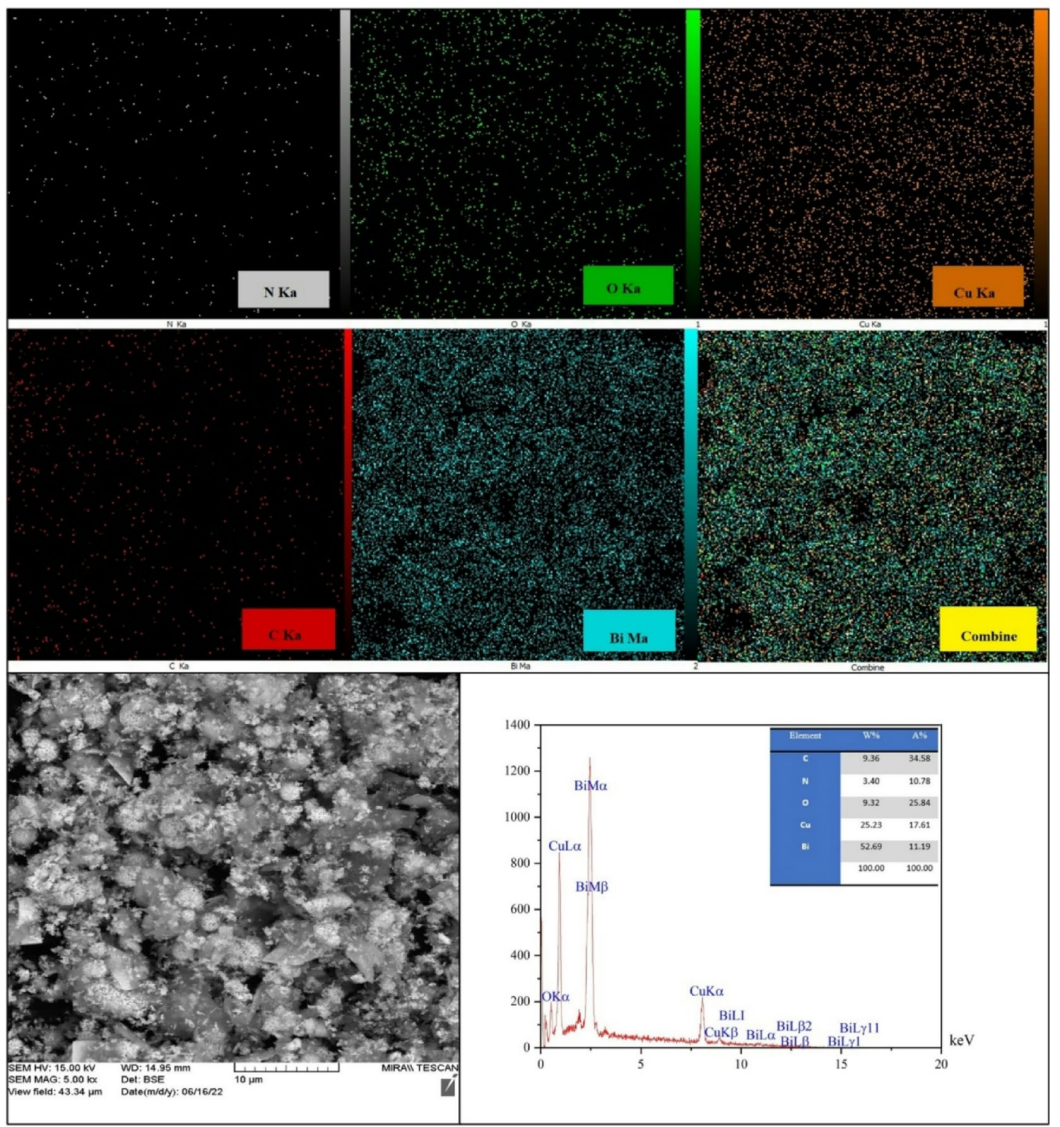
EDAX element mapping images and analysis of CTF-CuBi_2_O_4_ and SEM/EDS.

EDS mapping was carried out to investigate the distribution of the elements in the nanoparticles. The high dispersive of five elements (N, O, Cu, C, and Bi) is shown in Fig. 6. The energy dispersive x-ray (EDAX) spectrum confirmed the type of present elements in the nanoparticles of CTF-CuBi_2_O_4_ and the result is depicted in Fig. 6. The sample of CuBi_2_O_4_ contains the elements of O, Bi, and Cu in the structure.

**N**_**2**_ **adsorption-desorption isotherm curves**.

**Fig. 7:**
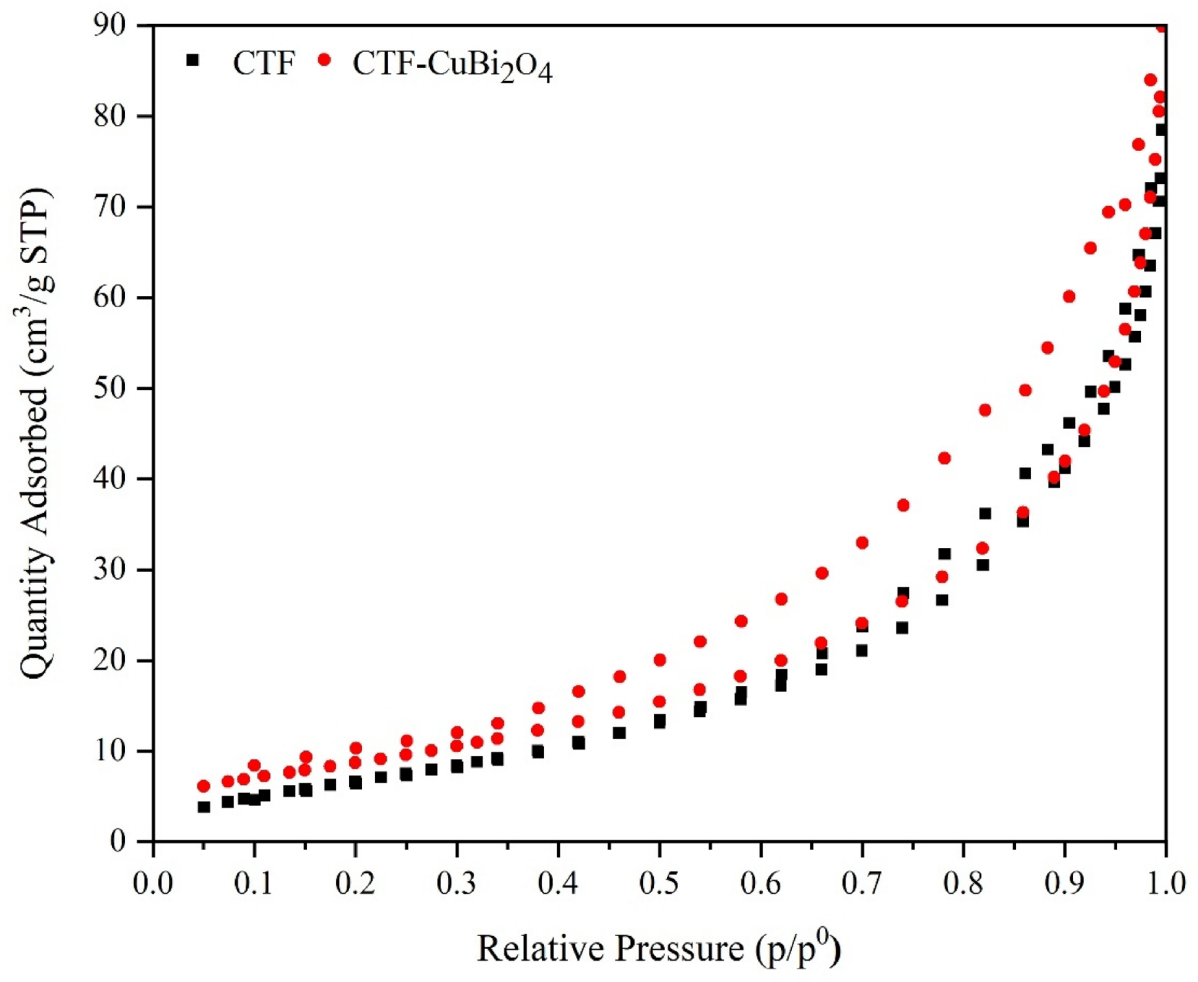
BET analysis of CTF and CTF-CuBi_2_O_4_.

Surface area and porosity of CTF, CuBi_2_O_4,_ and CTF-CuBi_2_O_4_ were investigated by using the N_2_ adsorption-desorption isotherm and BET at 77 K (in Fig. 7). The BET surface areas of the CTF, CuBi_2_O_4,_ and CTF-CuBi_2_O_4,_ according to calculations, are 28.20, 8.23, and 33.37 m^2^g^− 1^, respectively. These results demonstrate that while pristine CuBi₂O₄ exhibits a relatively low surface area, incorporation into the CTF framework markedly enhances the accessible surface area of the hybrid material. To further assess the pore structure, BJH analysis was applied. The calculated average pore sizes were 5.92 nm (CTF), 1.21 nm (CuBi₂O₄), and 5.80 nm (CTF–CuBi₂O₄), indicating the predominantly mesoporous character of the framework materials and the microporous nature of pure CuBi₂O₄. The pore capacities were found to be 0.06 cm³g⁻¹, 0.05 cm³g⁻¹, and 0.07 cm³g⁻¹, respectively, as shown in Table 1. These data suggest that the integration of CuBi₂O₄ nanoparticles into the porous CTF scaffold prevents pore blockage and maintains mesoporosity, while also slightly increasing pore volume. This enhancement is beneficial for photocatalysis, as it improves substrate diffusion and provides more accessible active sites.

**Table 1:** BET and BJH date for CTF, CuBi_2_O_4_, and CTF-CuBi_2_O_4_.

Entry	BET Surface Area (m^2^/g)	Average Pore Size (nm)	Pore Capacity (Cm^3^/g)
CTF	28.20	5.92	0.06
CuBi_2_O_4_	8.23	1.21	0.05
CTF-CuBi_2_O_4_	33.37	5.80	0.07

**TGA analysis**.

**Fig. 8:**
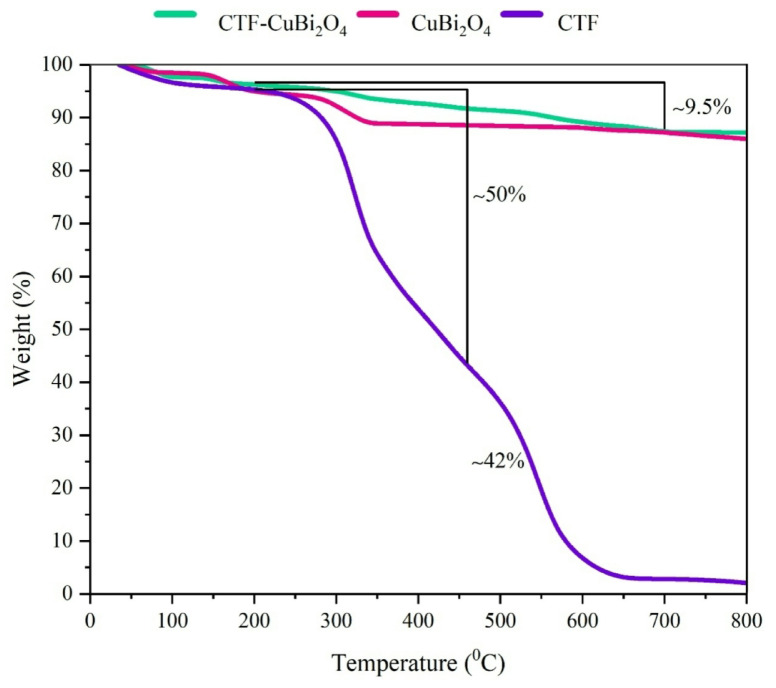
TGA curves of CTF, CuBi_2_O_4_, and CTF-CuBi_2_O_4_.

TGA was investigated for thermal performance and thermostability of CTF, CuBi_2_O_4_, and CTF-CuBi_2_O_4_ in an air atmosphere at a 20 °C·min^− 1^ heating rate in Fig. 8. As shown in the figure, the initial degradation of pure CTF commenced below 200 °C, resulting in approximately 5% loss due to evaporating moisture and solvents. Figure 8 indicates that CTF underwent degradation in two primary stages at the elevated temperature of 200 °C. The initial mass loss of approximately 50% occurred between 200 and 460 °C, followed by a second mass drop of roughly 42% between 480 and 650 °C. After oxidation, the residual CTF is approximately 3%. The primary causes of this are fragmentation of aromatic rings, the cleavage of the principal chains, and carbon oxidation. The curve of bare CuBi_2_O_4_ shows a weight decrease of about 5% below 200 °C, mainly due to the evaporation of entrapped and absorbed water. According to the CTF-CuBi_2_O_4_ curve, the final photocatalyst has some weight loss steps. The first and second weight losses, approximately 4% below 200 °C, correspond to dehydration and the removal of organic solvent. The next decomposition steps, observed at temperatures ranging from 200 °C to 700 °C (approximately 9.5%), are attributed to the degradation of organic ligands, revealing the tremendous thermal stability of the nanoparticles in these results (total weight loss is approximately 12.5%).

**UV-vis DRS analysis**.

**Fig. 9:**
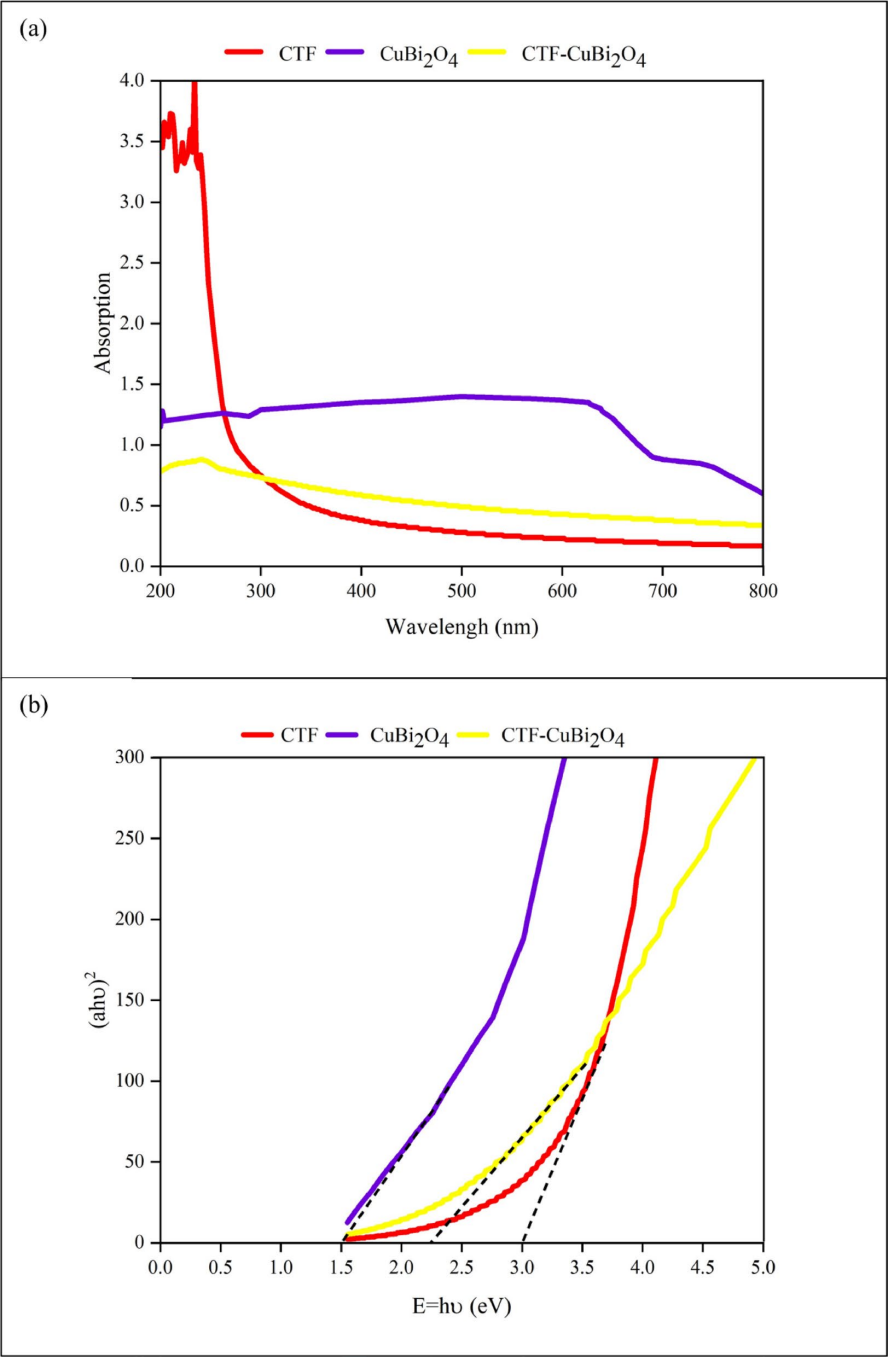
Uv-vis DRS (a) and Kubelka-Munk plot (b) of CTF, CuBi_2_O_4_, and CTF-CuBi_2_O_4_.

The light absorption characteristics of the materials in their prepared state were evaluated by collecting UV-vis spectra. Pure CTF (Fig. 9a, red curve) exhibits typical absorption margins at approximately 300 nm in the UV range and an estimated band energy of 3.0 eV (Fig. 9b, red curve). While the absorption edge is prolonged up to 730 nm for pure CuBi_2_O_4_ (Fig. 9a, the purple curve), the band energy is estimated to be around 1.5 eV (shown in Fig. 9b, the purple curve). The CTF-CuBi_2_O_4_ composite (Fig. 9a, Yellow curve) shows a clear red shift of the absorption band when CTF is coupled with CuBi_2_O_4_. This suggests that CuBi_2_O_4_ plays a significant role in harnessing sunlight and may help to increase the photocatalytic activity for the subsequent target reactions.

Mott-Schottky measurements of CTF (see Fig. S1) were performed at 1000 Hz in 0.5 M Na₂SO₄ to verify its semiconductor characteristics and band structure. In these measurements, a positive slope in the Mott-Schottky plot indicates n-type behavior, whereas a negative slope confirms p-type behavior^59,60^. For p-type semiconductors, the Fermi level ($\:{E}_{F}$) typically lies close to the valence band (VB) at around 0.10 eV more positive, while for n-type semiconductors, the conduction band (CB) is approximately 0.10 eV lower. Consequently, CTF’s CB edge at about − 0.82 eV relative to the saturated calomel electrode (SCE). Because the standard hydrogen electrode (NHE) can be related to SCE by $\:{E}_{NHE}\:=\:{E}_{SCE}\:+\:0.24$^61,62^, the CTF conduction and VBs are found to be − 0.58 eV and 2.42 eV, respectively, on the NHE scale.

Moreover, it is possible to estimate the band-edge positions theoretically using an empirical formula originally developed for CuBi₂O₄^63,64^. The conduction band energy ($\:{E}_{CB}$) can be calculated via:

1 $$\:{E}_{CB}=X-\:{E}^{e}-\:0.5{E}_{g}$$

2 $$\:{E}_{VB}={E}_{g}\:+\:{E}_{CB}$$

Where E^e^ is the energy of free electrons on the hydrogen scale (4.5 eV), E_g_ is the semiconductor’s band gap (1.5 eV), X is the semiconductor’s absolute electronegativity (4.76), and E_CB_ is the conduction band energy. CuBi_2_O_4_ was determined to possess E_CB_ and E_VB_ values of − 0.49 and 1.01 eV, respectively.

A p-n junction structure is established when p-type CuBi_2_O_4_ contacts n-type CTF, attributable to the conduction band edge and the lower Fermi level of CuBi_2_O_4_ compared to CTF. The electrons move from CTF (higher level) to CuBi_2_O_4_ (lower level) because of this energy level difference until the Fermi levels of CTF and CuBi_2_O_4_ equalize. As the energy bands of CTF shift downward relative to its Fermi level, those of CuBi₂O₄ move upward. Consequently, the conduction band edge of CuBi₂O₄ is positioned higher than that of CTF. In contrast, the valence band edge of CTF is inferior, attributable to the establishment of equilibrium and the generation of an internal electric field at the p-n heterojunction interface. The photoinduced electrons on the CB of CuBi_2_O_4_ moved to the more positive CB of CTF^61–64^ when the composite photocatalyst was exposed to visible light, as seen in Fig. 9. This caused the hydroxyl anion (OH⁻) and tert-butoxide radical (tBuO^•^) to be produced. In contrast, the holes on the VB of CTF moved to that of CuBi_2_O_4_.

**PL spectroscopy**.

**Fig. 10:**
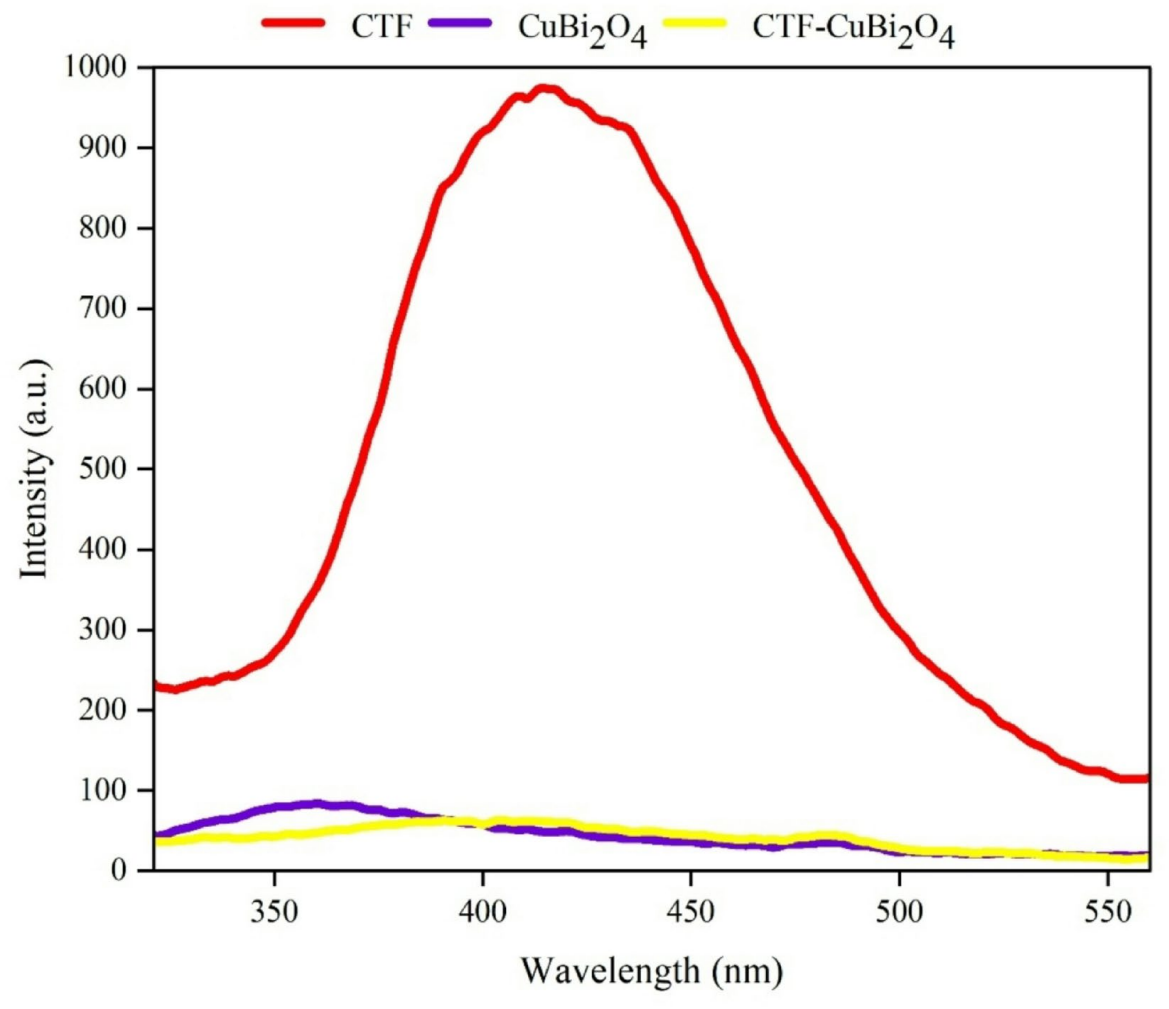
PL spectroscopy of CTF, CuBi_2_O_4_, and CTF-CuBi_2_O_4_.

Photoluminescence (PL) measurements were carried out to examine the efficiency of charge-carrier separation and the corresponding recombination rate, noting that higher PL emission intensities generally correlate with shorter carrier lifetimes. The resulting PL spectra for CTF, CuBi₂O₄, and the CTF-CuBi₂O₄ composite, shown in Fig. 10, reveal a prominent emission band at an excitation wavelength of 300 nm in the CTF samples. This observation indicates a relatively high rate of electron-hole pair recombination. By contrast, the CTF-CuBi₂O₄ photocatalyst exhibited the weakest emission signal among all tested samples, implying that photogenerated electron-hole pairs were more effectively separated and transported.

## Photocatalytic activity

### Effect of light wavelength

The reaction was investigated under different light wavelengths and was studied with and without varying quantities of the photocatalyst (Table 2). The absence of light (Table 2, entry 8) resulted in no production, indicating that light irradiation is crucial in the reaction’s progression. Product yield increased significantly at shorter wavelengths (Table 2, entries 4–7), as higher-energy photons more effectively activated the photocatalyst and promoted electron–hole pair generation. longer wavelengths, the energy was insufficient to activate the photocatalyst.

The reaction was conducted using varying amounts of the photocatalyst (Table 2, entries 1–3). The optimal outcome was attained with 30 mg of photocatalyst. Reducing quantities affected product efficiency, whereas increasing amounts did not improve yield. Without a catalyst (Table 2, entry 9), the reaction did not proceed, highlighting the indispensable role of the photocatalyst in facilitating benzyl alcohol oxidation.

**Table 2:** Screening of light Wavelength.

Entry	Amount of photocatalyst (g)	Light	Yield (%)
1	CTF-CuBi_2_O_4_ (0.1)	blue	96
2	CTF-CuBi_2_O_4_ (0.02)	blue	85
3	CTF-CuBi_2_O_4_ (0.03)	blue	96
4	CTF-CuBi_2_O_4_ (0.03)	white	81
5	CTF-CuBi_2_O_4_ (0.03)	red	56
6	CTF-CuBi_2_O_4_ (0.03)	sun	78
7	CTF-CuBi_2_O_4_ (0.03)	dark	11
8	-	blue	trace

### Effect of light intensity

**Fig. 11:**
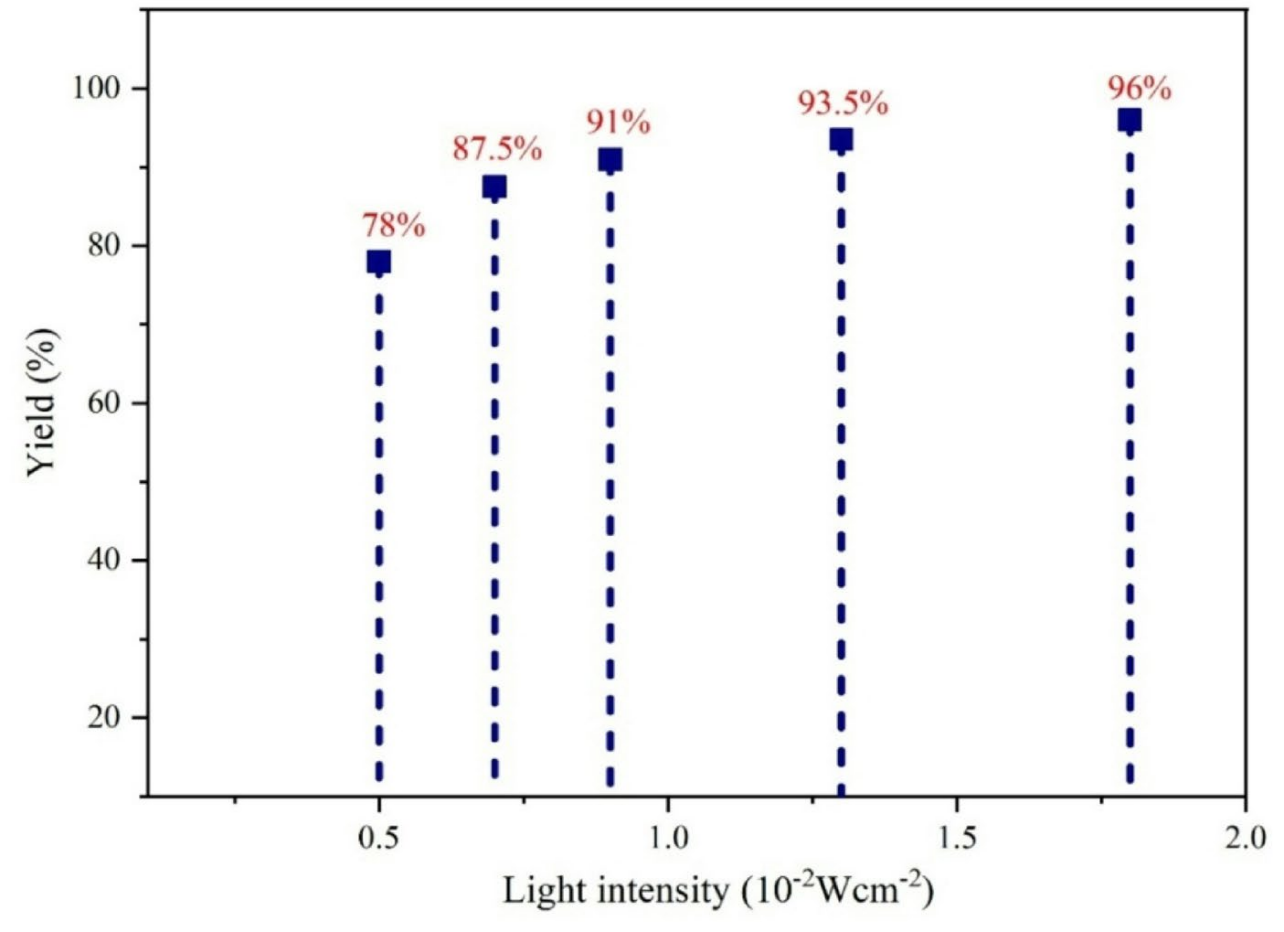
The influence of blue light intensity.

The influence of blue light intensity on the progression of the reaction was examined in Fig. 11. The augmentation of light intensity elevated the product yield due to improved transfer of photogenerated carriers and enhanced catalytic reactivity.

### Catalytic activity

After the characterization of the photocatalyst, we examined its catalytic efficacy in the oxidation of benzyl alcohols, as presented in Table 3. The oxidation of 4-methoxybenzyl alcohol to benzaldehyde was chosen as the model reaction, and various essential parameters, including solvent, oxidant, oxidant quantity, reaction duration, and catalyst type, were used to optimize reaction conditions.

**Fig. 12:**
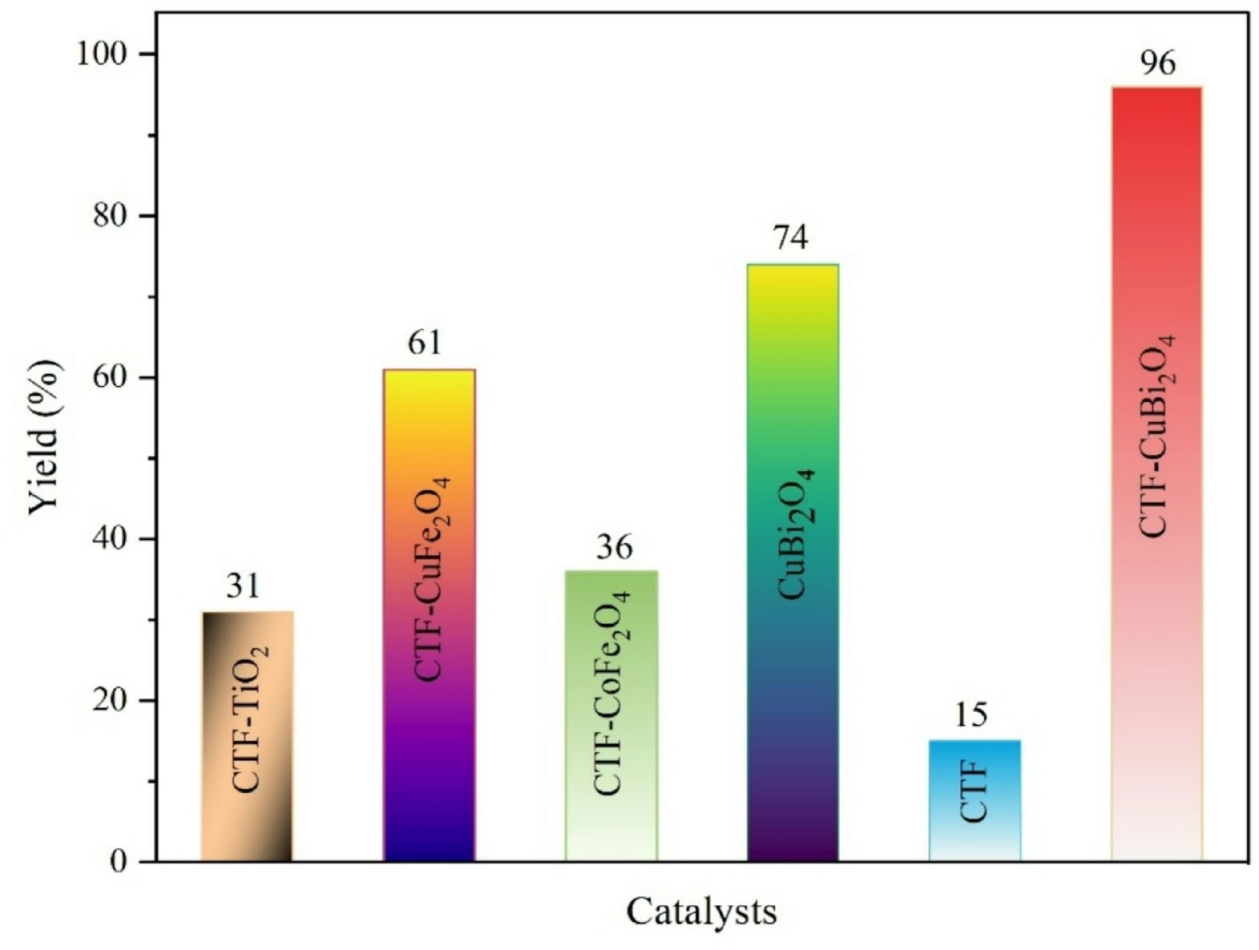
The catalyst’s effect on the reaction.

The influence of several solvents on product yield was analyzed (Table 3, entries 1–7). n-Hexane was identified as the most suitable solvent compared to others. Multiple oxidants, such as TBHP, H₂O₂ (30%), Oxone (Tetrahydropyran), and K₂S₂O₈, were employed (Table 3, entries 8–10). Among the oxidants tested, TBHP gave the best results, achieving 96% conversion with high selectivity to benzaldehyde. This superior performance arises because TBHP readily generates reactive tBuO· and ·OH radicals under light irradiation (e.g., UV or visible light) in the presence of a catalyst, which efficiently promotes the oxidation of benzyl alcohol. In some reactions, TBHP exhibits better selectivity than other oxidants, such as H₂O₂ or O₂, oxidizing only specific groups, which leads to higher yields and purer products. This is because one oxidant often responds better to the photocatalyst under light conditions than the others, depending on the conduction band levels of the photocatalyst and the oxidant species’ ability to be reduced under light conditions, which is consistent with previous reports on the visible-light-driven oxidation of benzyl alcohol^65^. On the other hand, we could not obtain a substantial quantity of product without the oxidants (Table 3, entry 11), indicating that in our system (noble-metal-free), the presence of an oxidant (TBHP) is essential for achieving high efficiency and selectivity, even if CTF or CuBi₂O₄ were to exhibit sufficient visible-light absorption. Additionally, the reaction was performed with varying amounts of the oxidant (Table 3, entries 12–14) and during different durations (Table 3, entries 15–19). The findings indicated that optimal outcomes were achieved with 3 mmol of the oxidant over 3 h. The results suggest that CTF and CuBi₂O₄ were unable to oxidize the reactant independently (Table 3, entries 20 and 21) because of insufficient visible light absorption, which hindered the generation of electron-hole pairs. The reaction was also examined with alternative photocatalysts (Table 3, entries 22–24), yielding 36%, 61%, and 31%, respectively, which were considerably inferior to the photocatalysts chosen in this work. The FT-IR spectra of CTF-CoFe_2_O_4_, CTF-CuFe_2_O_4_, and CTF-TiO_2_ are depicted in Fig. S3-S5. The effect of several catalysts on the process is illustrated in Fig. 12.

**Table 3:** Optimization of oxidation of benzyl alcohol^a^.


Entry	Photocatalyst	Solvent	Oxidant (mmol)	Time (h)	Yield (%)^b^
1	CTF-CuBi_2_O_4_	n-Hexane	TBHP (3)	3	96
2	CTF-CuBi_2_O_4_	THF	TBHP (3)	3	51
3	CTF-CuBi_2_O_4_	DMF	TBHP (3)	3	68
4	CTF-CuBi_2_O_4_	Ethanol	TBHP (3)	3	83
5	CTF-CuBi_2_O_4_	H_2_O	TBHP (3)	3	58
6	CTF-CuBi_2_O_4_	EtOAc	TBHP (3)	3	80
7	CTF-CuBi_2_O_4_	CH_3_CN	TBHP (3)	3	88
8	CTF-CuBi_2_O_4_	n-Hexane	H_2_O_2_ (30%) (3)	3	87
9	CTF-CuBi_2_O_4_	n-Hexane	Oxone (3)	3	62
10	CTF-CuBi_2_O_4_	n-Hexane	K_2_S_2_O_8_ (3)	3	62
11	CTF-CuBi_2_O_4_	n-Hexane	-	3	10
12	CTF-CuBi_2_O_4_	n-Hexane	TBHP (1)	3	82
13	CTF-CuBi_2_O_4_	n-Hexane	TBHP (2)	3	96
14	CTF-CuBi_2_O_4_	n-Hexane	TBHP (4)	3	96
15	CTF-CuBi_2_O_4_	n-Hexane	TBHP (3)	1	59
16	CTF-CuBi_2_O_4_	n-Hexane	TBHP (3)	2	78
17	CTF-CuBi_2_O_4_	n-Hexane	TBHP (3)	4	96
18	CTF-CuBi_2_O_4_	n-Hexane	TBHP (3)	5	96
19	CTF-CuBi_2_O_4_	n-Hexane	TBHP (3)	6	96
20	CTF	n-Hexane	TBHP (3)	3	15
21	CuBi_2_O_4_	n-Hexane	TBHP (3)	3	74
22	CTF-CoFe_2_O_4_	n-Hexane	TBHP (3)	3	36
23	CTF-CuFe_2_O_4_	n-Hexane	TBHP (3)	3	61
24	CTF-TiO_2_	n-Hexane	TBHP (3)	3	31

^a^ Reaction conditions: 4-methoxybenzyl alcohol (0.138 g, 1 mmol), photocatalyst (30 mg), oxidant (3 mmol), solvent (3 mL), room temperature, 20 W blue LED, 3 h. ^b^Isolated yield.

To quantitatively assess the intrinsic catalytic activity of the CTF–CuBi₂O₄ photocatalyst, turnover number (TON) and turnover frequency (TOF) were calculated. Using a conservative normalization based on the total Cu and Bi metal content (30 mg catalyst, 25.23 wt% Cu, 52.69 wt% Bi), the TON was 4.93 and the TOF was 1.64 h⁻¹ for the oxidation of 4-methoxybenzyl alcohol (1.0 mmol, 96% conversion in 3 h). The corresponding mass-specific productivity was 10.67 mmol g⁻¹ h⁻¹, and the surface-area-normalized rate was ≈ 320 µmol m⁻² h⁻¹ (BET 33.37 m² g⁻¹). In addition, assuming 1–5 surface-active sites per nm², the BET-based site-density approach yields TON values in the range of 116–578 and TOF values of 38.5–193 h⁻¹. These results are consistent with literature practices for noble-metal-free heterogeneous photocatalysts. All data are summarized and shown in Table S1^66,67^.

We oxidized several benzyl alcohols under the standard conditions outlined in Table 4. All items had an exceptional yield. Various functional groups were examined under optimal reaction circumstances and compared to electron-donating and electron-withdrawing groups, including Cl, Br, Me, NO₂, OH, OMe, isopropyl, and NMe₂. Electron-donating groups are more reactive than electron-withdrawing groups, yielding higher reactions. Table 4 shows that benzyl alcohols with *O*-substitution yielded lower results than their counterparts with *P*-substitution because of the strict hindrance effect at the *ortho*-position. In addition, in electron-withdrawing groups, *m*-substitution shows higher yields than *O*-substitution and *P*-substitution, unlike the electron-donating groups.

**Table 4:** Synthesized different benzaldehyde derivatives at the optimum oxidation reaction condition^a^.


2a-q, Yield (%), TON, TOF (h^− 1^)			
			
			
			
			
	

^a^Reaction conditions: substrate (1 mmol), CTF-CuBi_2_O_4_ (30 mg), TBHP (3 mmol), *n*-hexane (3 mL), room temperature, 20 W blue LED, 3 h.

When comparing our approach to those employed in previous research, we identified several drawbacks in the existing methods, including extended reaction times and the need for costly and difficult-to-obtain raw materials (as shown in Table 5). In contrast, our work offers clear benefits by significantly reducing the overall reaction time, utilizing inexpensive materials, and avoiding noble metals such as Pd, which are costly and prone to leaching throughout the reaction.

**Table 5:** Comparison of various types of benzaldehyde synthesis.

Entry	Catalyst	Reaction Condition	Yield (%)	Reference
**1**	40-CBO/WO_3_	1 mmol benzyl alcohol, 1.6 mmol of CsCO_3_, 6 mL n-hexane, air atmosphere, 500 W halogen lamp, 30 ± 3 ◦C, 14 h.	54.8 (conv.), > 99 sel.	^49^
**2**	3 wt% Pd@CBO	1 mmol benzyl alcohol, 1 mmol NaOH, 6 mL DMF, air atmosphere, 500 W halogen lamp, 30 ± 3 ◦C, 14 h.	73.2 (conv.), > 99 sel.	^68^
**3**	4% Au/CuBi_2_O_4_	1 mmol benzyl alcohol, 0.8 mmol of Cs_2_CO_3_, 6 mL n-heptane, air atmosphere, room temperature, visible light, 30 ± 3 ◦C, 14 h.	88.9 (conv.), > 99 sel.	^69^
**4**	4% CBO/HP-TiO_2_ hetero-system	4-methoxybenzyl alcohol, water, simulated solar light irradiation, 4 h	35	^70^
**5**	CTF-CuBi_2_O_4_	1 mmol benzyl alcohol, TBHP (3 mmol), n-hexane (3 mL), room temperature, 20 W blue LED, 3 h.	96	This work

## Photocatalytic mechanism

**Fig. 13:**
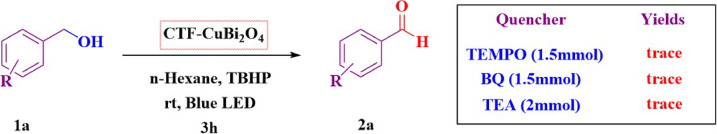
Control experiments.

This work utilized 2,2,6,6-tetramethylpiperidine-1-oxyl (TEMPO), 1,4-benzoquinone (BQ), and triethanolamine (TEA) as electron trapping agents in the conduction band (CB) and for photo-generated holes (h⁺), respectively. The introduction of BQ into the photocatalytic system markedly lowered the yield of benzyl alcohol oxidation, suggesting that the photo-generated electrons were effectively trapped and represent the key reactive species driving the transformation. When TEMPO was employed as a trapping agent, the efficiency of benzaldehyde formation declined substantially compared to experiments conducted without any trapping agent, further confirming the participation of radical intermediates. Similarly, the addition of TEA diminished photocatalytic performance by quenching photogenerated holes (h⁺), thereby impeding electron–hole separation and reducing the generation of oxidative species. Taken together, these observations demonstrate that the photocatalytic oxidation of benzyl alcohol over the CTF–CuBi₂O₄ hybrid catalyst proceeds predominantly through a radical-mediated mechanism (see Fig. 13).

**Fig. 14:**
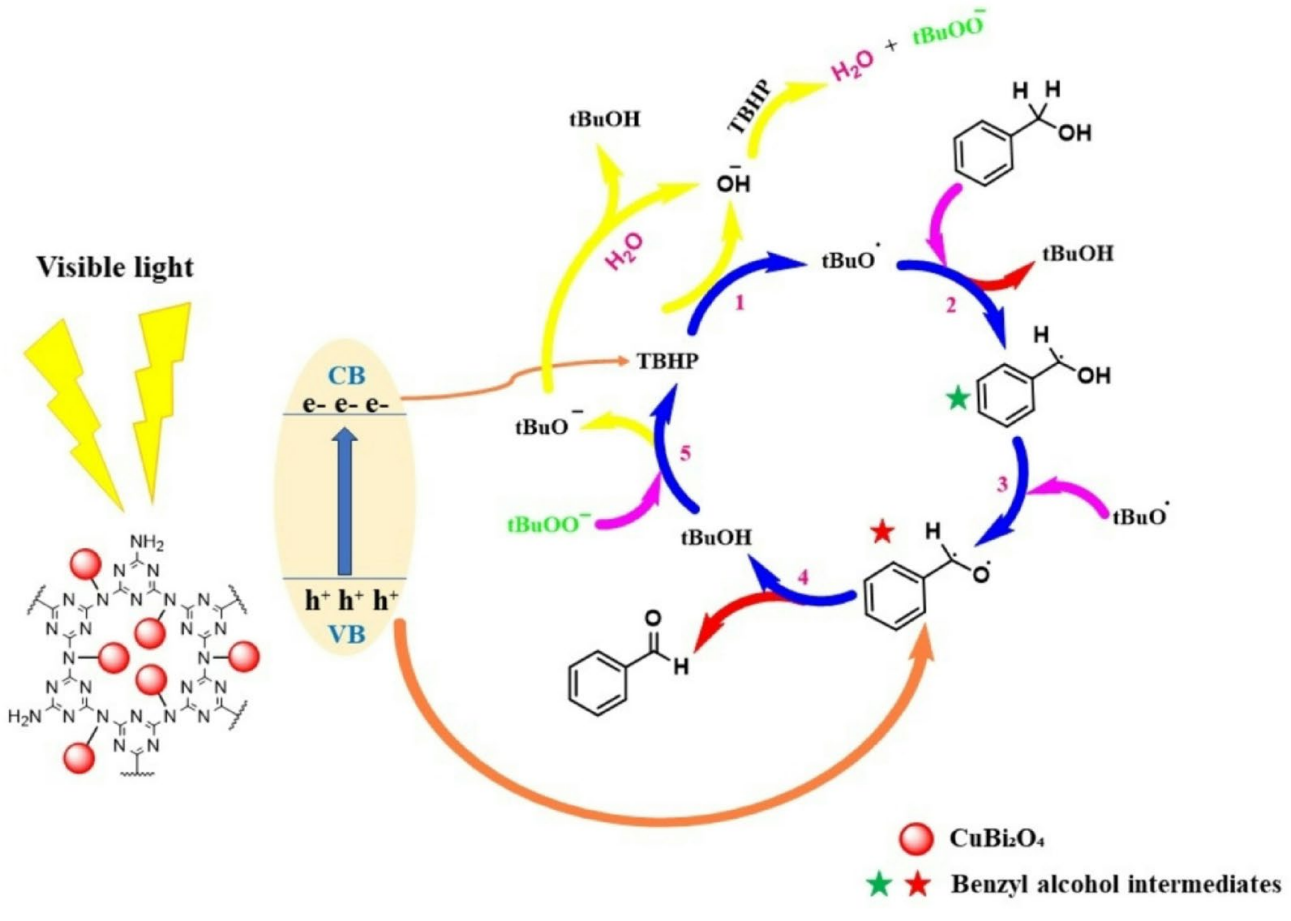
Proposed mechanism for the synthesis of Benzaldehydes.

Figure 14 illustrates the photocatalytic mechanism of the oxidation reaction. Under visible light irradiation, the CTF-CuBi₂O₄ composite acts as the photocatalyst, so electrons (e⁻) are excited from the valence band (VB) to the conduction band (CB) by leaving behind photogenerated holes (h⁺). After that, these charge carriers initiate redox reactions in the system, and the photogenerated electrons (e⁻) participate in reducing TBHP, leading to the generation of tBuO^•^ and OH⁻. The tBuO^•^ drives oxidation reactions^71–74^. The tBuO^•^ abstracts a hydrogen atom from benzyl alcohol, forming a benzyl radical intermediate (marked with a green star in the diagram). This intermediate undergoes further oxidation by tBuO^•^ radicals. It abstracts a hydrogen atom from the benzyl radical intermediate’s oxygen, forming another benzyl radical intermediate (marked with a red star in the diagram). Finally, it leads to the formation of benzaldehyde as the final oxidation product. In addition, the generated radicals propagate the reaction cycle, maintaining continuous oxidation; also, the CTF-CuBi₂O₄ composite photocatalyst remains active under visible light and facilitates sustained oxidation. The current procedure achieved an ultra-high yield of benzaldehyde due to the combination of n-type CTF and p-type semiconductor CuBi₂O₄, which gives p-n junction, generating a substantial quantity of h⁺, which facilitated the oxidation of the benzyl alcohol intermediate to benzaldehyde.

## Recycling test of the photocatalyst

The stability of CTF-CuBi₂O₄ was assessed for recyclability in the oxidation reaction under optimal circumstances, including green chemistry and industrial applications. To regenerate the catalyst, it was separated by centrifugation after each cycle, washed with ethanol and water to eliminate organic components, dried in an oven, and utilized in the following run. The catalyst can be recycled and reused six times with minimal loss in product yield (Fig. S6a). This finding indicates that the leaching of CTF-CuBi₂O₄ nanoparticles was minimal. XRD and SEM investigations were conducted on the recycled photocatalyst following six cycles (Fig. S6b). The results indicate that the morphology of the catalyst remained unchanged, confirming that the photocatalyst is a reusable and stable heterogeneous catalyst.

## Hot filtration test

**Fig. 15:**
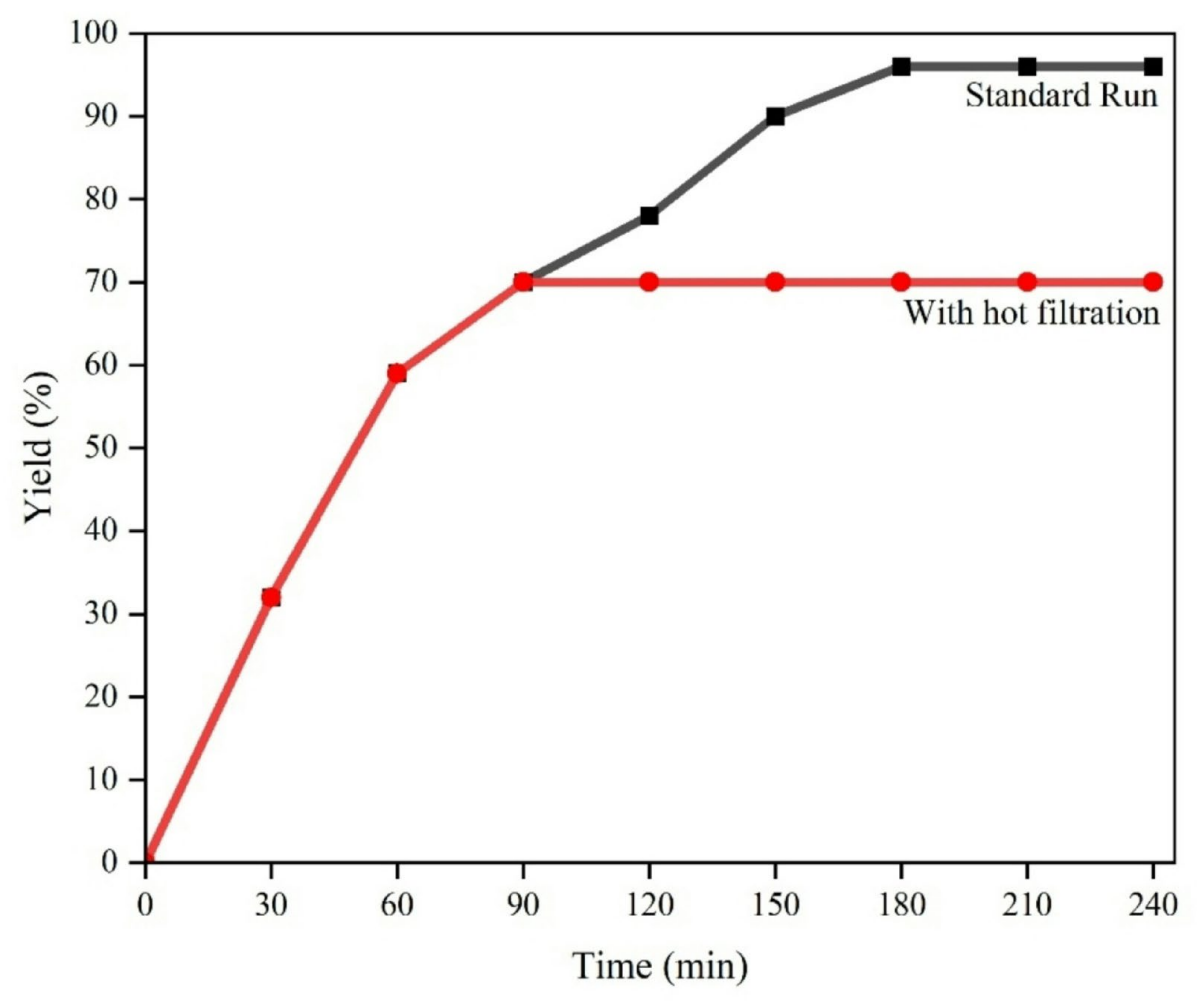
The time course of the oxidation reaction using CTF-CuBi₂O₄ photocatalysis (standard run) and after hot filtration of the photocatalysis.

To confirm the heterogeneous nature of the photocatalyst in the model reaction, a hot filtration test was conducted (Fig. 15). The reaction mixture was prepared within a test tube and stirred under an LED. The reaction was paused after 1.5 h, and the reaction mixture was cooled to room temperature; then, the catalyst was separated by centrifuge, so the experiment was continued with the filtrate for 4 h. After removal of the photocatalyst, no progress in the reaction was observed. The result showed no significant leaching, indicating the high stability and heterogeneous nature of the photocatalyst under the reaction conditions.

## Conclusion

A novel composite of the covalent triazine-based organic framework (CTF) combined with CuBi_2_O_4_ nanoparticles was successfully generated. It was used as an efficient photocatalyst activated by visible light to oxidize benzyl alcohols to corresponding benzaldehydes in the presence of TBHP after discovery and appraisal using various spectroscopic investigations. TEM and SEM studies turned up nanoparticles with a 103 nm range. According to UV-DRS and PL experiments, the photocatalyst demonstrates notable visible light absorption and, conversely, negligible emission (showing a low electron-hole pair recombination rate), demonstrating its better efficacy in oxidation reactions within the visible light spectrum. After six recycling runs, the photocatalyst shows remarkable performance, free from any morphological change.

## Supplementary Information

Below is the link to the electronic supplementary material.

Supplementary Material 1

## Data Availability

The data that support the findings of this study are available in the supplementary material of this article.
