# Supplementary material for: Covalent organic frameworks-supported copper bismuth oxide nanoparticles as an efficient and green photocatalyst for benzyl alcohol oxidation
Source: Sci Rep. 2025 Nov 21;15:41348. doi: 10.1038/s41598-025-25368-8 (PMC12638315; doi:10.1038/s41598-025-25368-8)
Supplement: Supplementary file 1 — Supplementary Material 1 [file 41598_2025_25368_MOESM1_ESM.docx]

**Supporting Information**

**Covalent Organic Frameworks-Supported Copper Bismuth Oxide Nanoparticles as an Efficient and Green Photocatalyst for Benzyl Alcohol Oxidation**

**Sanaz HajimohamadzadehTorkambour^a^, Masoumeh Jadidi Nejad*^b^, Farzane Pazoki^a^, and Akbar Heydari*^a^**

*^a^Chemistry Department, Tarbiat Modares University, P.O. Box 14155-4838, Tehran, Iran. E-mail: [heydar_a@modares.ac.ir](file:///C:\\Users\\TechCenter\\AppData\\Roaming\\Microsoft\\Word\\heydar_a@modares.ac.ir); Fax: +98-21-82883455; Tel: +98-21-82883444*

*^b^Department of Chemistry, Isfahan University of Technology, P.O. Box 84156-83111, Isfahan, Iran.*

*E-mail:* [*m.jadidinejad@iut.ac.ir*](mailto:m.jadidinejad@iut.ac.ir)*; Tel: +98-31-33913261*

**Table of contents**

| 1. **Experimental ………………………………………………………………** | **S3** |
| --- | --- |
| 1. **Characterization of the catalyst ………………………………………….** | **S5** |
| **Fig. S1.** The Mott-Schottky plot of CTF……………………………………………………. | **S5** |
| **Fig. S2.** The band energy diagrams of CTF and CoFe_2_O_4_ before contact (a) and after contact (b)…………………………………………………………………………………………… | **S5** |
| **Fig. S3.** FT-IR spectrum of CTF-CoFe_2_O_4_…………………………………………………. | **S6** |
| **Fig. S4.** FT-IR spectrum of CTF-CuFe_2_O_4_…………………………………………………. | **S6** |
| **Fig. S5.** FT-IR spectrum of CTF-TiO_2_……………………………………………………. | **S7** |
| **Fig. S6.** Recycling test of the CTF-CuBi₂O₄ photocatalyst in Oxidation reactions (a). XRD pattern and FE-SEM image of the recovered CTF-CuBi₂O₄ photocatalyst (b)…………….. | **S8** |
| **Table S1.** TON and TOF of CTF–CuBi₂O₄ under optimized conditions…………………. | **S9** |
| 1. **Characterization of the corresponding benzaldehyde products ……….** | **S10** |
| 1. **^1^H NMR and ^13^C NMR spectra of benzaldehyde derivatives …………...** | **S15** |
| 1. **FT-IR spectrum of benzaldehyde derivatives …………………………...** | **S32** |
| 1. **References ………………………………………………………………….** | **S42** |

**EXPERIMENTAL**

**Materials and methods**

All the materials were purchased from Merck (Germany) and Aldrich (China). Thin-layer chromatography was used to monitor the reaction progress. TLC was conducted on glass plates using silica-gel 60 F-254 as the matrix. Infrared (IR) spectra within the range of 400-4000 cm⁻¹ were recorded using KBr pellets on a Nicolet IR100 instrument. X-ray diffraction (XRD) analysis was performed at room temperature utilizing a Philips X-pert 1710 equipped with monochromated Cu Kα radiation, covering a 2θ range of 10° to 80°. A TESCAN MIRA III Field Emission Scanning Electron Microscope (FE-SEM) was used to examine the morphology and size of the particles. EDAX analysis was carried out to determine the elemental composition of the catalysts. Thermal gravimetric analysis (TGA) was conducted with a thermal analyzer, operating at a heating rate of 20 °C min⁻¹ over a temperature range of 25-800 °C in an air environment. Transmission electron microscopy (TEM) imaging was performed using a Philips CM 120 at an accelerating voltage of 120 kV. The Brunauer-Emmett-Teller (BET) method was employed to determine the nanocatalyst’s surface area through nitrogen gas physisorption, using the BELSORP MINI II instrument at 77 K. UV-visible diffuse reflectance spectroscopy (DRS) measurements were conducted using a Shimadzu UV-2550 spectrophotometer within the 200-800 nm wavelength range. For the Matt-Schottky experiment, a Na₂SO₄ (0.5 M) electrolyte solution and a calomel reference electrode were used. Additionally, a Shimadzu RF6000 fluorescence spectrophotometer was utilized to analyze the photoluminescence (PL) spectra. The ¹H NMR and ¹³C NMR spectra were obtained using a Bruker DRX-300 Avance spectrometer at 300 and 75 MHz, respectively.

**Synthesis of CTF**

Firstly, 0.66 gr of melamine (5.2 mmol), 0.5 gr of triethylamine (5 mmol), and 10 mL of DMF were mixed by ultrasonication within a round-bottom flask for 10 minutes (solution 1); then, 0.92 gr of trichlorotriazine (TCT) (5mmol) was added to solution 1 and was sonicated for 30-40 minutes. Next, the mixture was conveyed into a Teflon-lined autoclave for 24 h at 120 ^0^C. Then, the solution was cooled to room temperature, and a white precipitate was separated by centrifuging and eluted with dimethylformamide and ethanol several times. Finally, it was dried in the oven at 80 ^0^C to acquire CTF.

**Synthesis of CTF-CuBi_2_O_4_**

0.44 gr of copper (II) nitrate hexahydrate (1.5 mmol) was solved in 10 mL of deionized (DI) water within a round-bottom flask by ultrasonic for 10 minutes (solution 1); then, 1.4 gr of bismuth (III) nitrate pentahydrate (3 mmol) was solved in 10 mL of DI water by ultrasonic for 10 minutes (solution 2). Afterward, solution 1 and solution 2 were sonicated for 30 minutes. After that, 0.6 gr of CTF was added to the previous mixture, and the homogenous solution was mixed by ultrasonication for 30 minutes. A solution of sodium hydroxide (0.2 M) (20 mL, 0.16 gr) was added to adjust pH=12. Then, the mixture was placed in an autoclave for 24 h at 180 ^0^C. In the next step, the black solid product was separated by centrifuge and washed with DI water and ethanol several times. Finally, the product was dried in the oven at 80 ^0^C to acquire CTF-CuBi_2_O_4_.

**Synthesis of Benzaldehyde**

The tests were conducted under visible light illumination using a 20 W blue LED lamp as the light source. A white LED with 400-750 nm wavelength was used, while a 460 nm wavelength was applied for the blue LED. The light intensity was 1.6 × 10⁻² W·cm⁻². A reaction mixture containing 1 mmol of benzyl alcohol, 3 mmol of TBHP (tert-butyl hydroperoxide, 70% aqueous solution), and 30 mg of catalyst was prepared in 3 mL of n-hexane within a test tube. This mixture was stirred for 3 h under LED. The progress of the reaction was evaluated by thin-layer chromatography. After completion of the reaction, the solution was cooled to room temperature; then, the catalyst was separated by centrifuge and extracted with ethyl acetate. Finally, the product had to be isolated by column chromatography (ethyl acetate and hexane in a ratio of 3:2) on silica gel. Also, it was mentioned that the characterizations of the isolated products were based on using ^13^C NMR, ^1^H NMR, and Mass spectroscopy.

**Characterization of the catalyst:**

| 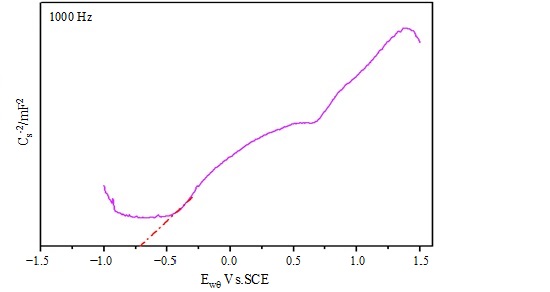 |
| --- |
| **Fig. S1** The Mott-Schottky plot of CTF |

| **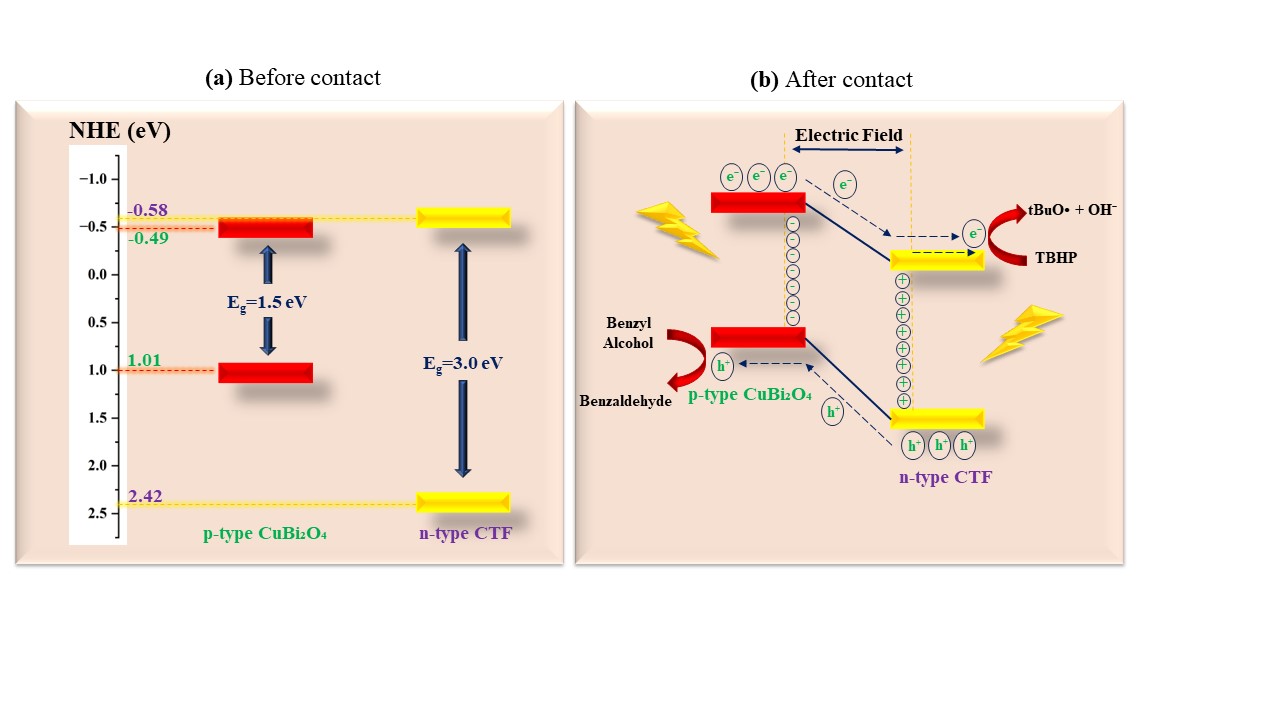** |
| --- |
| **Fig. S2** The band energy diagrams of CTF and CoFe_2_O_4_ before contact (a) and after contact (b) |

|  |
| --- |
| **Fig. S3** FT-IR spectrum of CTF-CoFe_2_O_4_ |

|  |
| --- |
| **Fig. S4** FT-IR spectrum of CTF-CuFe_2_O_4_ |

|  |
| --- |
| **Fig. S5** FT-IR spectrum of CTF-TiO_2_ |

| 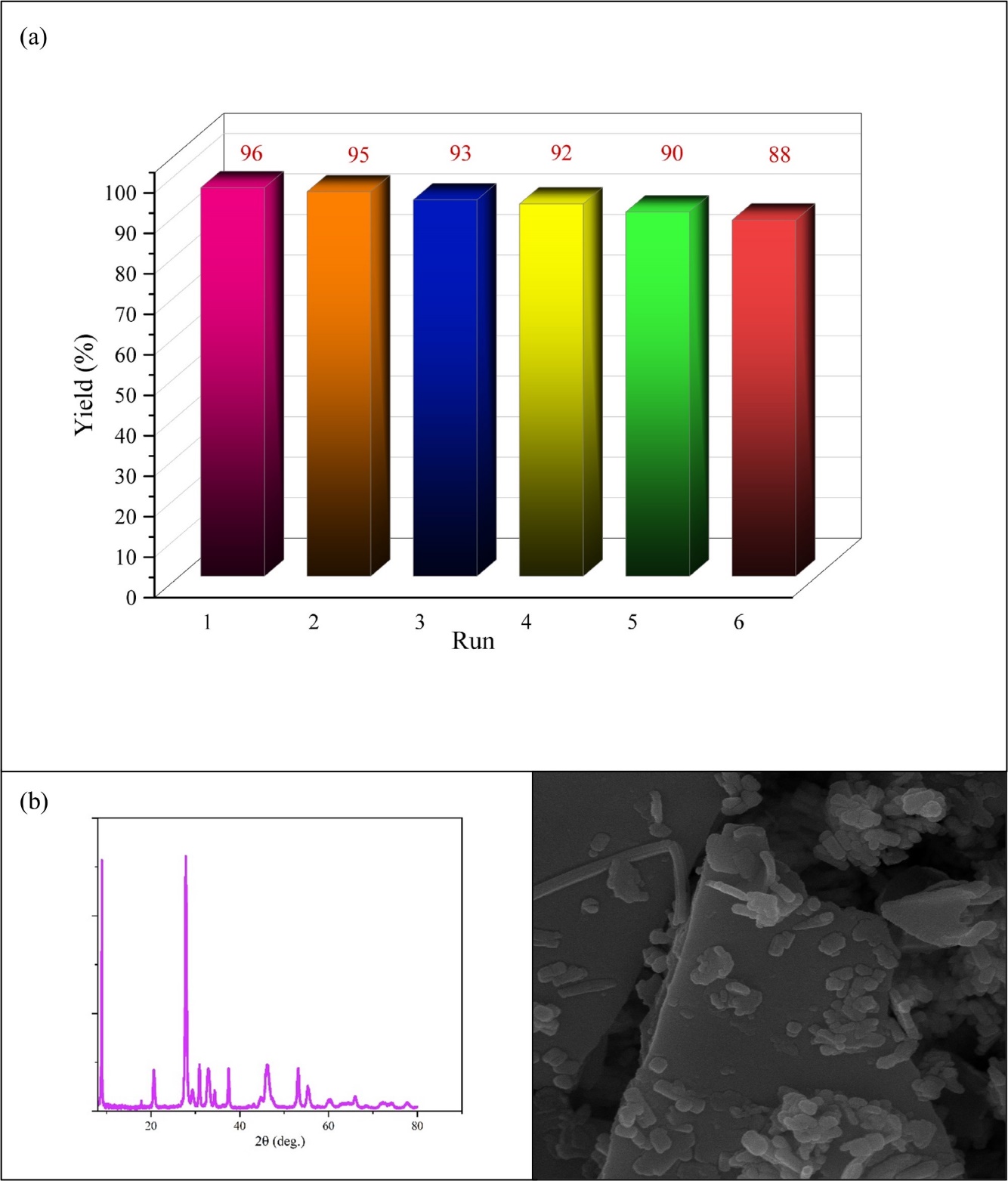 |
| --- |
| **Fig. S6** Recycling test of the CTF-CuBi₂O₄ photocatalyst in Oxidation reactions (a). XRD pattern and FE-SEM image of the recovered CTF-CuBi₂O₄ photocatalyst (b). |

**Table S1. TON and TOF of CTF–CuBi₂O₄ under optimized conditions^a^.**

| **Normalization method** | **Basis of calculation** | **TON** | **TOF (h⁻¹)** |
| --- | --- | --- | --- |
| **Per total metal atoms (Cu + Bi)** | 25.23 wt% Cu + 52.69 wt% Bi in 30 mg catalyst | 4.93 | 1.64 |
| **Per catalyst mass** | mmol product per g catalyst per h | - | 10.67 mmol g⁻¹ h⁻¹ |
| **Per BET surface area** | 33.37 m² g⁻¹ → 1.001 m² (30 mg catalyst) | - | 320 μmol m⁻² h⁻¹ |
| **BET site density: 1 site nm⁻²** | Active sites estimated from surface area | 578 | 193 |
| **BET site density: 5 sites nm⁻²** | Active sites estimated from surface area | 116 | 38.5 |
| ^a^Optimized conditions: 1 mmol 4-methoxybenzyl alcohol, 30 mg catalyst, 96% conversion, 3 h. | | | |

**Characterization of the corresponding benzaldehyde products:**

**Benzaldehyde (2a):** Colorless liquid; 93% isolated yield; ^1^H NMR (500 MHz, CDCl3) δ 9.76 (s, 1H), 7.62 (dd, J_1_ = 8.4, J_2_ = 1.5 Hz, 2H), 7.36 – 7.33 (m, 1H), 7.24 (t, J = 7.6 2H); FT-IR (KBr) ῡ 3065, 2819, 2736, 1696, 1594, 1202, 1166, 826, 744, 686, 647 cm^-1^; ^13^C NMR (126 MHz, CDCl3) δ 192.23, 136.52, 134.38, 129.64, 129.00 ppm; MS (EI, 70eV): m/z: 106.5 [M]^+^.

**4-Methoxybenzaldehyde (2b):** Colorless oil; 96% isolated yield; ^1^H NMR (500 MHz, CDCl3) δ 9.83 (s, 1H), 7.78 (d, J = 8.4 Hz, 2H), 6.95 (d, J = 8.4 Hz, 2H), 3.83 (s, 3H); FT-IR (KBr) ῡ 2937, 2839, 2739, 1682, 1596, 1509, 1215, 1156, 1022, 829 cm^-1^; ^13^C NMR (126 MHz, CDCl3) δ 190.89, 164.78, 132.08, 130.12, 114.47, 55.69 ppm; MS (EI, 70eV): m/z: 136 [M]^+^.

**4-Chlorobenzaldehyde (2c):** White solid; 93% isolated yield; Mp: 46-48 °C; ^1^H NMR (500 MHz, CDCl3) δ 9.97 (s, 1H), 7.80 (d, J = 8.1 Hz, 2H), 7.49 (d, J = 8.1 Hz, 2H); FT-IR (KBr) ῡ 3047, 2858, 2759, 1684, 1578, 1477, 1417, 1286, 1202, 829, 807, 467 cm^-1^; ^13^C NMR (126 MHz, CDCl3) δ 190.94, 141.08, 134.95, 131.04, 129.59 ppm; MS (EI, 70eV): m/z: 140 [M]^+^.

**4-Hydroxybenzaldehyde (2d):** Light yellow to light brown powder; 96% isolated yield; Mp: 112-116 °C; ^1^H NMR (500 MHz, CDCl3) δ 10.57 (s, 1H), 9.77 (s, 1H), 7.74 (d, J = 8.5 Hz, 2H), 6.92 (d, J = 8.5 Hz, 2H); FT-IR (KBr) ῡ 3168, 2878, 1906, 1668, 1599, 1518, 1453, 1314, 1287, 1217, 1160, 1113, 859, 789, 697, 640 cm^-1^; ^13^C NMR (126 MHz, CDCl3) δ 191.16, 162.74, 132.56, 128.53, 116.00 ppm; MS (EI, 70eV): m/z: 122.2 [M]^+^.

**4-(Dimethylamino)benzaldehyde (2e):** Yellow-white powder; 86% isolated yield; Mp: 72-75 °C; ^1^H NMR (500 MHz, CDCl3) δ 9.71 (s, 1H), 7.71 (d, J = 8.8, 2H), 6.67 (d, J = 8.8, 2H), 3.05 (s, 6H); FT-IR (KBr) ῡ 2981, 2362, 1699, 1056, 1053, 1031, 960 cm^-1^; ^13^C NMR (126 MHz, CDCl3) δ 190.14, 154.41, 131.67, 125.18, 110.63, 40.12 ppm; MS (EI, 70eV): m/z: 149 [M]^+^.

**4-Methylbenzaldehyde (2f):** Colorless liquid; 92% isolated yield; ^1^H NMR (500 MHz, CDCl3) δ 9.94 (s, 1H), 7.78 (d, J = 8.0, 2H), 7.37 (d, J = 7.8, 2H), 2.37 (s, 3H); FT-IR (KBr) ῡ 3027, 2970, 2822, 2732, 1701, 1603, 1365, 1207, 1167, 846, 806, 757 cm^-1^; ^13^C NMR (126 MHz, CDCl3) δ 192.15, 145.83, 134.54, 130.16, 129.40, 22.01 ppm; MS (EI, 70eV): m/z: 120 [M]^+^.

**4-Isopropylbenzaldehyde (2g):** Colorless oil; 85% isolated yield; ^1^H NMR (500 MHz, CDCl3) δ 9.96 (s, 1H), 7.80 (d, J = 8.1 Hz, 2H), 7.37 (d, J = 7.9 Hz, 2H), 2.92 – 3.04 (m, 1H), 1.27 (d, J = 7.0 Hz, 6H); FT-IR (KBr) ῡ 3023, 2963, 2822, 2725, 1698, 1605, 1461, 1340, 1212, 1169, 1054, 829, 725 cm^-1^; ^13^C NMR (126 MHz, CDCl3) δ 192.15, 156.38, 134.75, 130.16, 127.29, 34.63, 23.77 ppm; MS (EI, 70eV): m/z: 148.1 [M]^+^.

**2-Hydroxybenzaldehyde (2h):** Colorless liquid; 94% isolated yield; ^1^H NMR (500 MHz, CDCl3) δ 11.01 (s, 1H), 9.86 (s, 1H), 7.44 – 7.59 (m, 2H), 6.89 – 7.06 (m, 2H); FT-IR (KBr) ῡ 3116, 3066, 2848, 2753, 1659, 1619, 1485, 1458, 1273, 1193, 1149, 881, 755, 706, 662 cm^-1^; ^13^C NMR (126 MHz, CDCl3) δ 196.75, 161.77, 137.11, 133.89, 120.85, 120.00, 117.74 ppm; MS (EI, 70eV): m/z: 122 [M]^+^.

**2-Chlorobenzaldehyde (2i):** Colorless liquid; 91% isolated yield; ^1^H NMR (500 MHz, CDCl3) δ 10.42 (s, 1H), 7.45 – 7.49 (m, 1H), 7.39 (dd, J_1_ = 8.1 Hz, J_2_ =1.3 Hz, 1H), 7.33 (t, J = 7.5 Hz, 1H); FT-IR (KBr) ῡ 3077, 2868, 1749, 1694, 1567, 1343, 1266, 1196, 1051, 824, 753, 714, 631 cm^-1^; ^13^C NMR (126 MHz, CDCl3) δ 189.84, 138.02, 135.25, 132.62, 130.73, 129.51, 127.43 ppm; MS (EI, 70eV): m/z: 140.5 [M]^+^.

**2-Bromobenzaldehyde (2j):** Colorless liquid; 91% isolated yield; ^1^H NMR (500 MHz, CDCl3) δ 10.33 (s, 1H), 7.91 – 7.85 (m, 1H), 7.65 – 7.62 (m, 1H), 7.51 – 7.39 (m, 2H); FT-IR (KBr) ῡ 2868, 2633, 1693, 1586, 1567, 1263, 1160, 822, 741, 686, 654, 526 cm^-1^; ^13^C NMR (126 MHz, CDCl3) δ 191.69, 135.79, 134.17, 133.71, 130.48, 127.89, 126.87 ppm; MS (EI, 70eV): m/z: 185.0 [M]^+^.

**3-Hydroxybenzaldehyde (2k):** White solid; 91% isolated yield; Mp: 100-102 °C; ^1^H NMR (500 MHz, CDCl3) δ 9.93 (s, 1H), 7.47 – 7.38 (m, 3H), 7.17 – 7.14 (m, 1H), 6.26 (s, 1H); FT-IR (KBr) ῡ 3212, 2878, 2561, 1668, 1581, 1284, 872, 782, 676, 430 cm^-1^; ^13^C NMR (126 MHz, CDCl3) δ 192.94, 156.89, 137.74,130.68, 123.51, 122.46, 114.79 ppm; MS (EI, 70eV): m/z: 122.1 [M]^+^.

**3-Bromobenzaldehyde (2l);** Yellow liquid; 85% isolated yield; ^1^H NMR (500 MHz, CDCl3) δ

9.95 (s, 1H), 8.00 (s, 1H), 7.80 (d, J = 7.6 Hz, 1H), 7.75 (d, J = 7.9 Hz, 1H), 7.42 (t, J = 7.8 Hz, 1H); FT-IR (KBr) ῡ 3067, 2827, 2727, 1693, 1569, 1468, 1428, 1382, 1188, 1063, 784, 671 cm^-1^; ^13^C NMR (126 MHz, CDCl3) δ 190.87, 138.19, 137.46, 132.52, 130.78, 128.52, 123.54 ppm; MS (EI, 70eV): m/z: 185.0 [M]^+^.

**3-Nitrobenzaldehyde (2m):** Yellowish to brownish powder; 79% isolated yield; Mp: 55-59 °C; ^1^H NMR (500 MHz, CDCl3) δ 10.12 (s, 1H), 8.67 – 8.65 (m, 1H),8.48 (dt, J_1_ = 8.1, J_2_ =4.0 Hz, 1H), 8.30 (d, J = 7.6 Hz, 1H), 7.87 (t, J = 7.9 Hz, 1H); FT-IR (KBr) ῡ 3097, 3066, 2877, 2768, 2346, 2283, 1701, 1611, 1580, 1528, 1395, 1274, 1101, 810, 727, 674, 666, 526 cm^-1^; ^13^C NMR (126 MHz, CDCl3) δ 189.85, 148.88, 137.37, 134.68, 130.68, 128.17, 124.13 ppm; MS (EI, 70eV): m/z: 151.1 [M]^+^.

**4-Nitrobenzaldehyde (2n):** White solid; 72% isolated yield; Mp: 103-106 °C; ^1^H NMR (500 MHz, CDCl3) δ 10.16 (s, 1H), 8.39 (d, J = 8.7 Hz, 2H), 8.07 (d, J = 8.9 Hz, 2H); FT-IR (KBr) ῡ 3107, 2850, 2733, 1704, 1603, 1534, 1342, 1194, 848, 814, 737, 680 cm^-1^; ^13^C NMR (126 MHz, CDCl3) δ 190.40, 151.32, 140.25, 130.63, 124.46 ppm; MS (EI, 70eV): m/z: 151.0 [M]^+^.

**3,4-Dimethoxybenzaldehyde (2o):** White solid; 93% isolated yield; Mp: 42-45 °C; ^1^H NMR (500 MHz, CDCl3) δ 9.84 (s, 1H), 7.44 (dd, J1 = 8.2 Hz, J2 =1.9 Hz, 1H), 7.39 (d, J = 1.9 Hz, 1H), 6.96 (d, J= 8.1 Hz, 1H), 3.95 (s, 3H), 3.92 (s, 3H); FT-IR (KBr) ῡ 3020, 2939, 2836, 2758, 1677, 1589, 1507, 1460, 1286, 1133, 1014, 726, 634 cm^-1^; ^13^C NMR (126 MHz, CDCl3) δ 190.97, 154.68, 149.83, 130.35, 126.93, 110.60, 109.21, 56.32, 56.16 ppm; MS (EI, 70eV): m/z: 166.1 [M]^+^.

**1-Naphthaldehyde (2p):** Colorless oil; 80% isolated yield; ^1^H NMR (500 MHz, CDCl3) δ 10.34 (s, 1H), 9.25 (d, J = 8.8 Hz, 1H), 7.99 (d, J = 8.6 Hz, 1H), 7.87 (dd, J1 = 7.0 Hz, J2 = 1.5 Hz, 1H), 7.84 (d, J = 8.2 Hz, 1H), 7.62 – 7.67 (m, 1H), 7.50 – 7.56 (m, 2H); FT-IR (KBr) ῡ 3051, 2839, 2725, 1648, 1622, 1458, 1216, 1168, 1054, 800, 647 cm^-1^; ^13^C NMR (126 MHz, CDCl3) δ 193.69, 136.69, 135.42, 133.88, 131.63, 130.65, 129.27, 128.72, 127.04, 125.08 ppm; MS (EI, 70eV): m/z: 156.1 [M]^+^.

**Furan-2-carbaldehyde (2q):** Colorless oil; 76% isolated yield; ^1^H NMR (500 MHz, CDCl3) δ 9.54 (s, 1H), 7.60 (s, 1H), 7.17 (s, 1H), 6.51 (s, 1H); FT-IR (KBr) ῡ 3133, 2850, 2813, 1669, 1466, 1391, 1276, 1017, 749 cm^-1^; ^13^C NMR (126 MHz, CDCl3) δ 177.96, 153.07, 148.25, 121.33, 112.72 ppm; MS (EI, 70eV): m/z: 96.1 [M]^+^.

**^1^H NMR and ^13^C NMR spectra of benzaldehyde derivatives:**

| **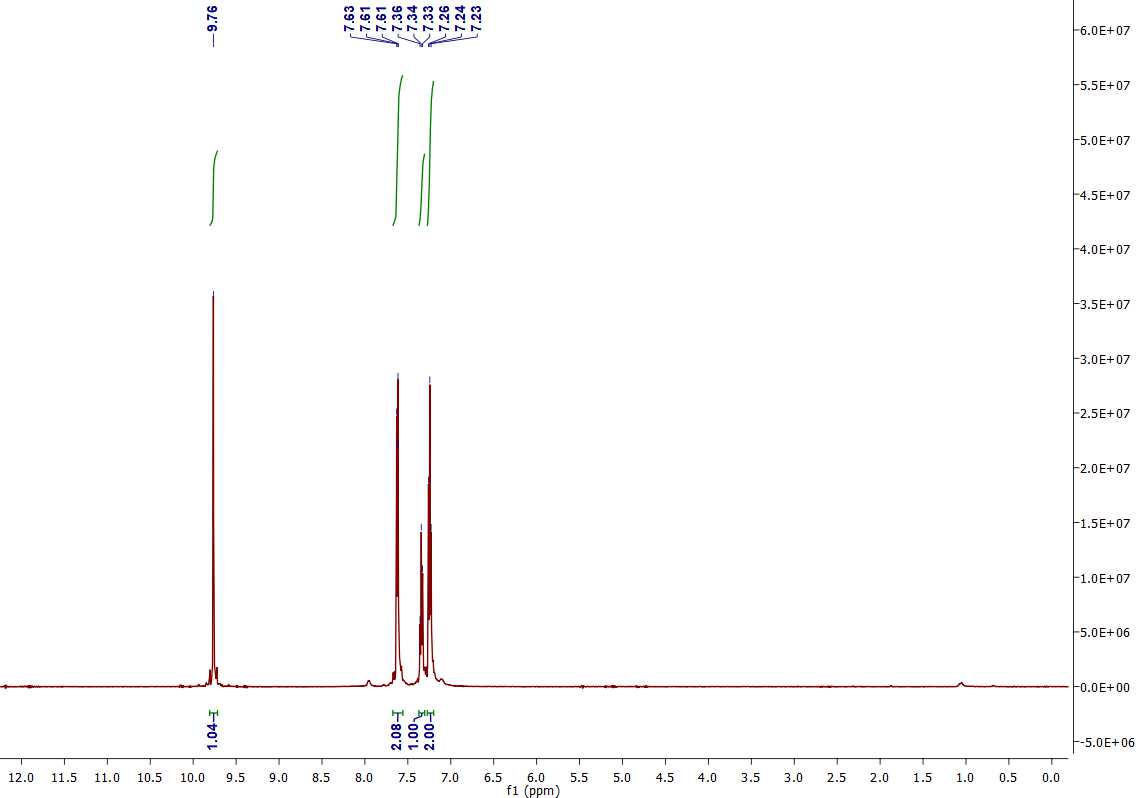**  **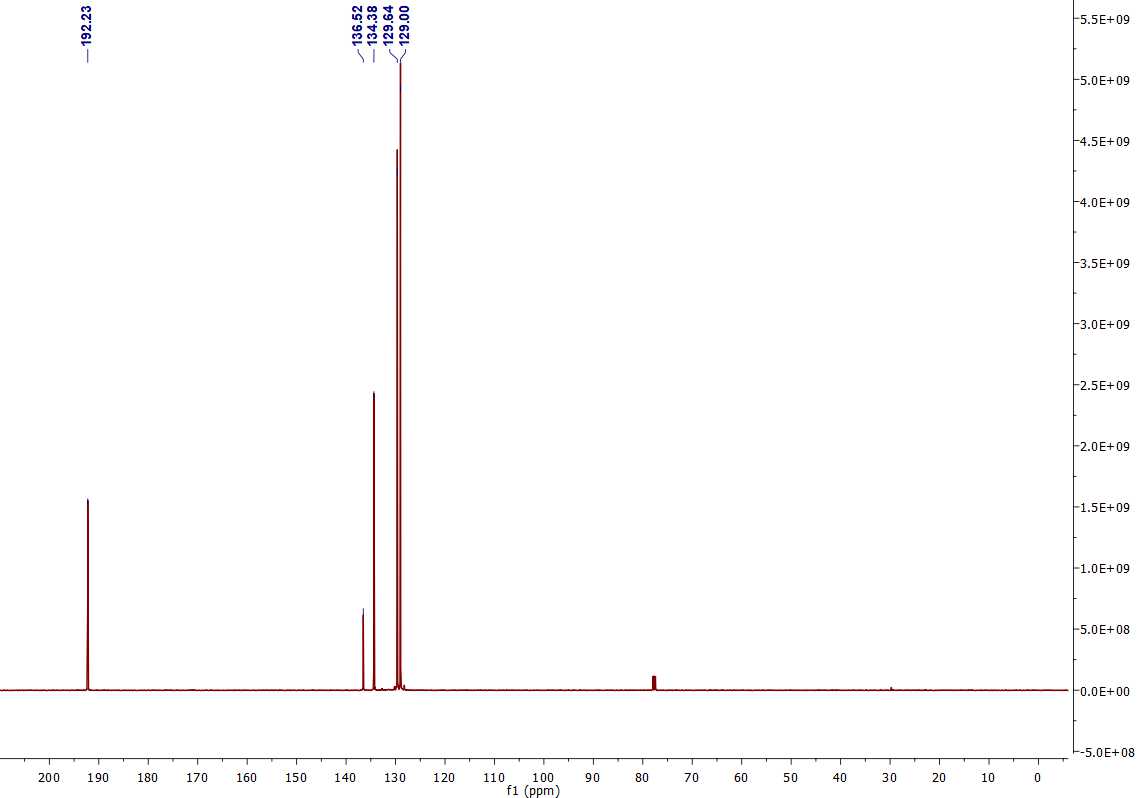**  **Fig. S7:** ^1^H NMR and ^13^C NMR of Benzaldehyde (2a) |
| --- |

| 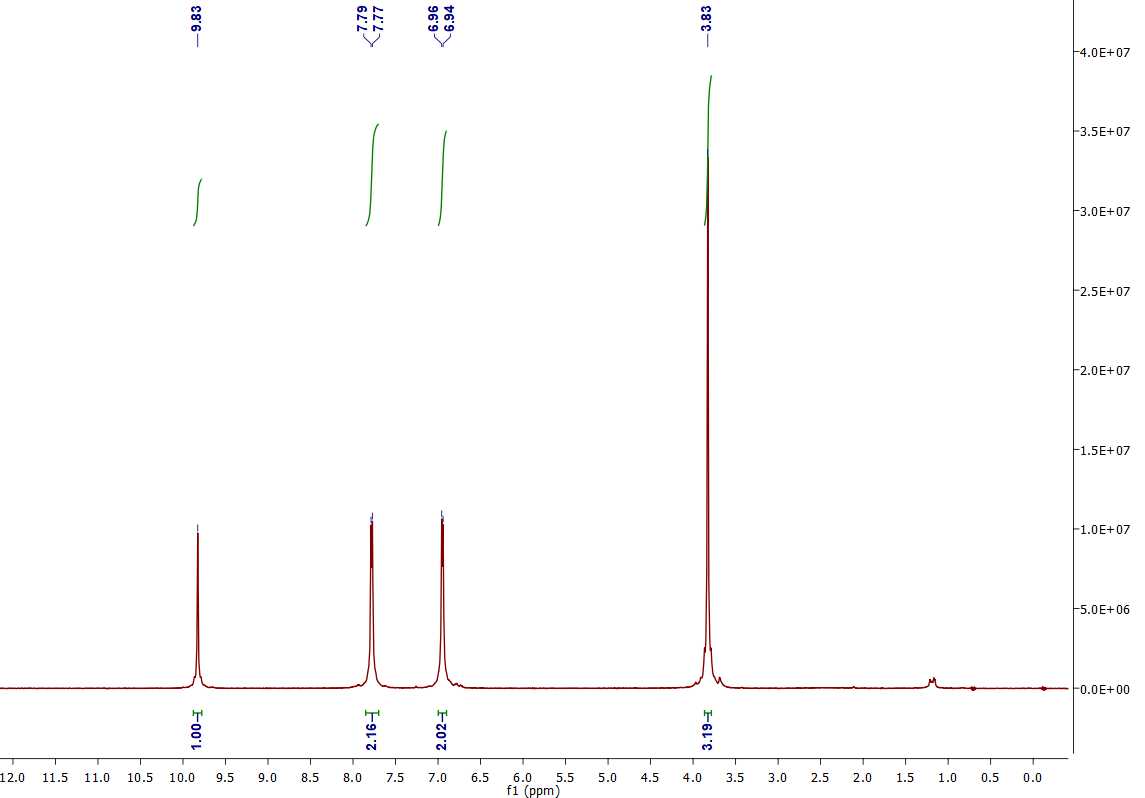  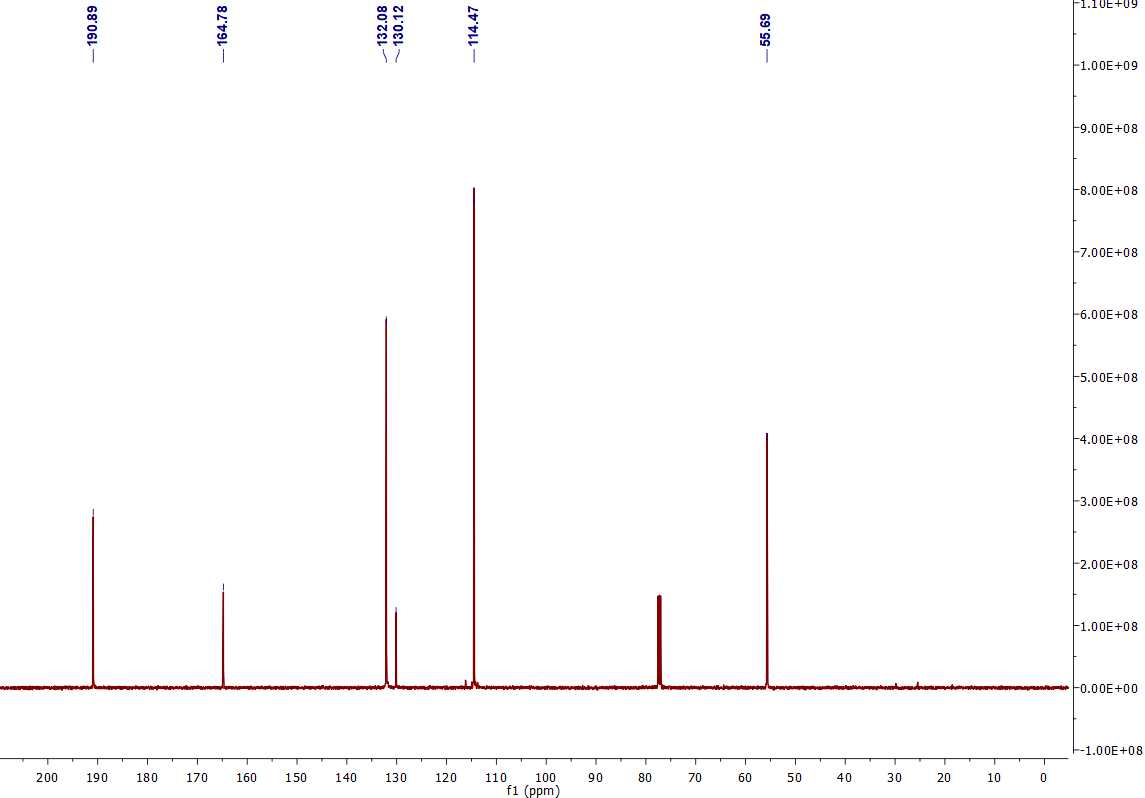  **Fig. S8:** ^1^H NMR and ^13^C NMR of 4-Methoxybenzaldehyde (2b) |
| --- |

| 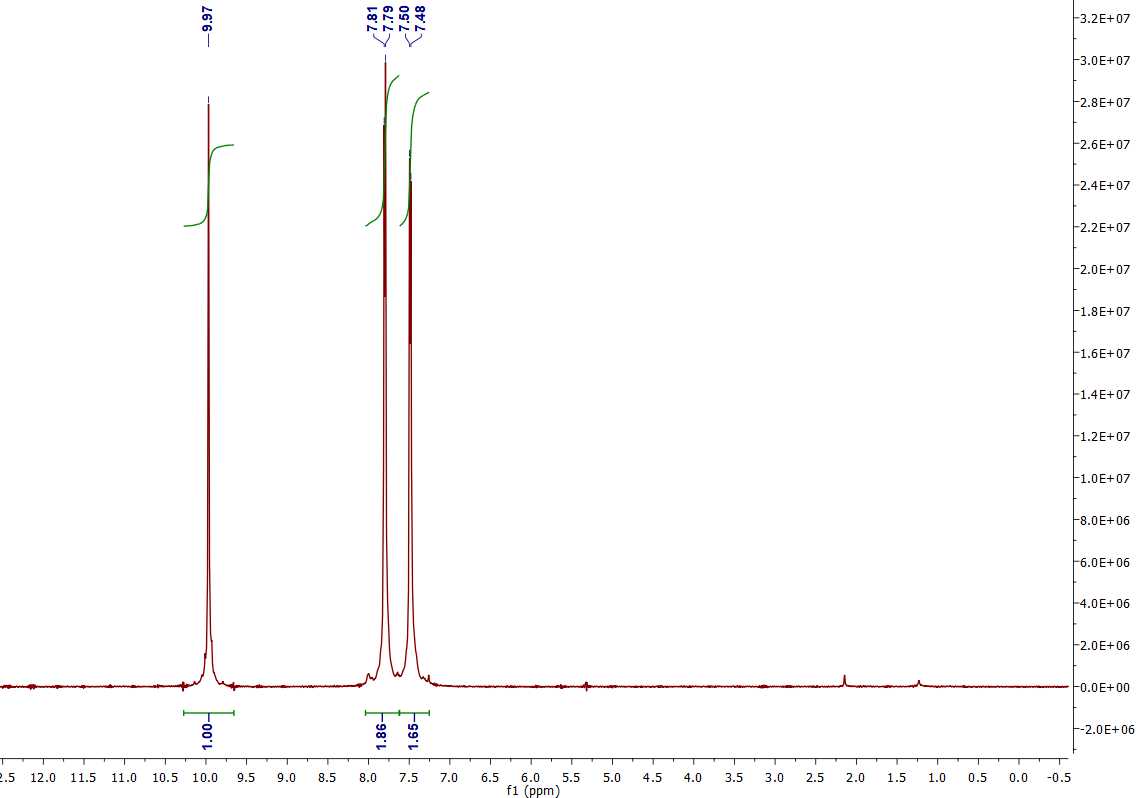  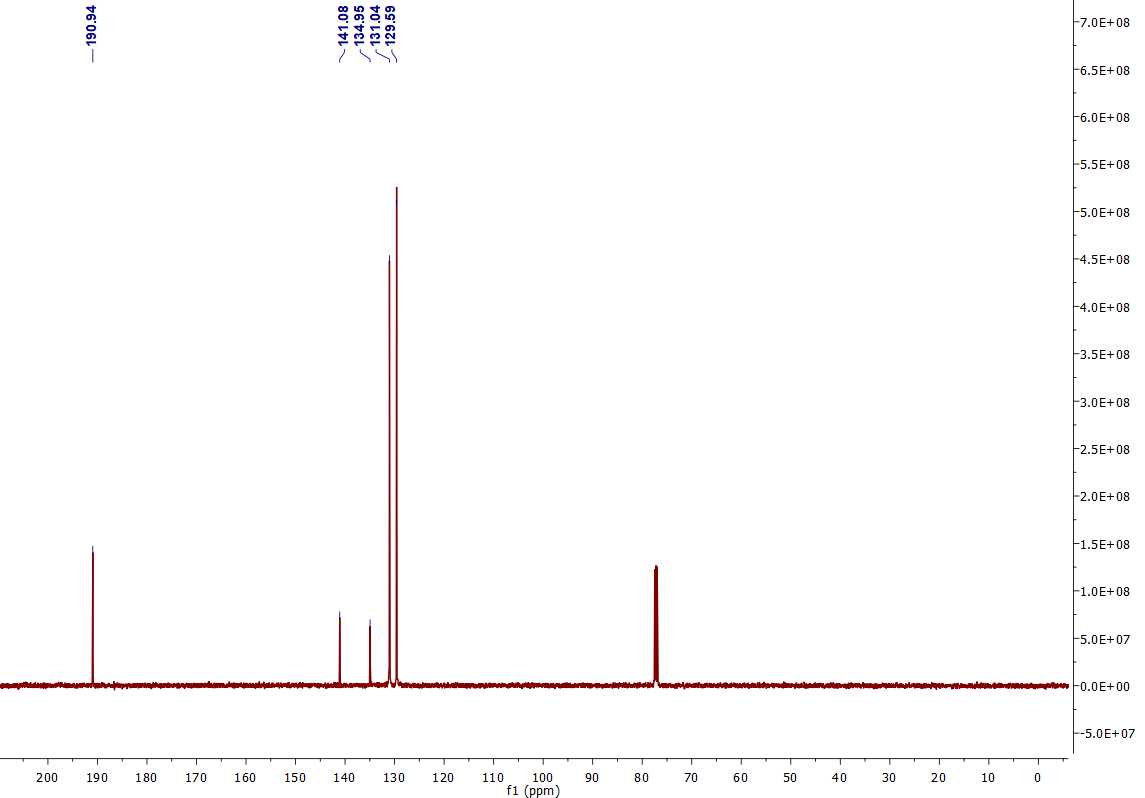  **Fig. S9:** ^1^H NMR and ^13^C NMR of 4-Chlorobenzaldehyde (2c) |
| --- |

| 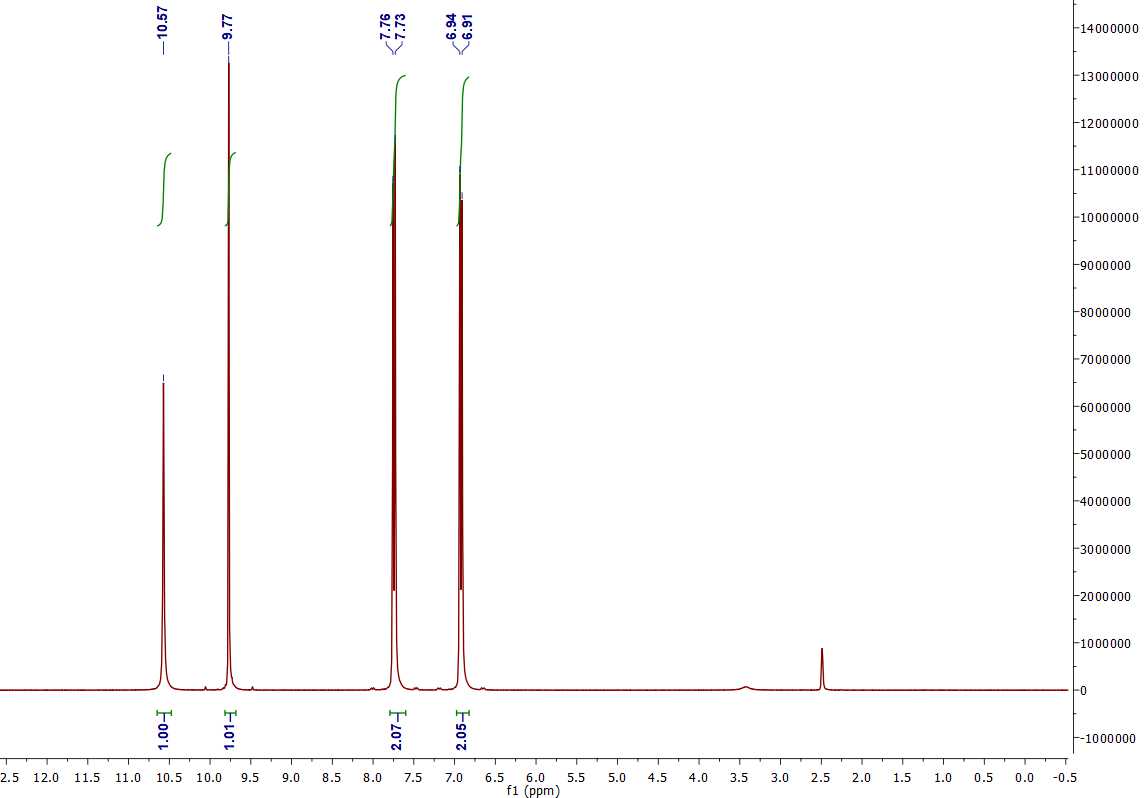  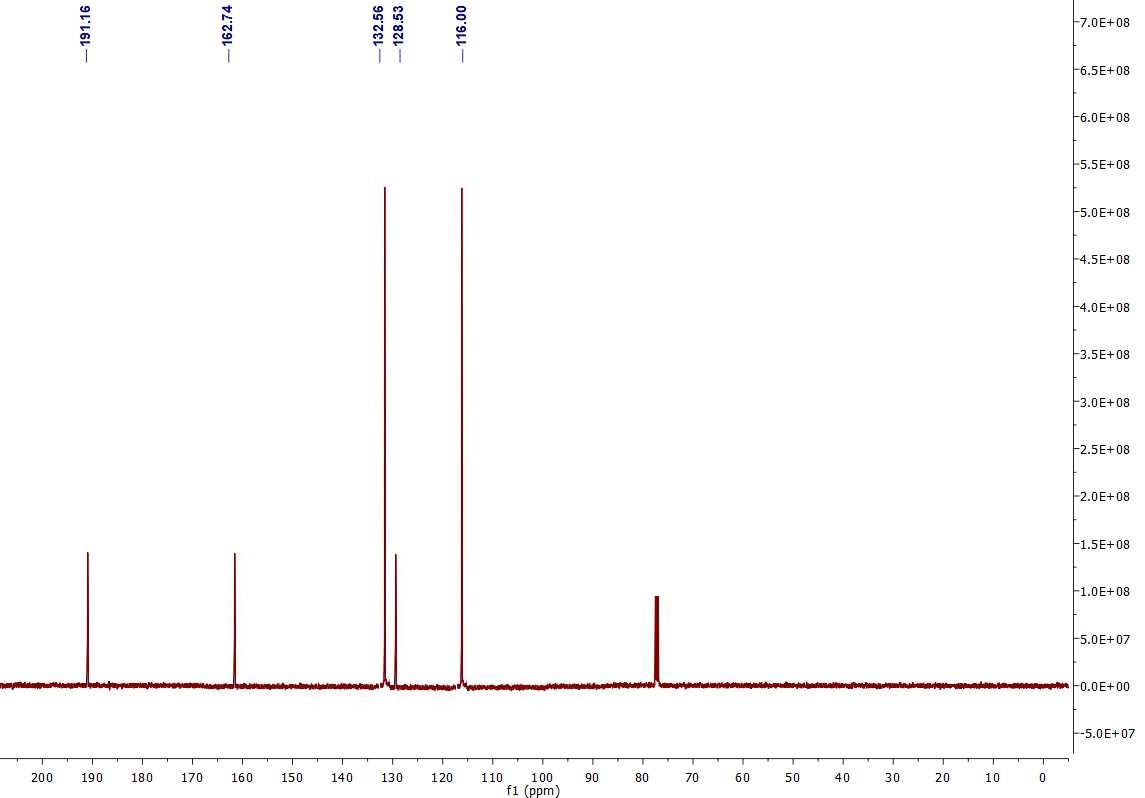  **Fig. S10:** ^1^H NMR and ^13^C NMR of 4-Hydroxybenzaldehyde (2d) |
| --- |

| 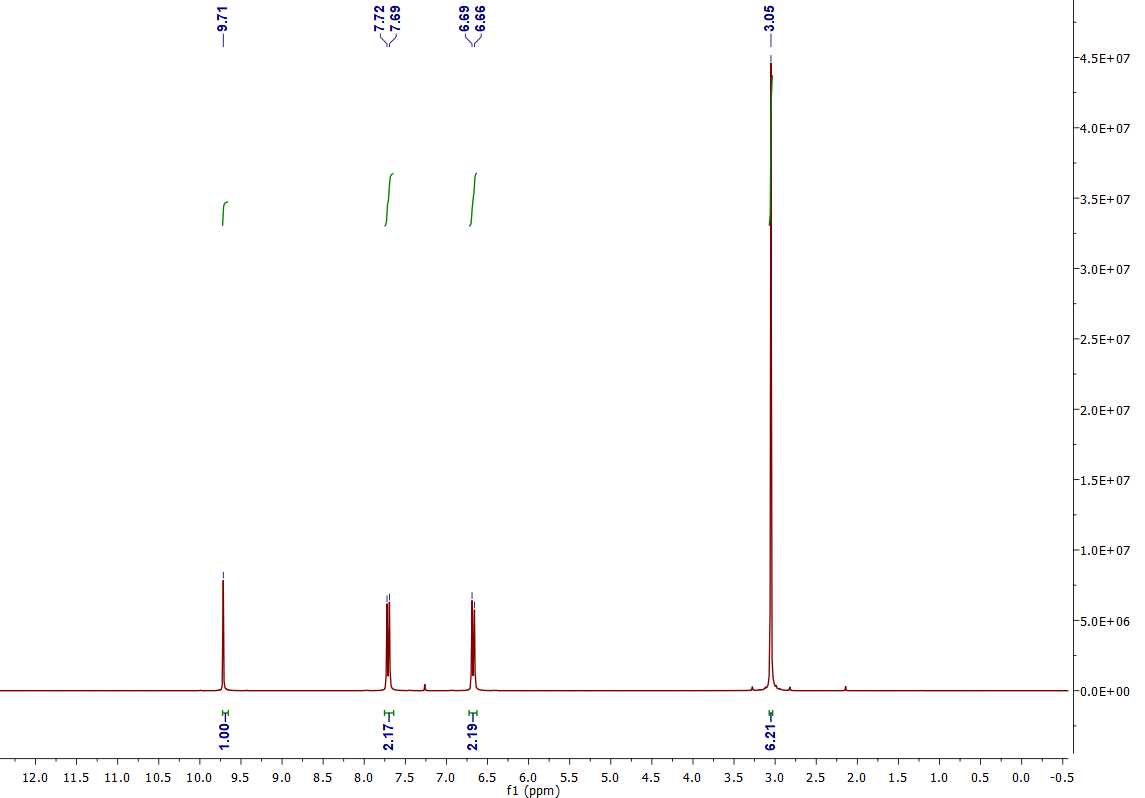  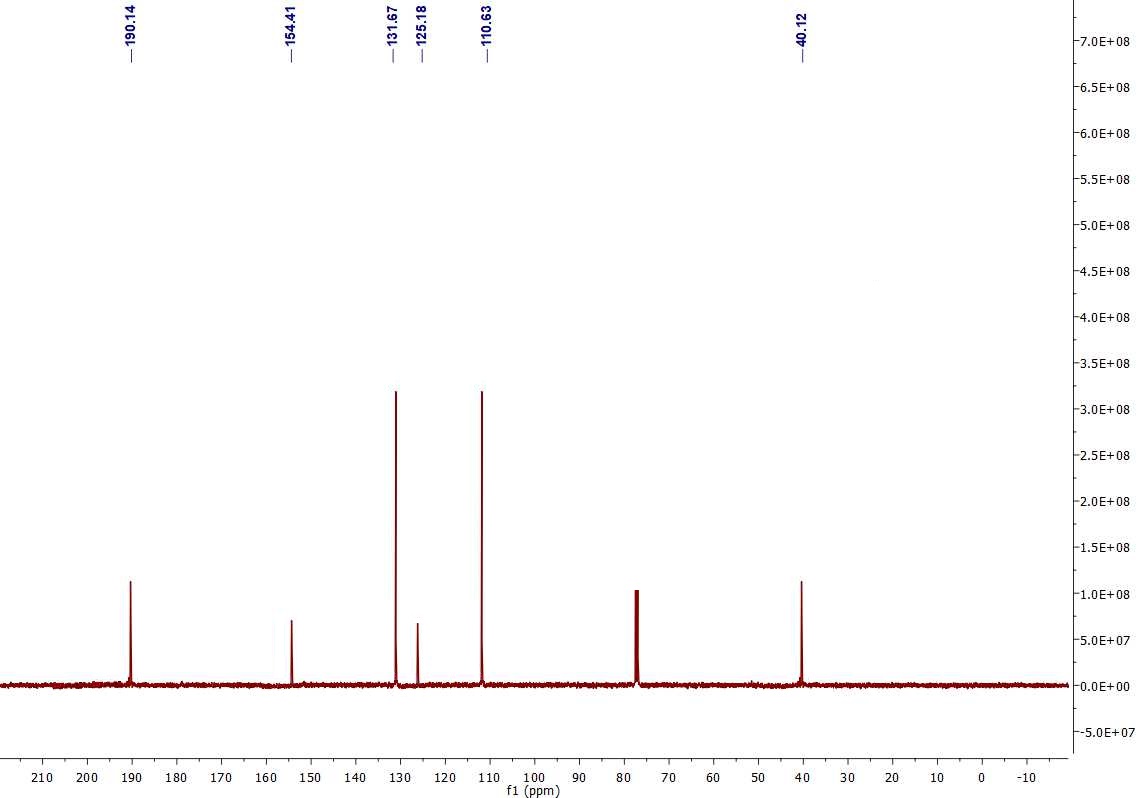  **Fig. S11:** ^1^H NMR and ^13^C NMR of 4-(Dimethylamino)benzaldehyde (2e) |
| --- |

| 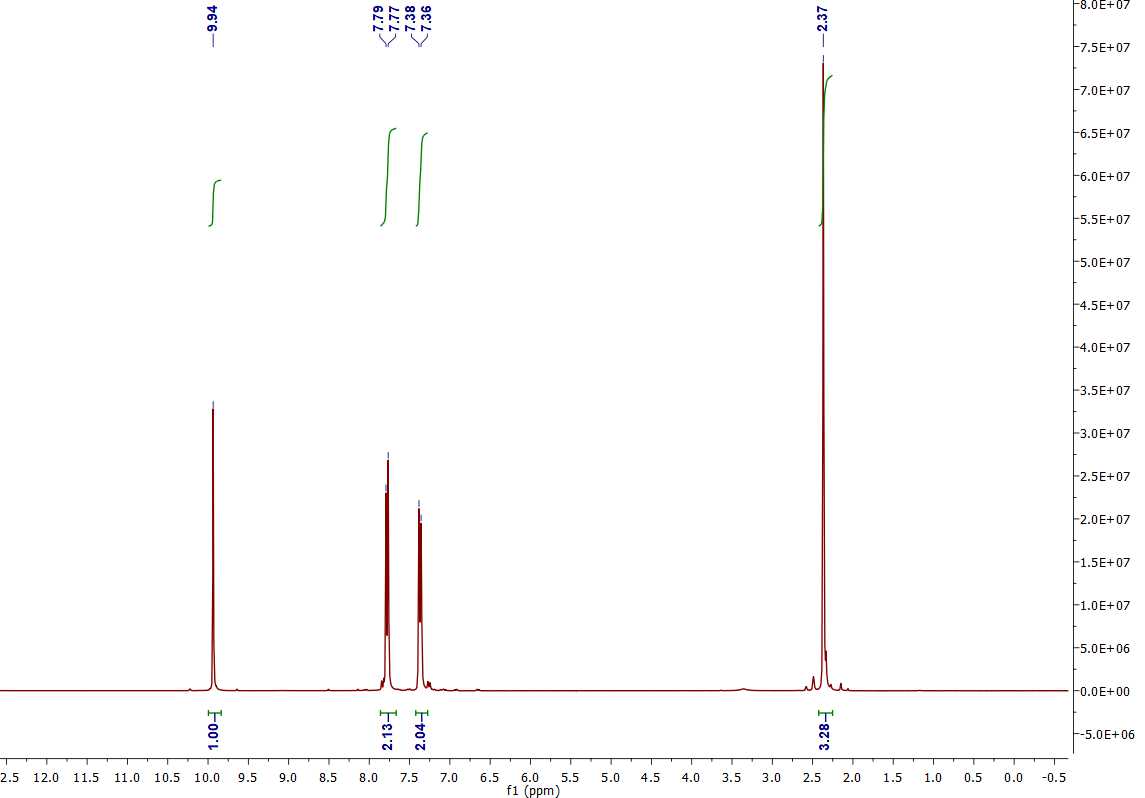  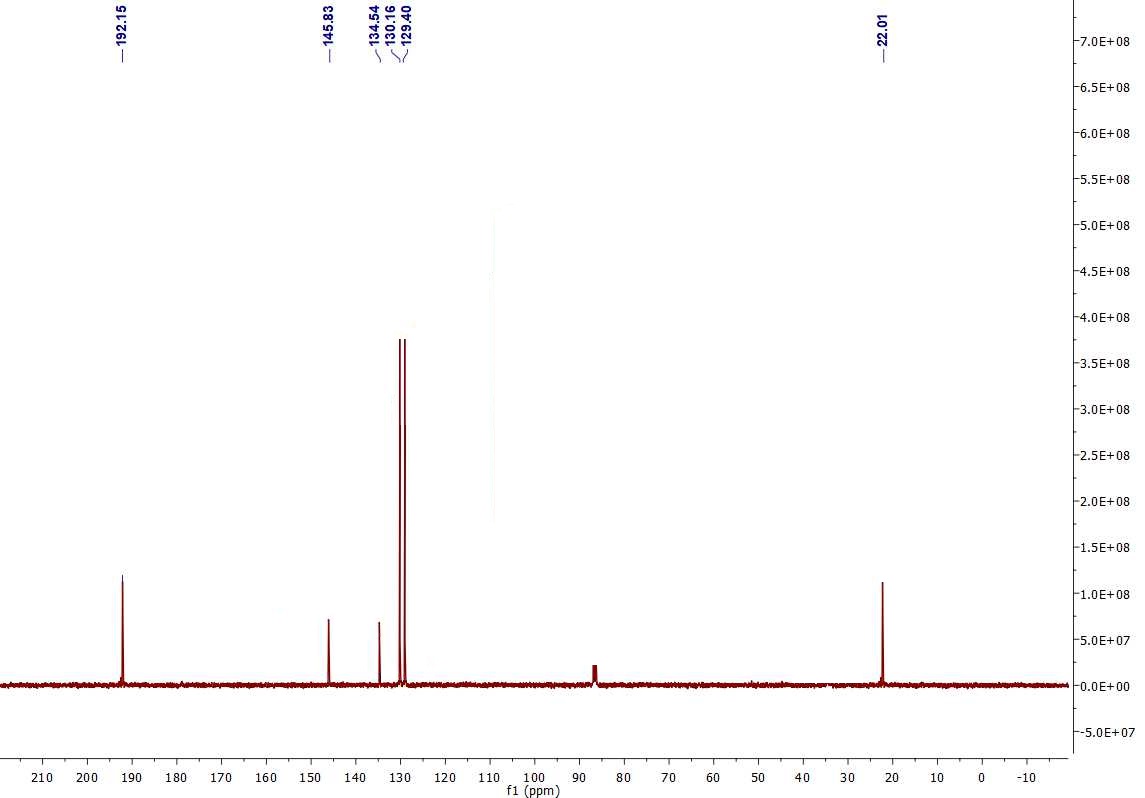  **Fig. S12:** ^1^H NMR and ^13^C NMR of 4-Methylbenzaldehyde (2f) |
| --- |

| 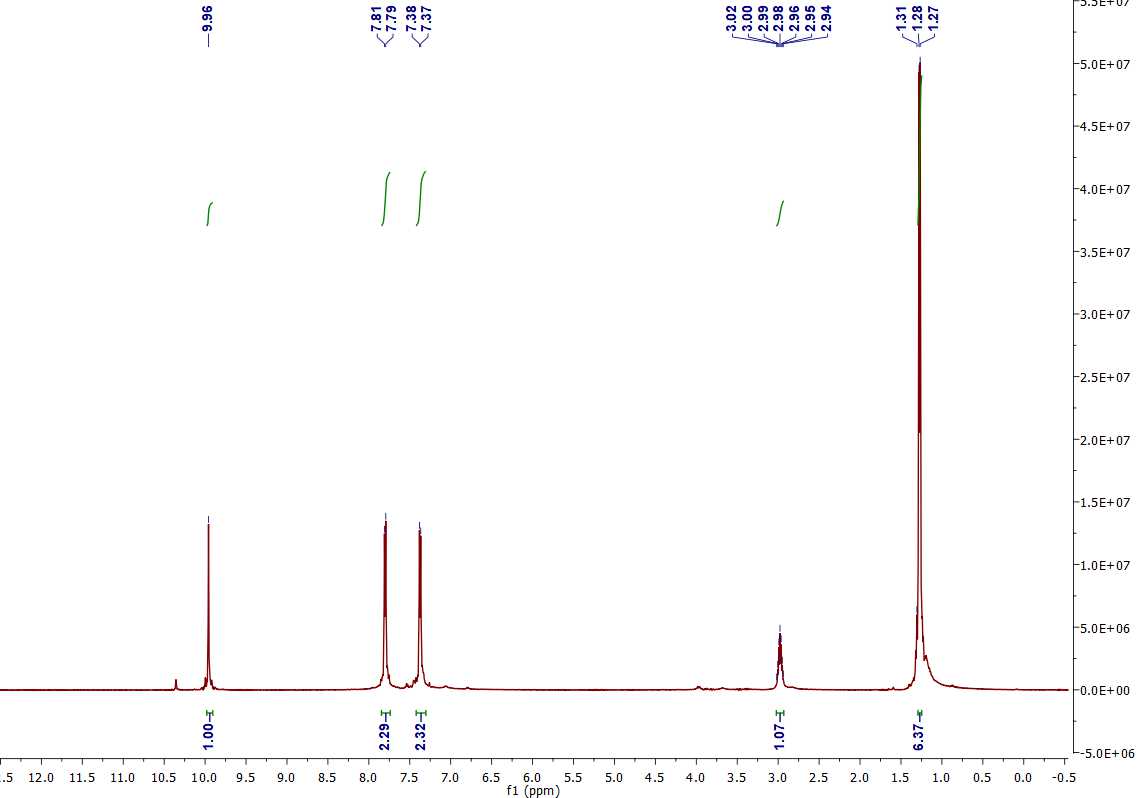  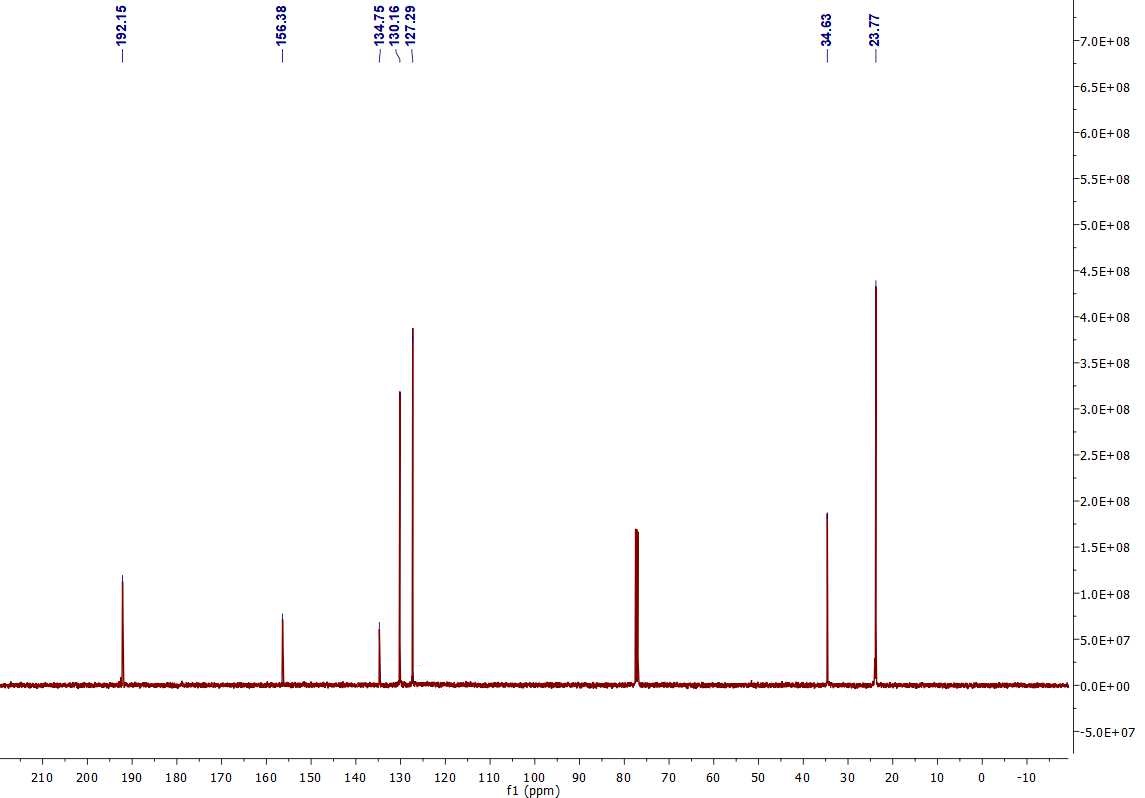  **Fig. S13:** ^1^H NMR and ^13^C NMR of 4-Isopropylbenzaldehyde (2g) |
| --- |

| 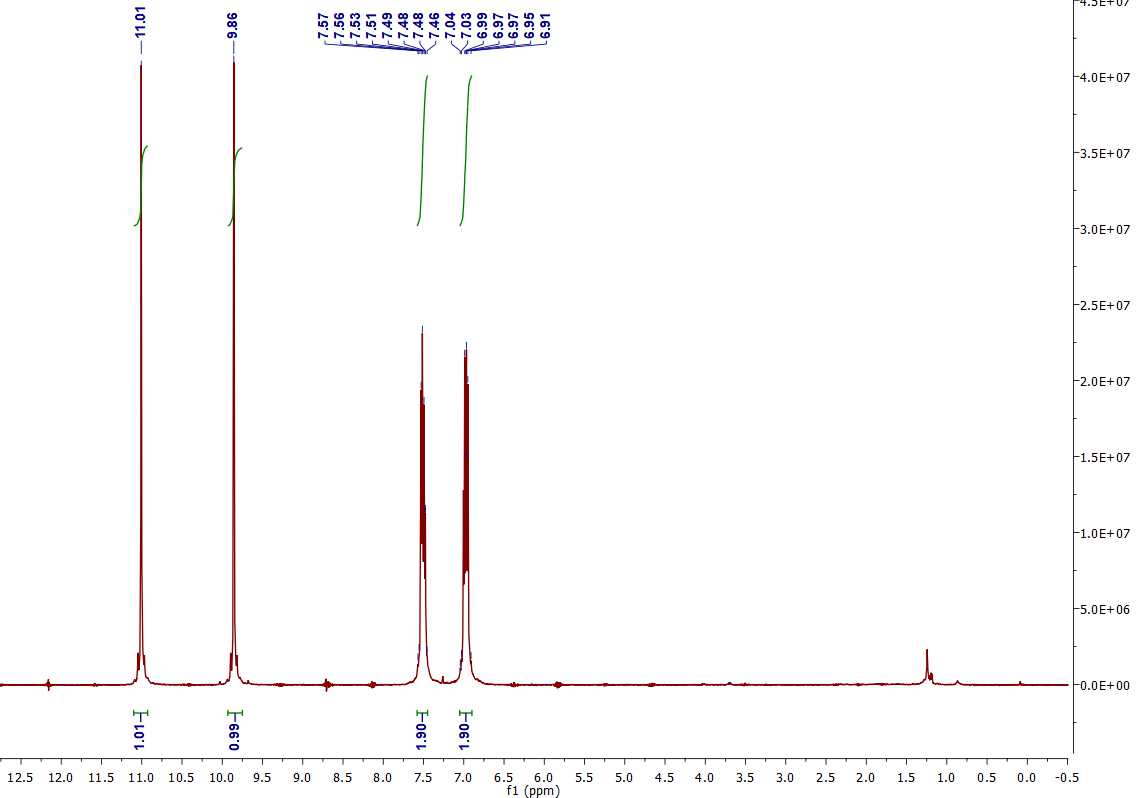  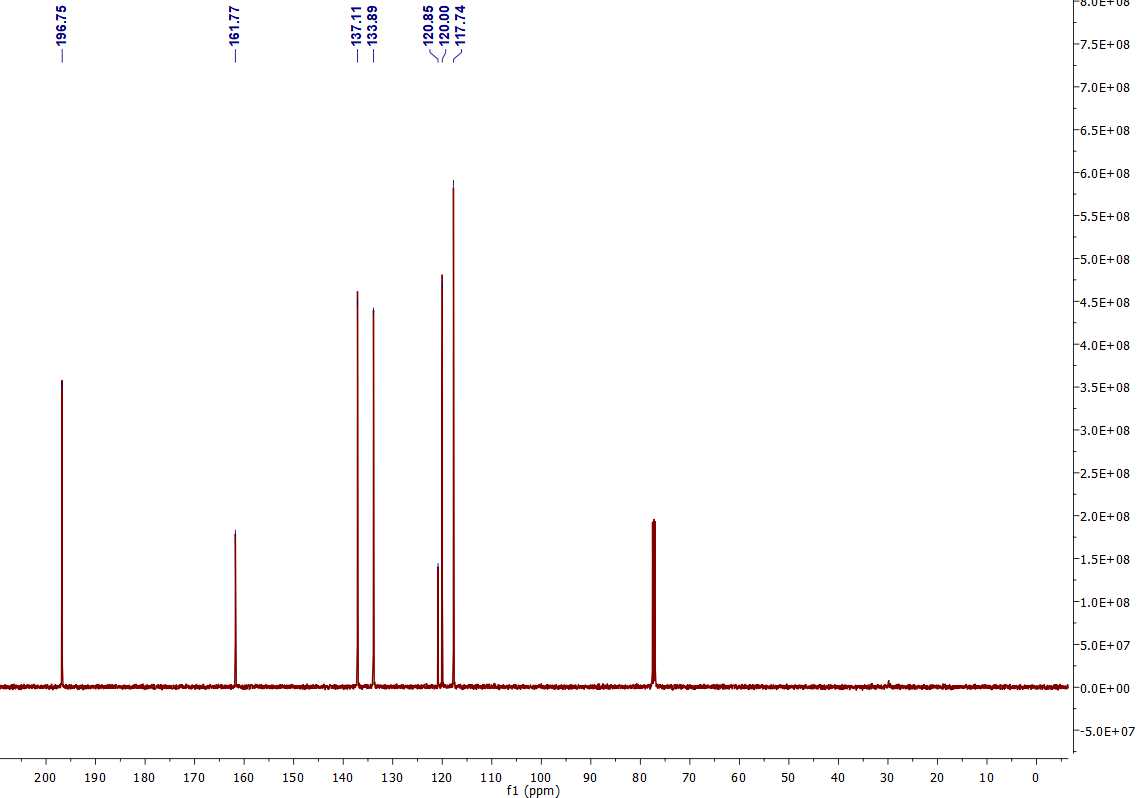  **Fig. S14:** ^1^H NMR and ^13^C NMR of 2-Hydroxybenzaldehyde (2h) |
| --- |

| 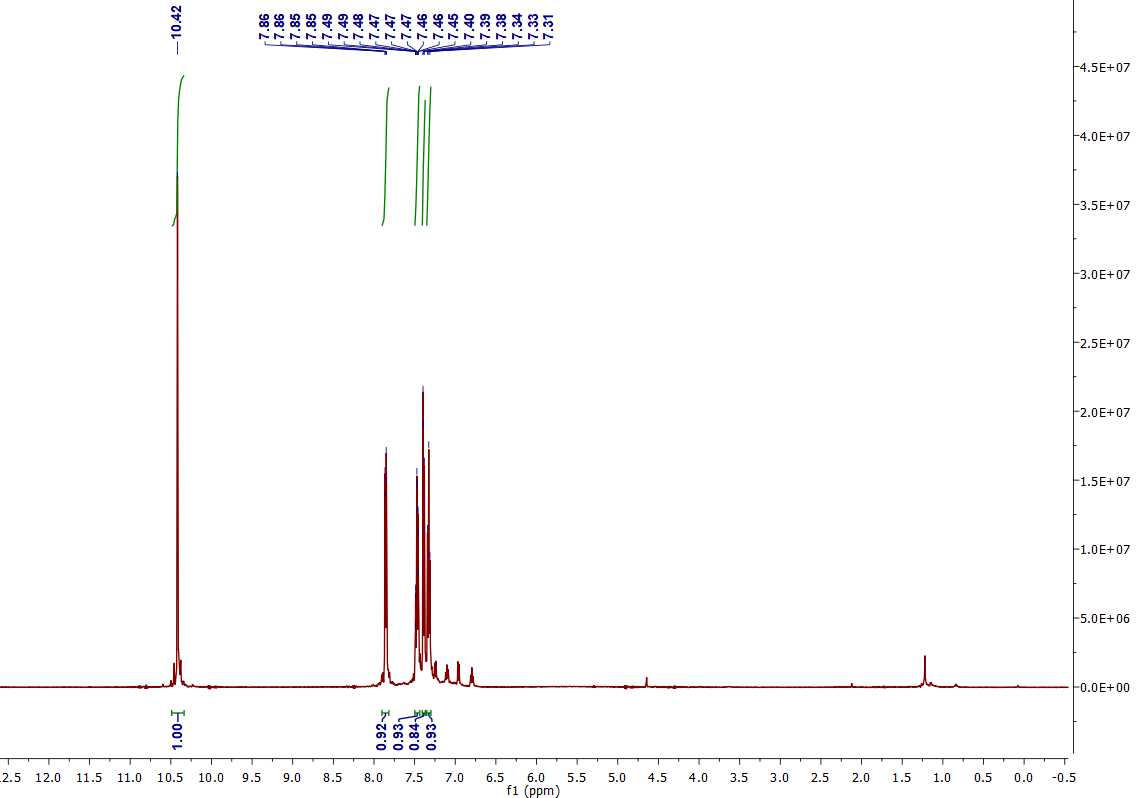  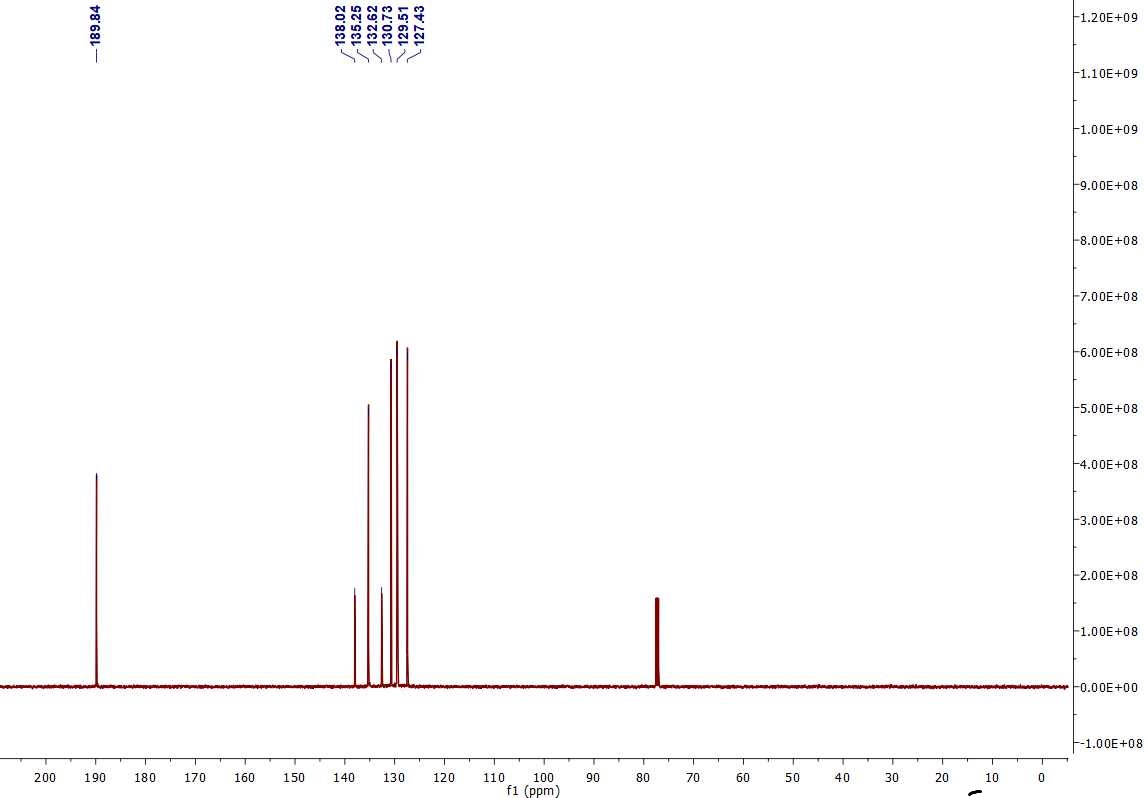  **Fig. S15:** ^1^H NMR and ^13^C NMR of 2-Chlorobenzaldehyde (2i) |
| --- |

| 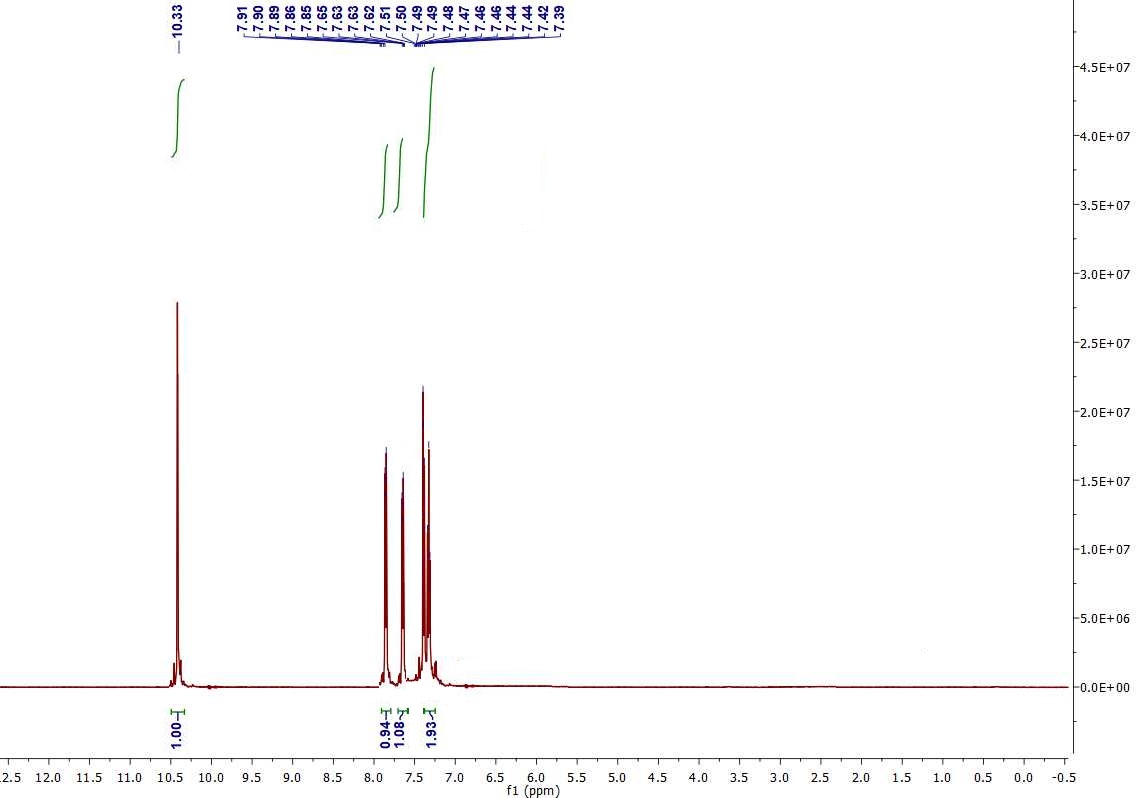  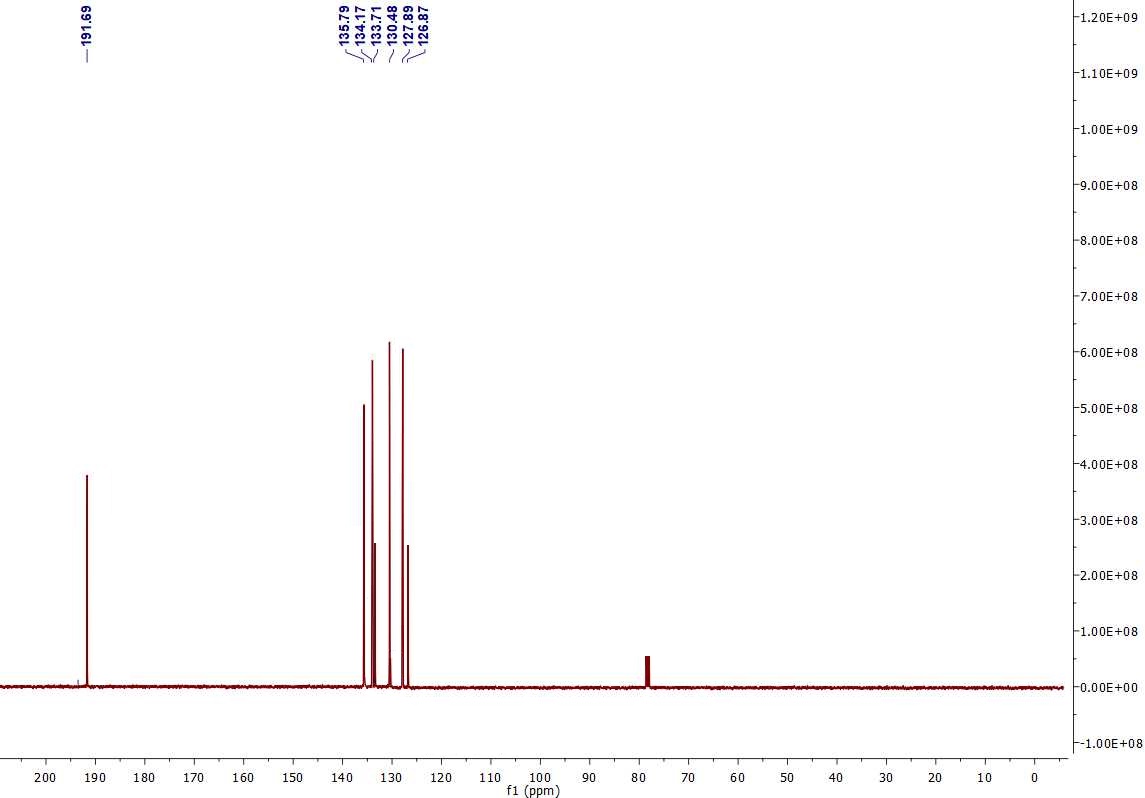  **Fig. S16:** ^1^H NMR and ^13^C NMR of 2-Bromobenzaldehyde (2j) |
| --- |

| 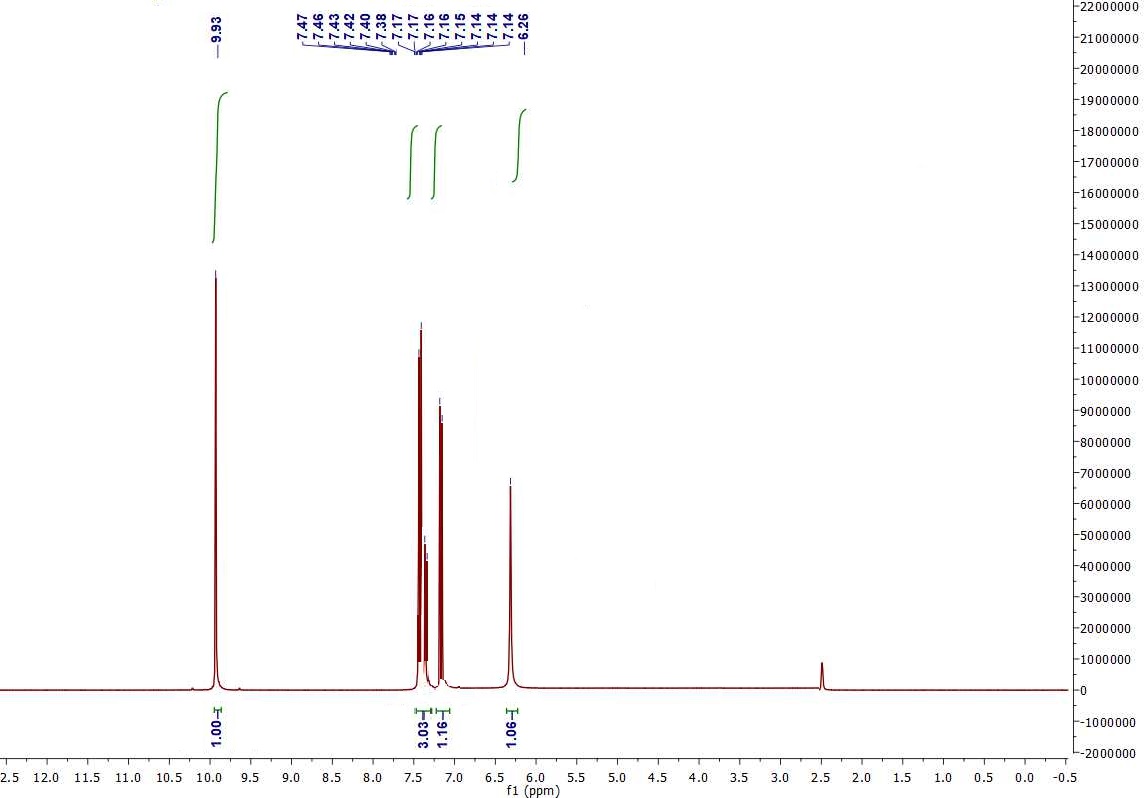  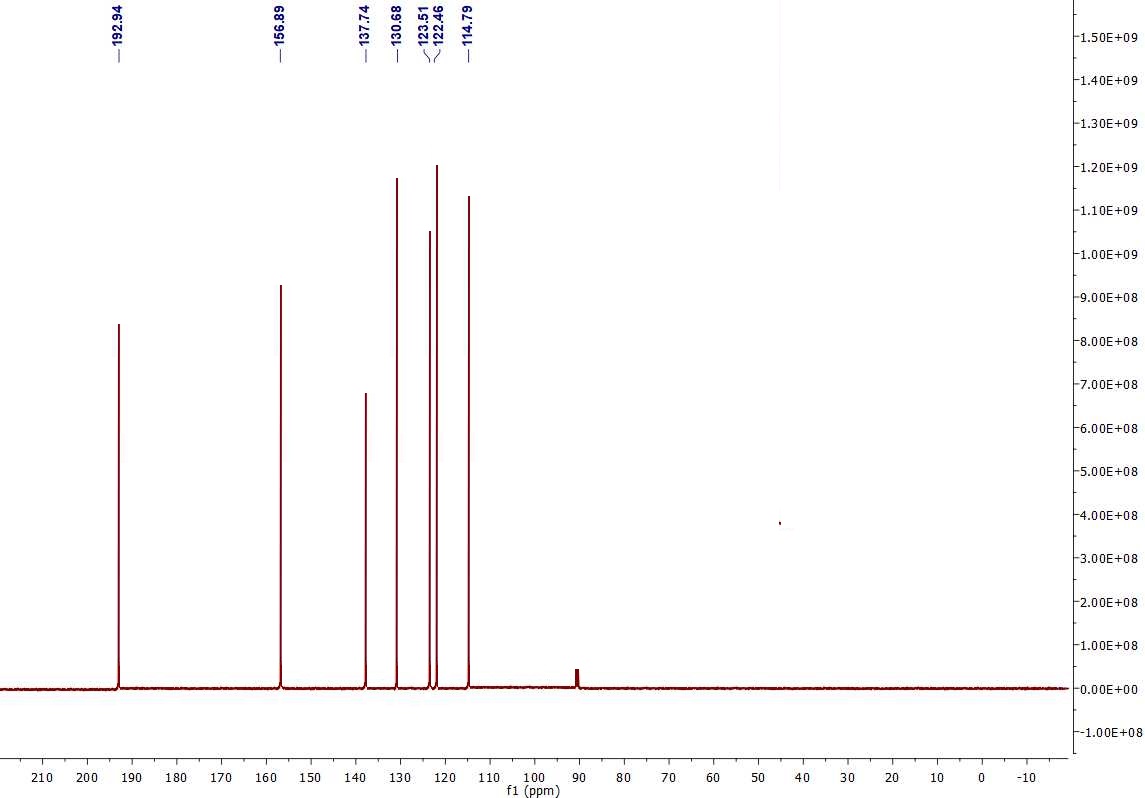  **Fig. S17:** ^1^H NMR and ^13^C NMR of 3-Hydroxybenzaldehyde (2k) |
| --- |

| 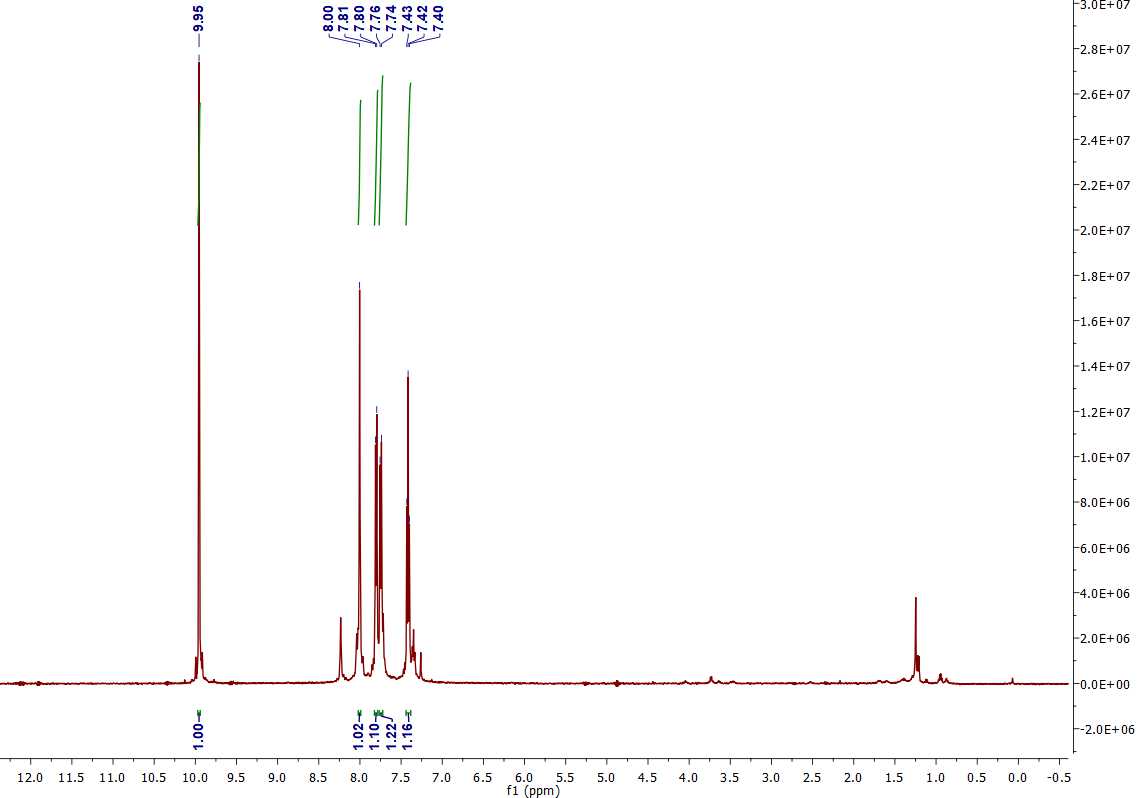  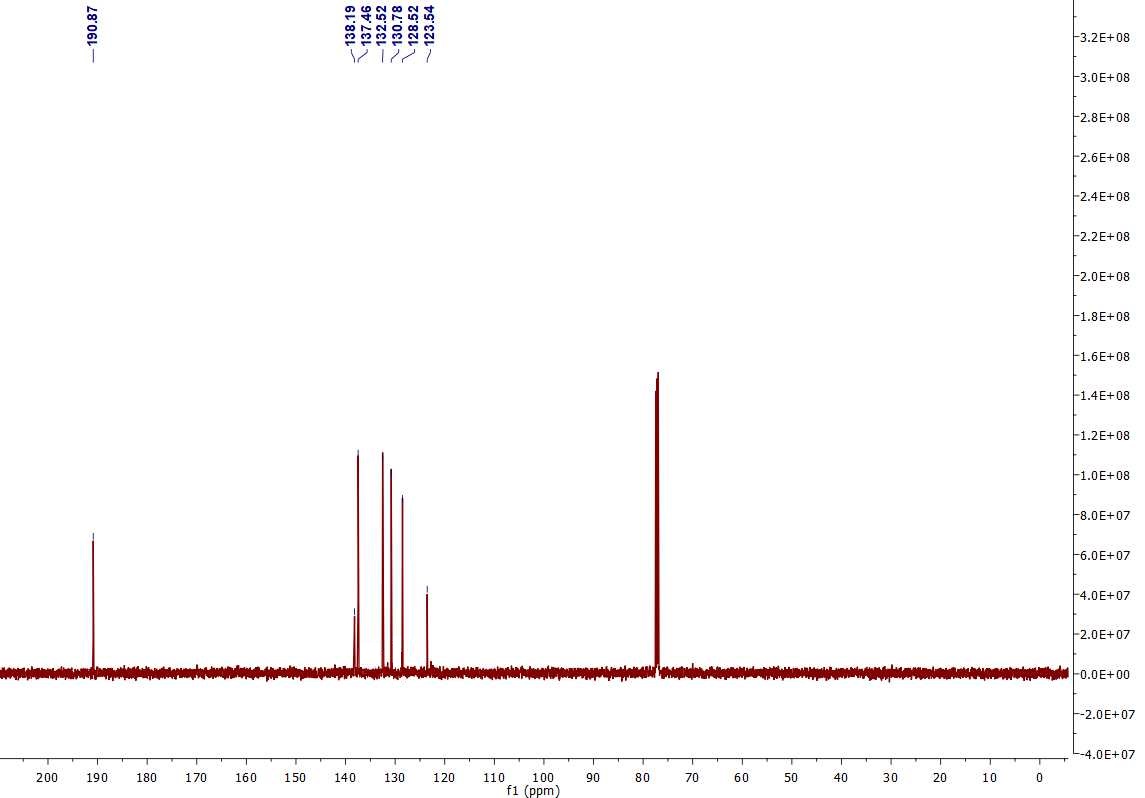  **Fig. S18:** ^1^H NMR and ^13^C NMR of 3-Bromobenzaldehyde (2l) |
| --- |

| 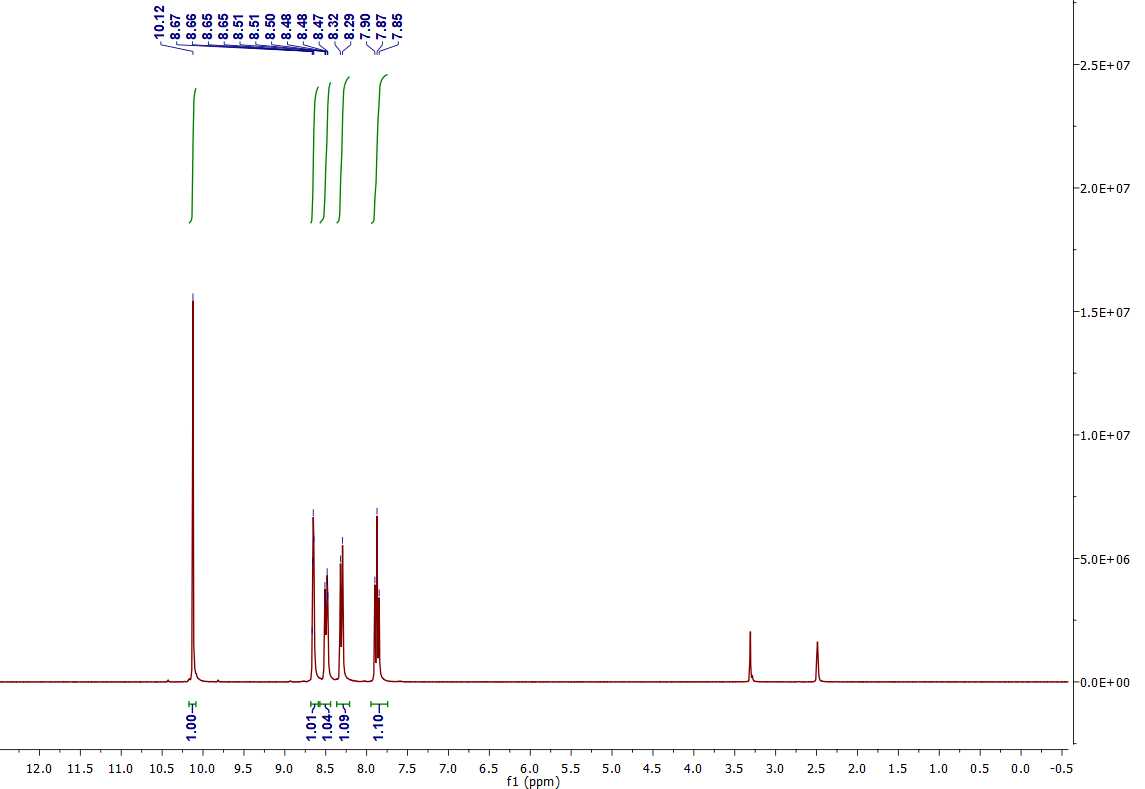  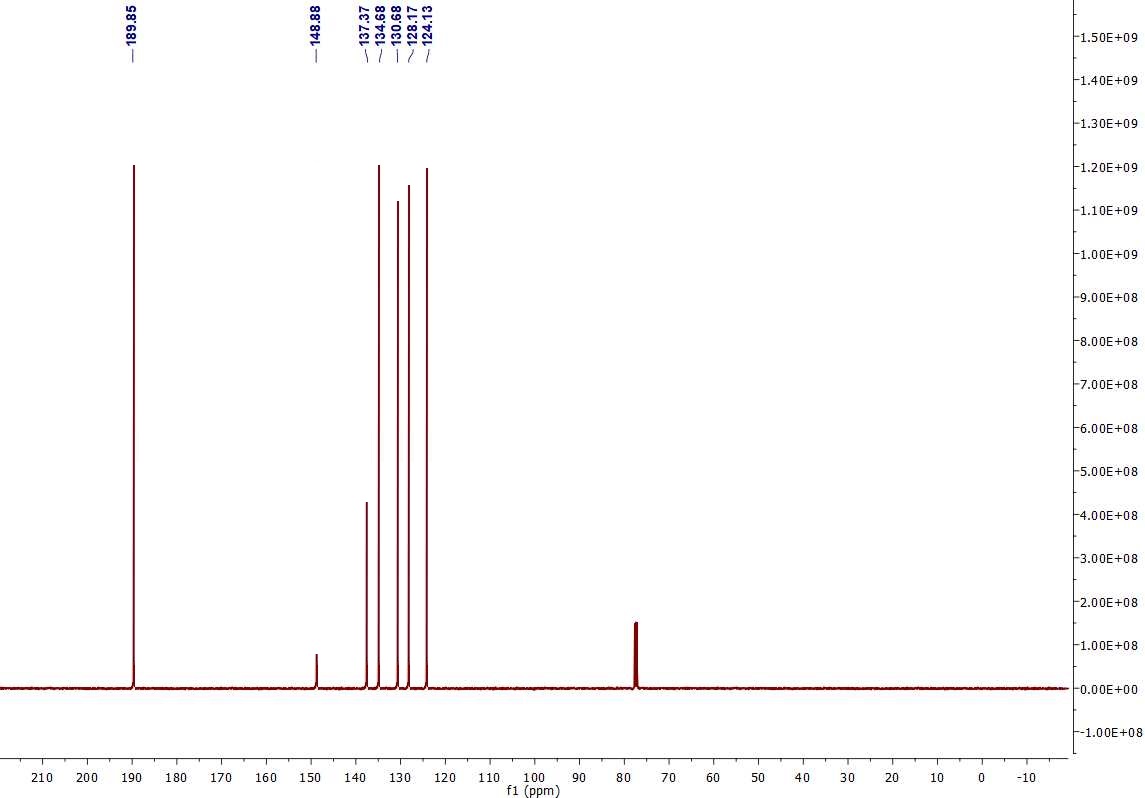  **Fig. S19:** ^1^H NMR and ^13^C NMR of 3-Nitrobenzaldehyde (2m) |
| --- |

| **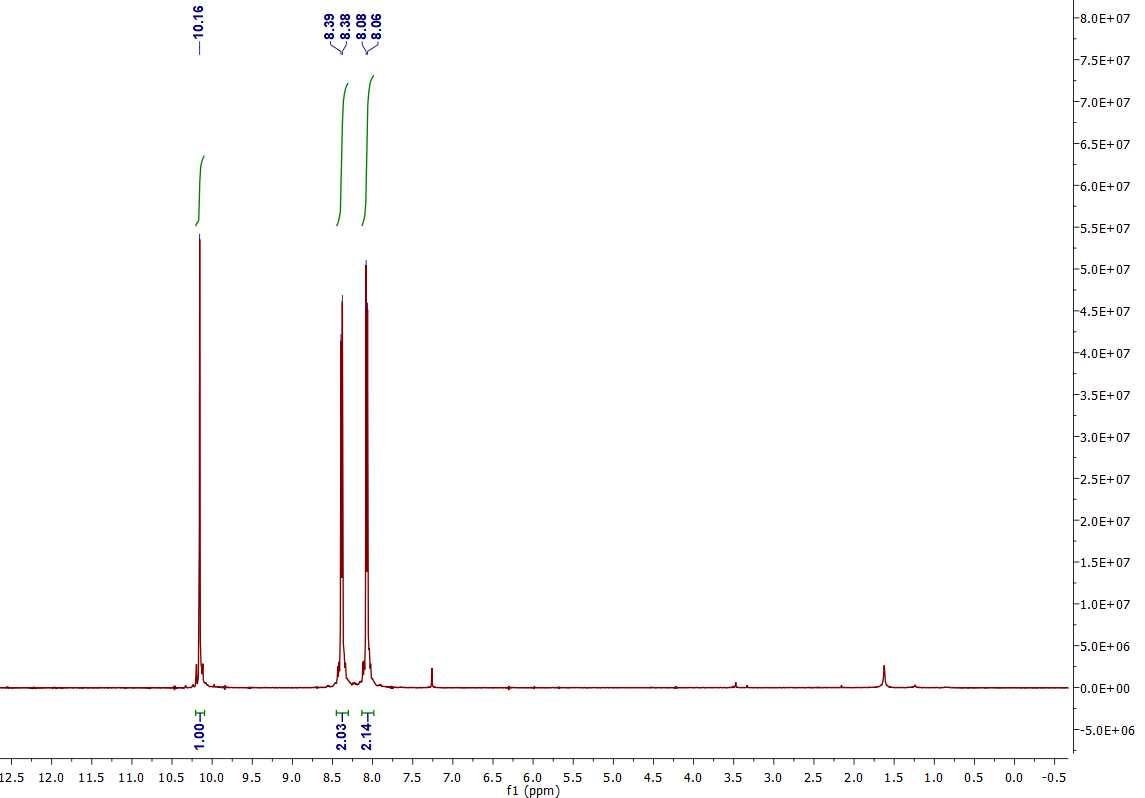**  **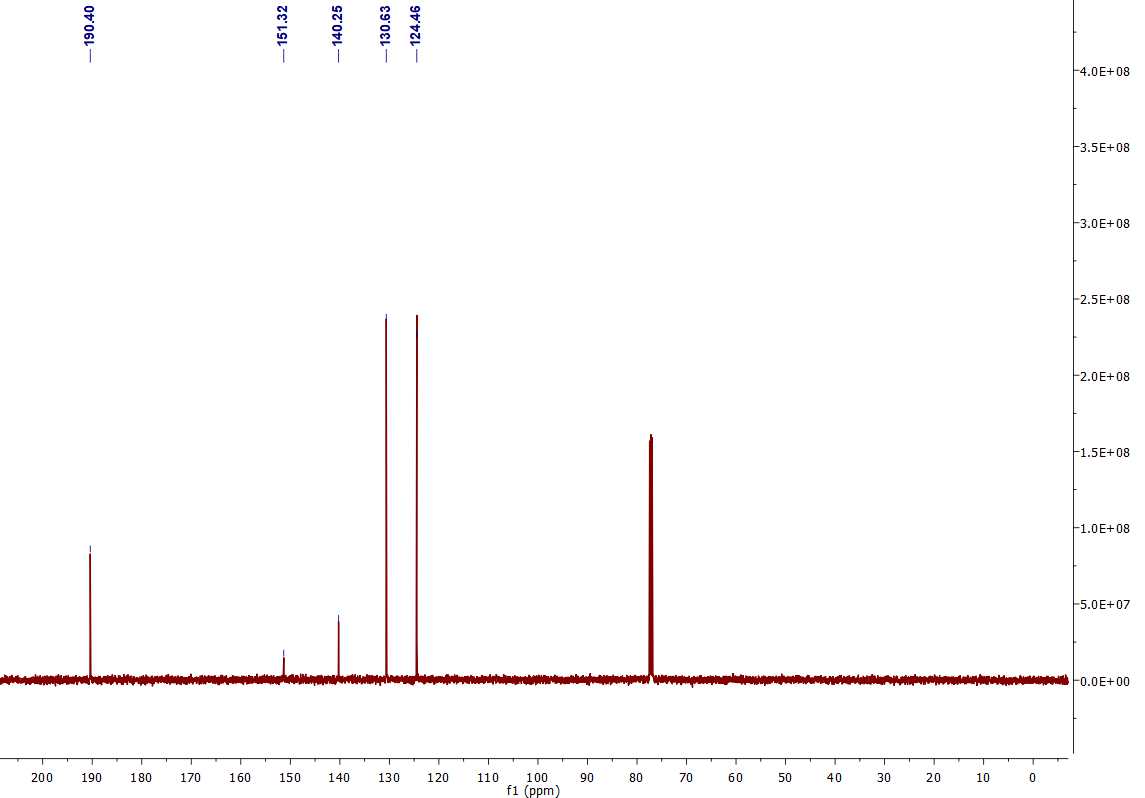**  **Fig. S20:** ^1^H NMR and ^13^C NMR of 4-Nitrobenzaldehyde (2n) |
| --- |

| **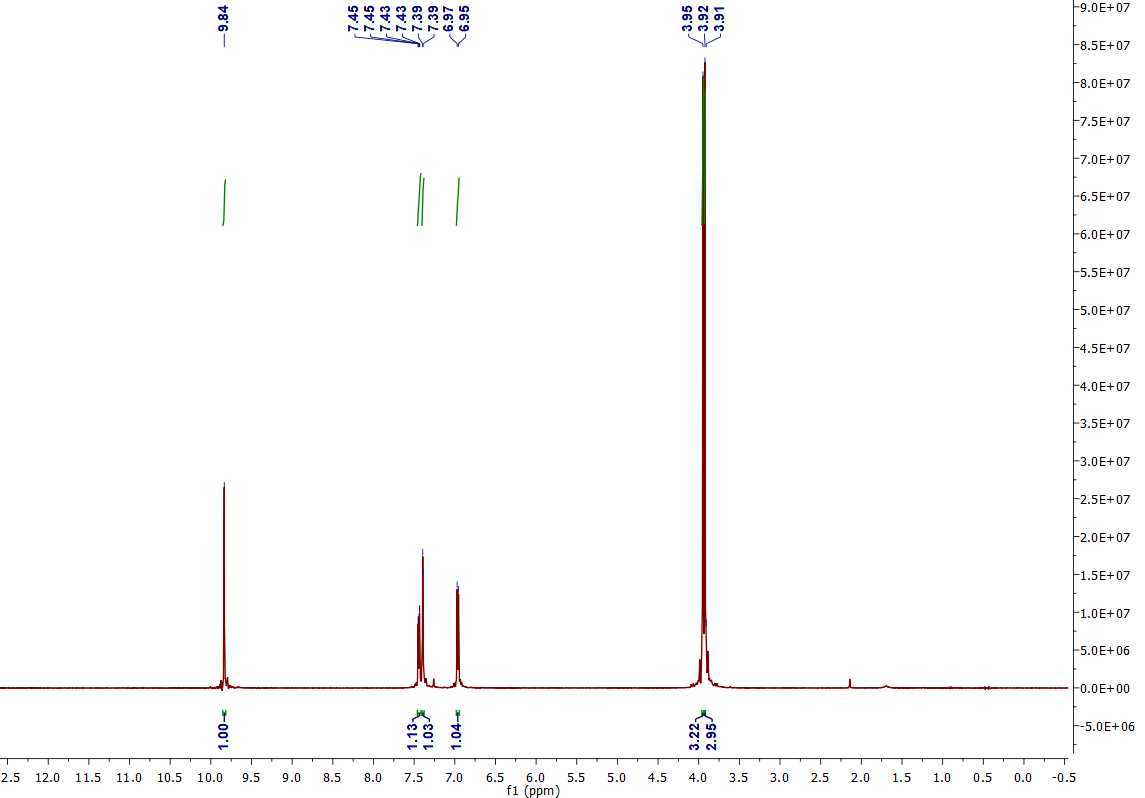**  **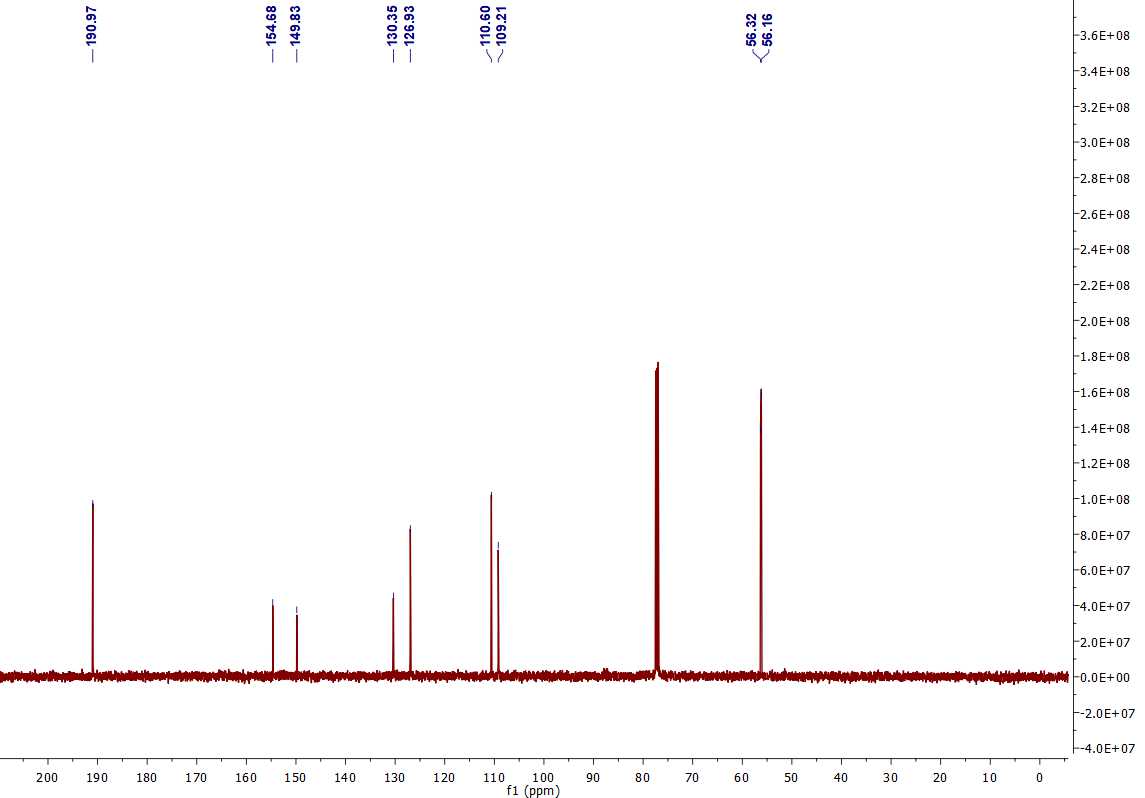**  **Fig. S21:** ^1^H NMR and ^13^C NMR of 3,4-Dimethoxybenzaldehyde (2o) |
| --- |

| **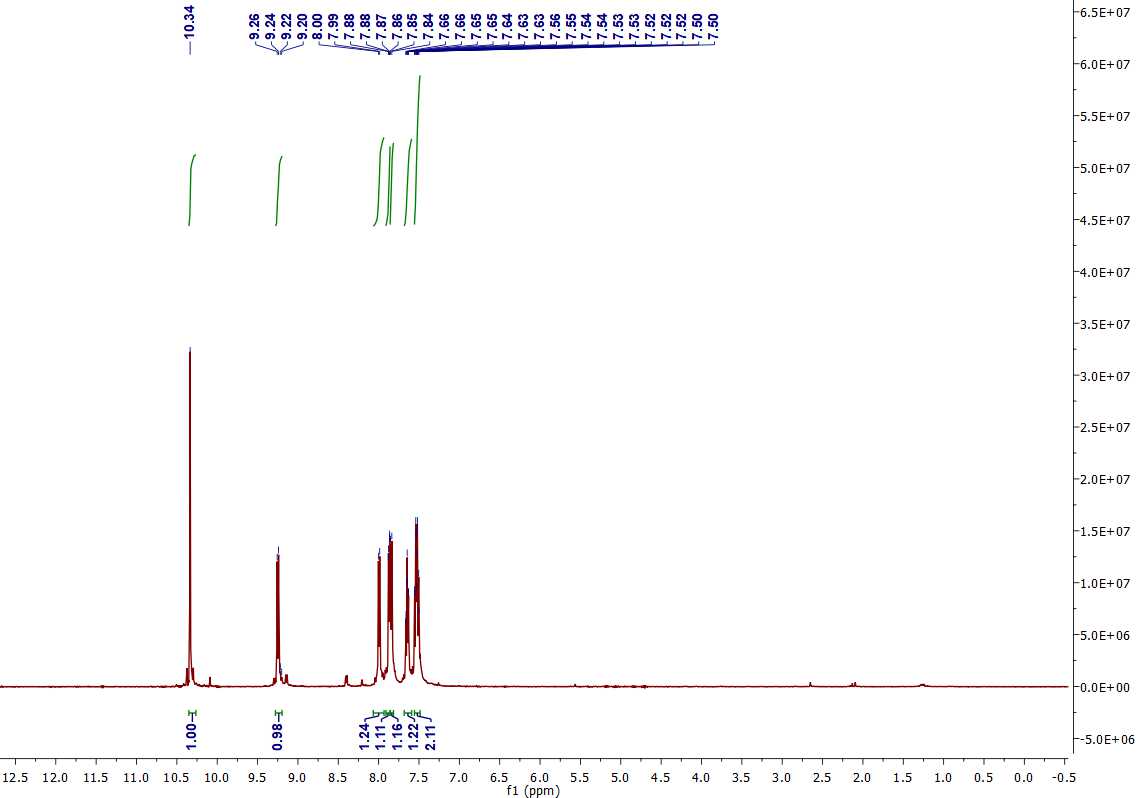**  **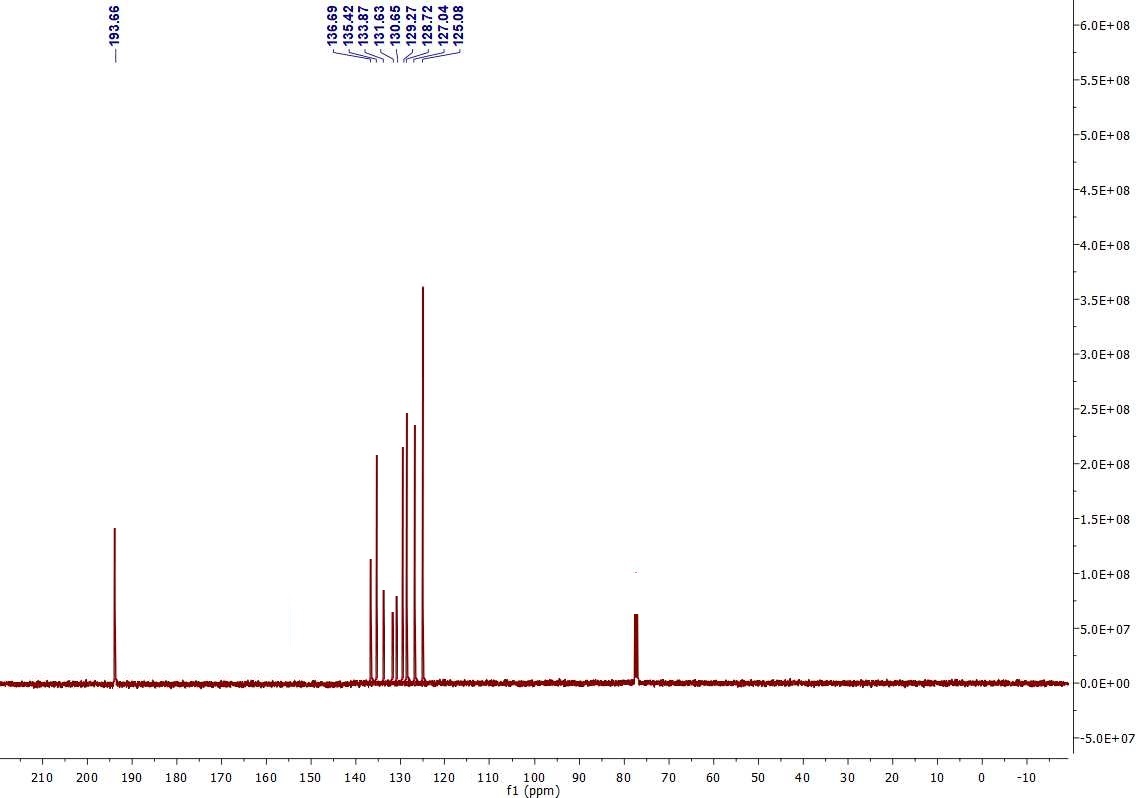**  **Fig. S22:** ^1^H NMR and ^13^C NMR of 1-Naphthaldehyde (2p) |
| --- |

| **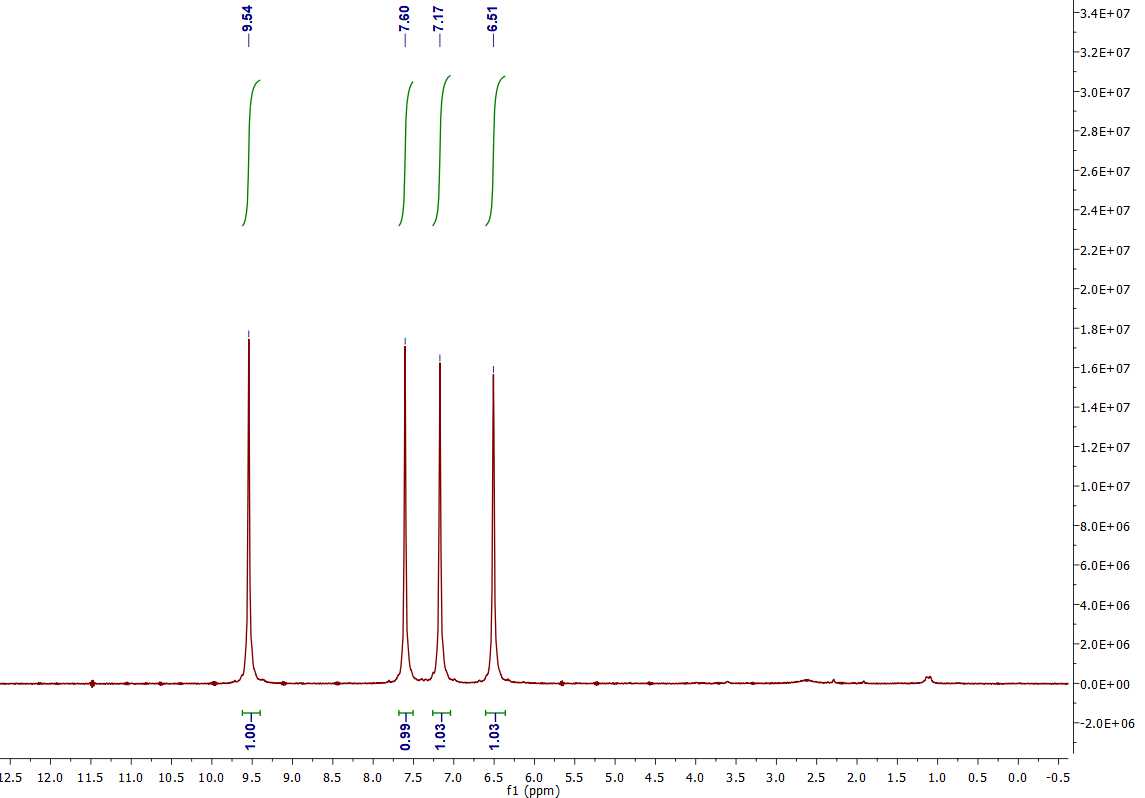**  **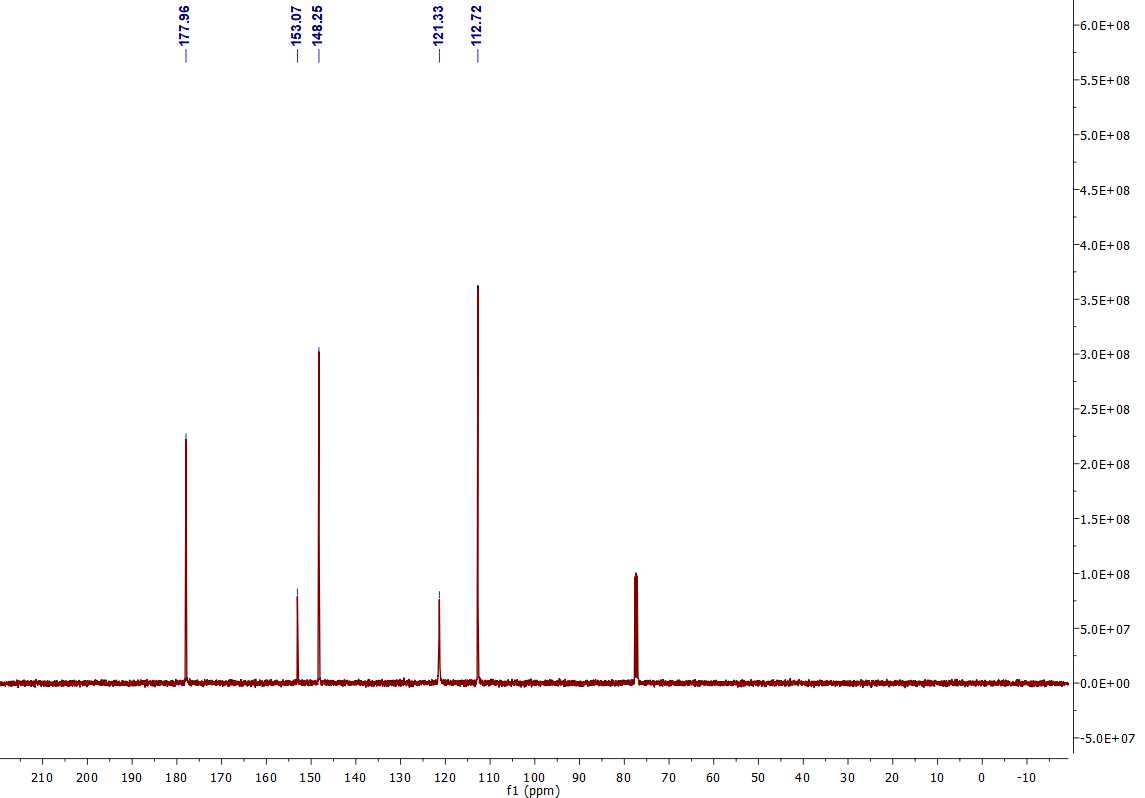**  **Fig. S23:** ^1^H NMR and ^13^C NMR of Furan-2-carbaldehyde (2q) |
| --- |

**FT-IR spectrum of benzaldehyde derivatives:**

| **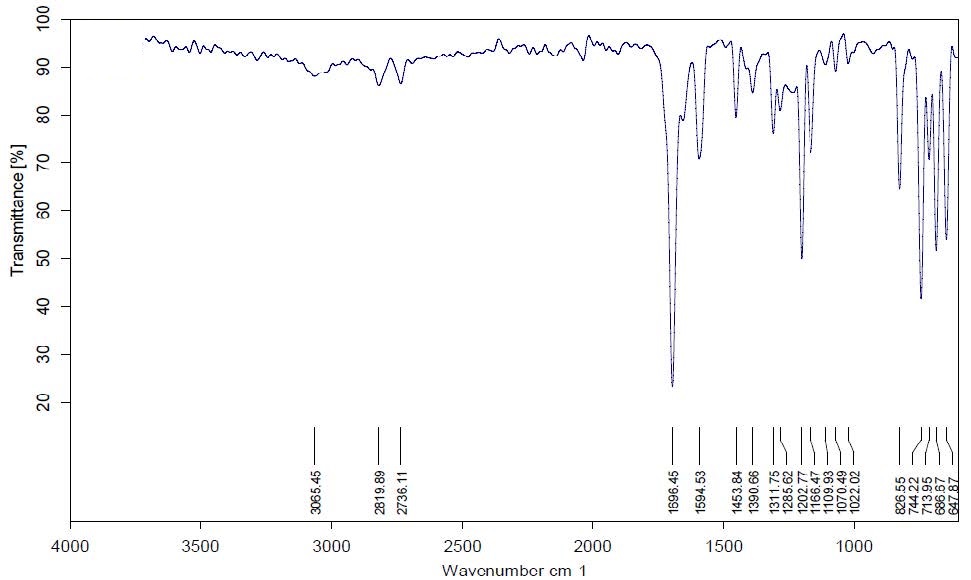**  **Fig. S24:** FT-IR spectrum of Benzaldehyde (2a) |
| --- |

| 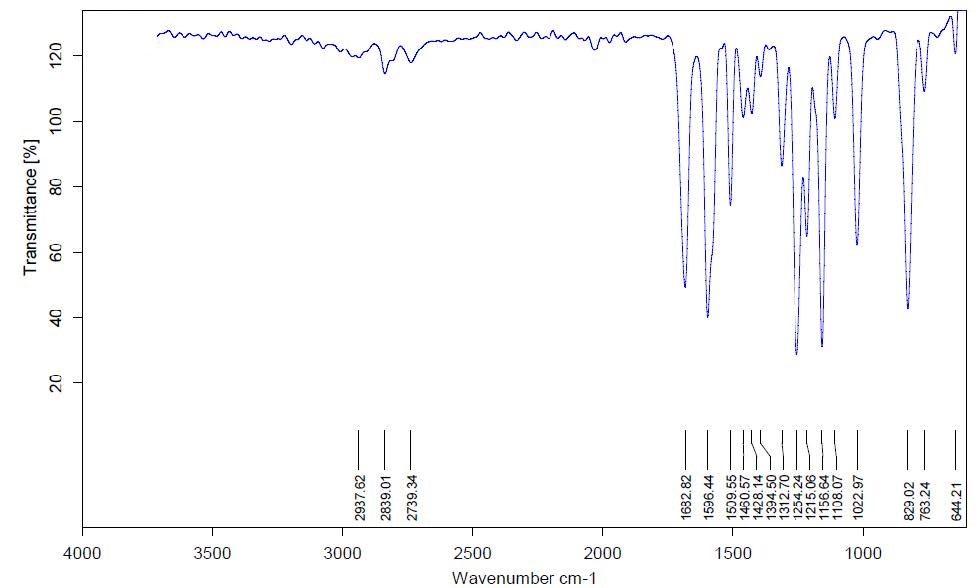  **Fig. S25:** FT-IR spectrum of 4-Methoxybenzaldehyde (2b) |
| --- |

| **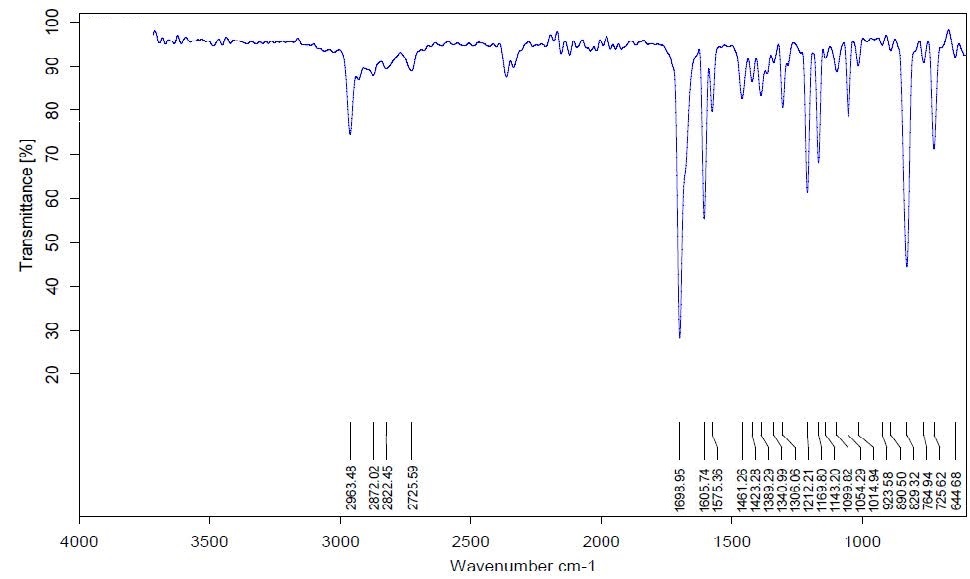**  **Fig. S26:** FT-IR spectrum of 4-Isopropylbenzaldehyde (2g) |
| --- |

| **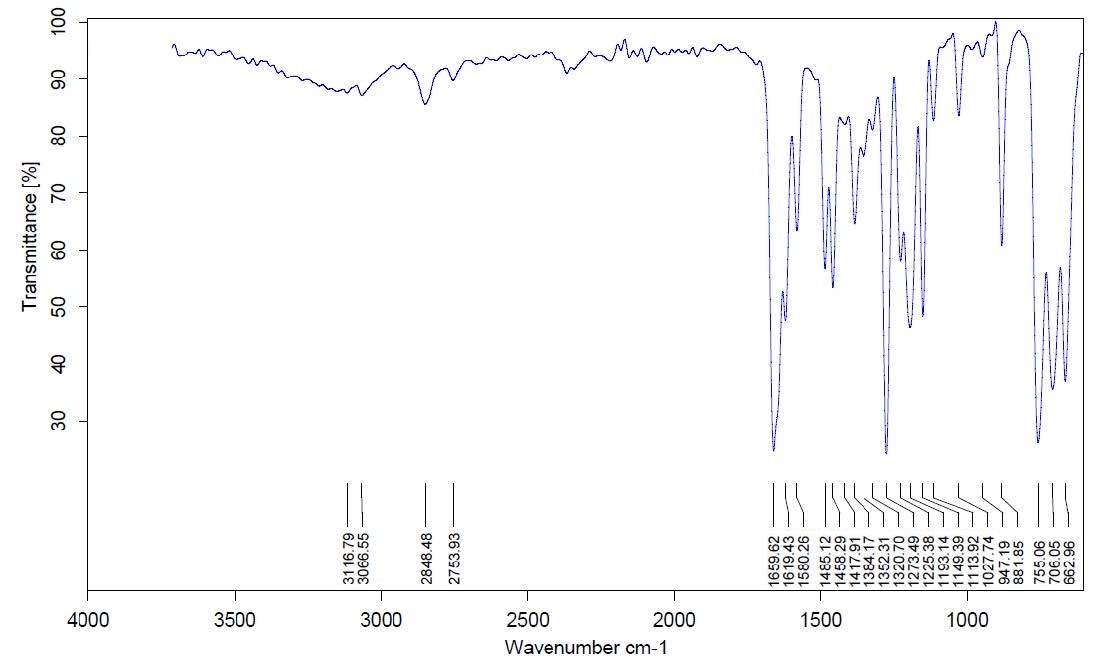**  **Fig. S27:** FT-IR spectrum of 2-Hydroxybenzaldehyde (2h) |
| --- |

| 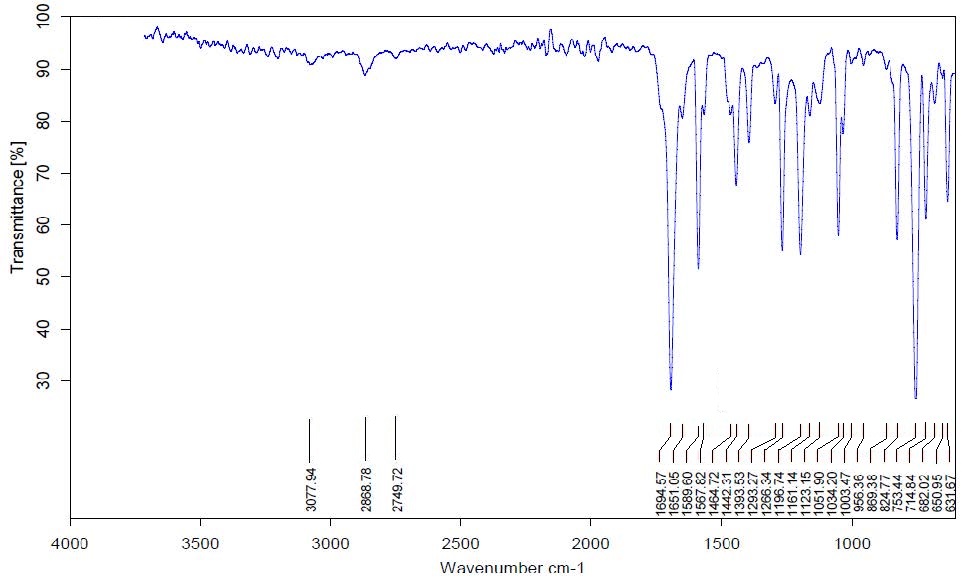  **Fig. S28:** FT-IR spectrum of 2-Chlorobenzaldehyde (2i) |
| --- |

| **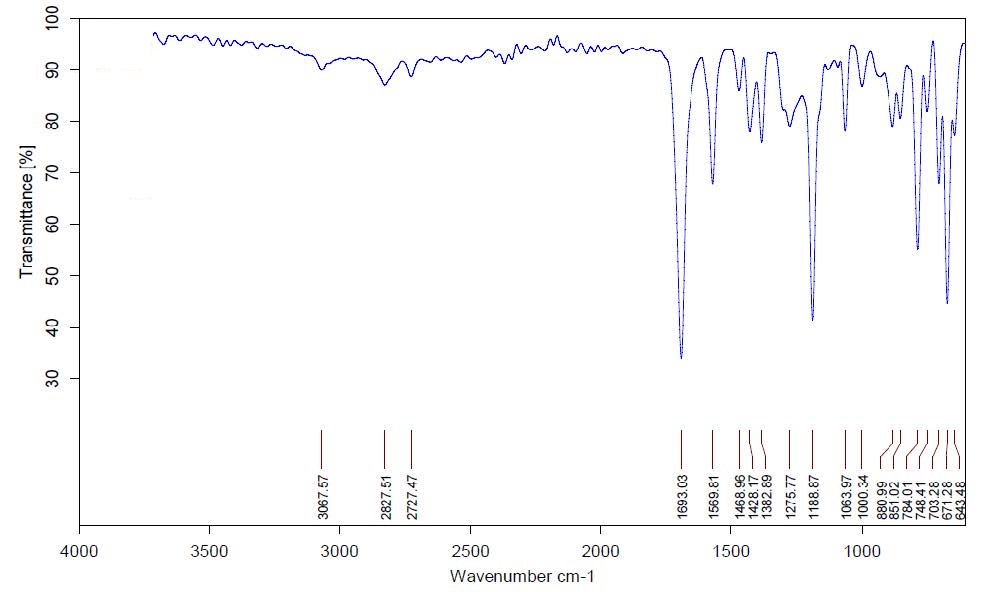**  **Fig. S29:** FT-IR spectrum of 3-Bromobenzaldehyde (2l) |
| --- |

| **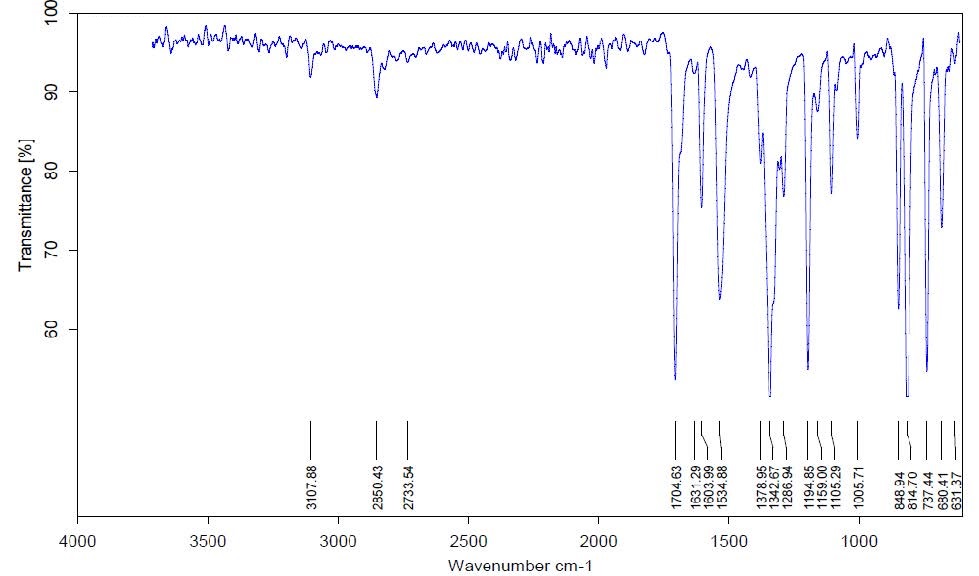**  **Fig. S30:** FT-IR spectrum of 4-Nitrobenzaldehyde (2n) |
| --- |

| **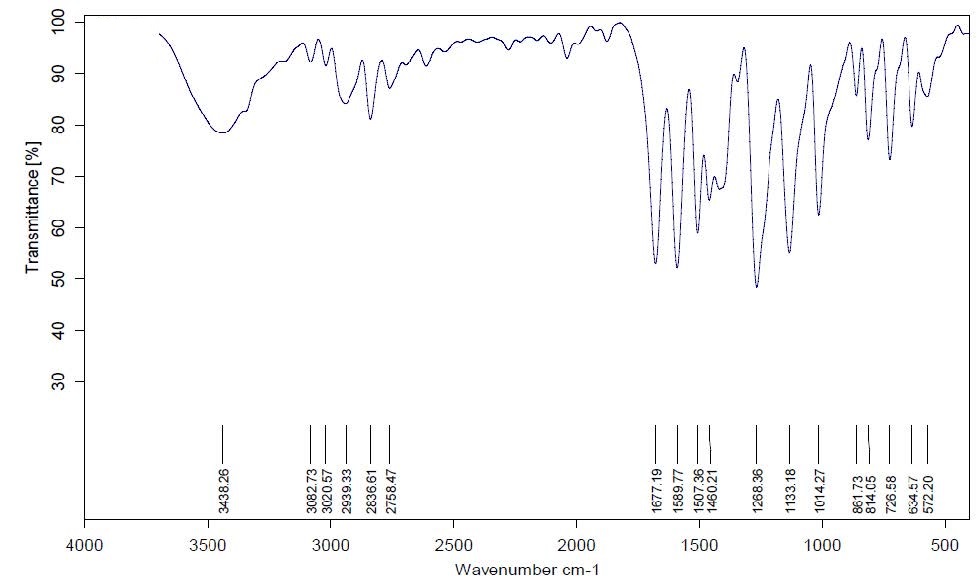**  **Fig. S31:** FT-IR spectrum of 3,4-Dimethoxybenzaldehyde (2o) |
| --- |

| **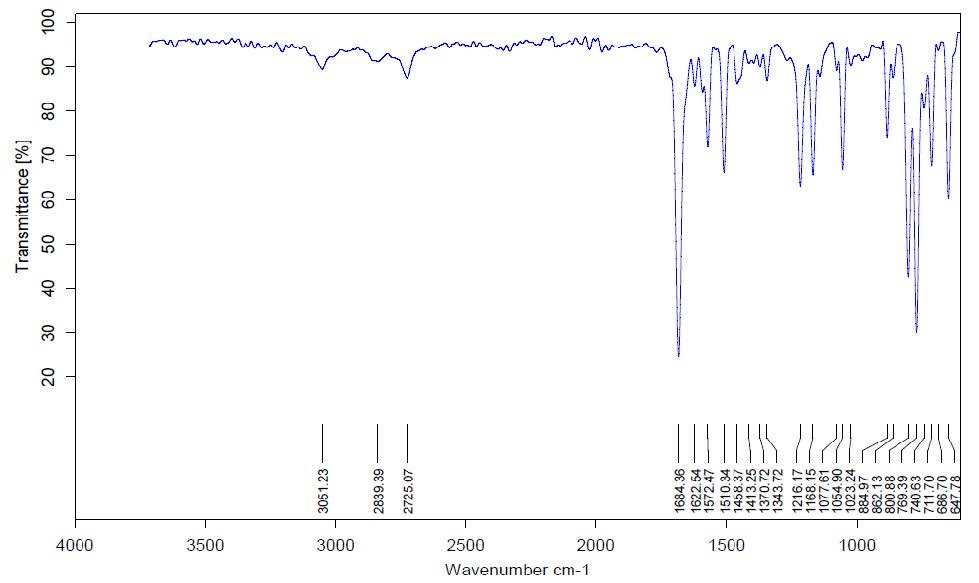**  **Fig. S32:** FT-IR spectrum of 1-Naphthaldehyde (2p) |
| --- |

| **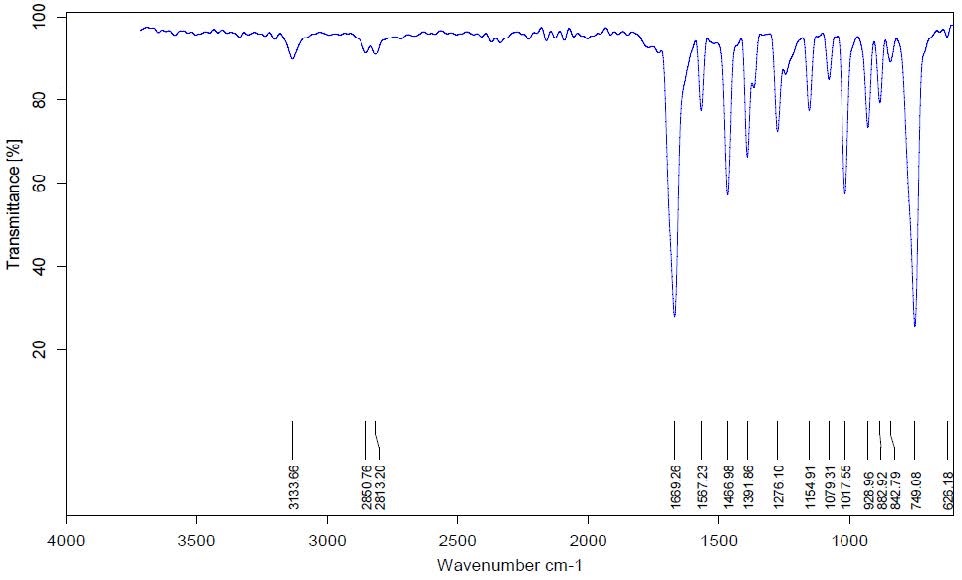**  **Fig. S33:** FT-IR spectrum of Furan-2-carbaldehyde (2q) |
| --- |

**References**

1. Nikitas, N. F., Tzaras, D. I., Triandafillidi, I., & Kokotos, C. G. (2020). Photochemical oxidation of benzylic primary and secondary alcohols utilizing air as the oxidant. Green Chemistry, 22(2), 471-477.
2. Könning, D., Olbrisch, T., Sypaseuth, F. D., Tzschucke, C. C., & Christmann, M. (2014). Oxidation of allylic and benzylic alcohols to aldehydes and carboxylic acids. Chemical Communications, 50(39), 5014-5016.
3. Hill, N. J., Hoover, J. M., & Stahl, S. S. (2013). Aerobic alcohol oxidation using a copper (I)/TEMPO catalyst system: a green, catalytic oxidation reaction for the undergraduate organic chemistry laboratory. Journal of Chemical Education, 90(1), 102-105.
4. Li, X. L., Zhang, R. Z., Niu, K. K., Dong, R. Z., Liu, H., Yu, S. S., ... & Xing, L. B. (2023). Construction of an efficient artificial light-harvesting system based on hyperbranched polyethyleneimine and improvement of photocatalytic performance. Chemical Communications, 59(89), 13301-13304.
5. Zhao, B., Shang, R., Cheng, W. M., & Fu, Y. (2018). Decarboxylative formylation of aryl halides with glyoxylic acid by merging organophotoredox with palladium catalysis. Organic Chemistry Frontiers, 5(11), 1782-1786.
6. Larrea, E. S., Fernández de Luis, R., Orive, J., Iglesias, M., & Arriortua, M. I. (2015). Mixed metal-organic framework as a heterogeneous catalyst.
7. Gao, M., Gan, Y., & Xu, B. (2019). From alkenes to isoxazolines via copper-mediated alkene cleavage and dipolar cycloaddition. Organic Letters, 21(18), 7435-7439.
8. Zarei, M., Noroozizadeh, E., Moosavi-Zare, A. R., & Zolfigol, M. A. (2018). Synthesis of nitroolefins and nitroarenes under mild conditions. The Journal of Organic Chemistry, 83(7), 3645-3650.
